# Structural color generation: from layered thin films to optical metasurfaces

**DOI:** 10.1515/nanoph-2022-0063

**Published:** 2023-02-22

**Authors:** Danyan Wang, Zeyang Liu, Haozhu Wang, Moxin Li, L. Jay Guo, Cheng Zhang

**Affiliations:** School of Optical and Electronic Information & Wuhan National Laboratory for Optoelectronics, Huazhong University of Science and Technology, Wuhan, Hubei 430074, China; Department of Electrical Engineering and Computer Science, University of Michigan, Ann Arbor, MI 48109, USA

**Keywords:** structural color, optical metasurface, Fabry Pérot resonance, guided mode resonance, plasmon resonance, Mie resonance

## Abstract

Recent years have witnessed a rapid development in the field of structural coloration, colors generated from the interaction of nanostructures with light. Compared to conventional color generation based on pigments and dyes, structural color generation exhibits unique advantages in terms of spatial resolution, operational stability, environmental friendliness, and multiple functionality. Here, we discuss recent development in structural coloration based on layered thin films and optical metasurfaces. This review first presents fundamentals of color science and introduces a few popular color spaces used for color evaluation. Then, it elaborates on representative physical mechanisms for structural color generation, including Fabry–Pérot resonance, photonic crystal resonance, guided mode resonance, plasmon resonance, and Mie resonance. Optimization methods for efficient structure parameter searching, fabrication techniques for large-scale and low-cost manufacturing, as well as device designs for dynamic displaying are discussed subsequently. In the end, the review surveys diverse applications of structural colors in various areas such as printing, sensing, and advanced photovoltaics.

## 1 Introduction

Color is an important part of our everyday life, for both aesthetic and social reasons [1–4]. The most frequently used method to create color is based on light absorbing materials such as organic dyes and chemical pigments [5–8]. A portion of visible light is absorbed by these materials and the remaining part is reflected back (or transmitted through), imparting color to an object. Dyes and pigments have been widely employed in diverse applications such as painting, displaying, cloth coloring, etc. Unfortunately, these materials are generally chemically-unstable and can degrade when exposed to heat or ultraviolet (UV) illumination, restraining the lifetime and stability of generated colors. For some emerging applications such as advanced color display and spectral imaging, color filters based on dyes and pigments are not suitable due to their limited spatial resolution, spectral purity, and operational efficiency. Moreover, production of dyes and pigments might involve toxic raw materials and waste products, making itself an environmentally unfriendly process.

Despite the afore-mentioned mechanism, colors can also be generated from the interaction between nanostructures and light, a process typically referred to as structural coloration [9–16]. Vivid structural colors are found in the feathers and scales of many animals, such as peacocks, beetles, and Morpho butterflies [17–19], as well as employed in various ancient artworks, such as the Lycurgus Cup and stained glasses [20–22]. Compared to conventional color generation based on pigments and dyes, structural color generation exhibits unique advantages such as high spatial resolution, long-term operational stability, and environmental friendliness. With the advance in computational electromagnetics and nanofabrication technology over recent years, researchers are able to design and fabricate an array of structural color devices with diverse working principles, constituent materials, and spectral responses [23–28]. These devices have shown great application potentials in a plethora of areas such as imaging, displaying, sensing, energy harvesting, etc.

**Figure 1:**
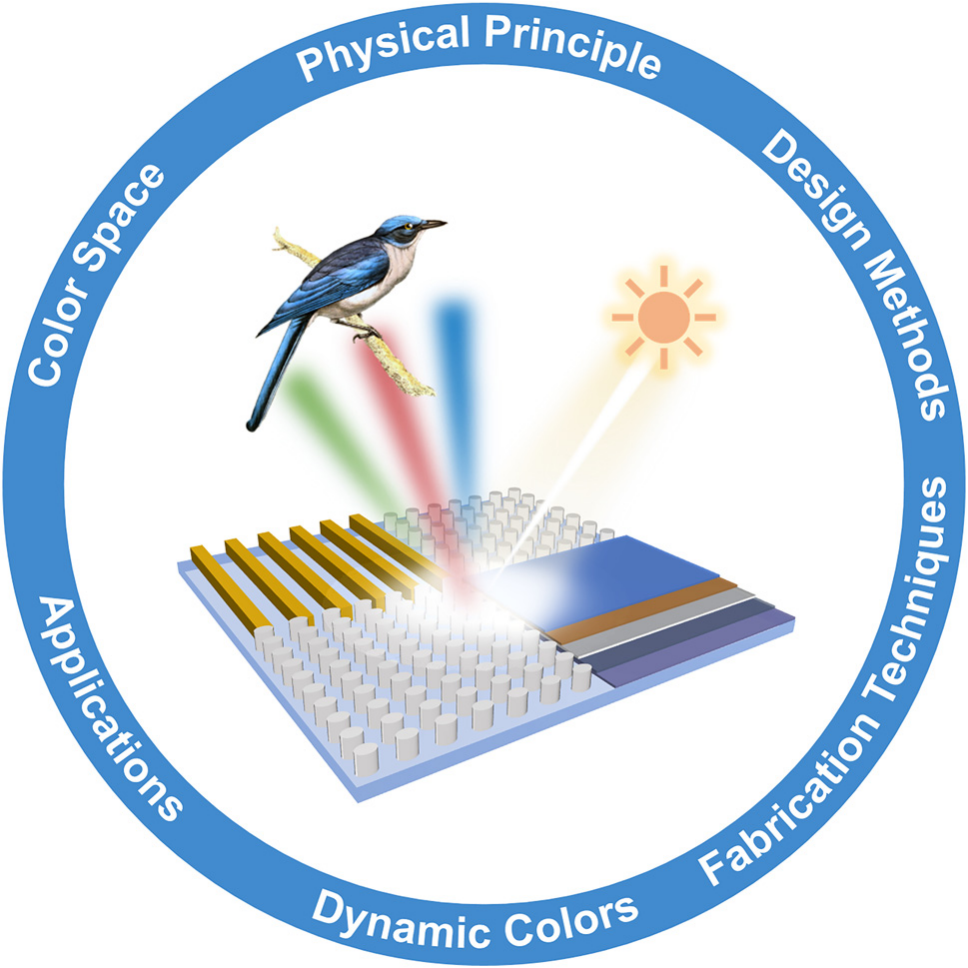
Schematic of structural color generation and key aspects covered by this review.

Here, we aim to systematically summarize the recent development of structural color generation that is based on layered thin films and optical metasurfaces. Optical metasurfaces are thin layers of nanostructures with engineered electromagnetic responses [29–33]. With properly designed and arranged subwavelength structures, a metasurface enables versatile amplitude, phase, and polarization manipulations of an incident electromagnetic wave [34–39]. In this review, we first introduce fundamental knowledge of color science and popular color spaces used for understanding and evaluating structural colors. Then, we elaborate on several key physical mechanisms and the associated device structures. Representative device designs that are based on either stacked multilayer thin films (e.g., Fabry–Pérot cavities and photonic crystals) or planar subwavelength nanostructure arrays (e.g., metallic and dielectric resonators) are discussed in details. Optimization methods for efficient design parameter searching, fabrication techniques for large-scale and low-cost manufacturing, as well as techniques for dynamic color tuning are surveyed subsequently. We conclude this review by discussing both classical and emerging applications of structural colors, as well as providing our perspectives on the future development of nanostructure-based structural coloration technology (Figure 1).

## 2 Fundamentals of color science and color spaces

**Figure 2:**
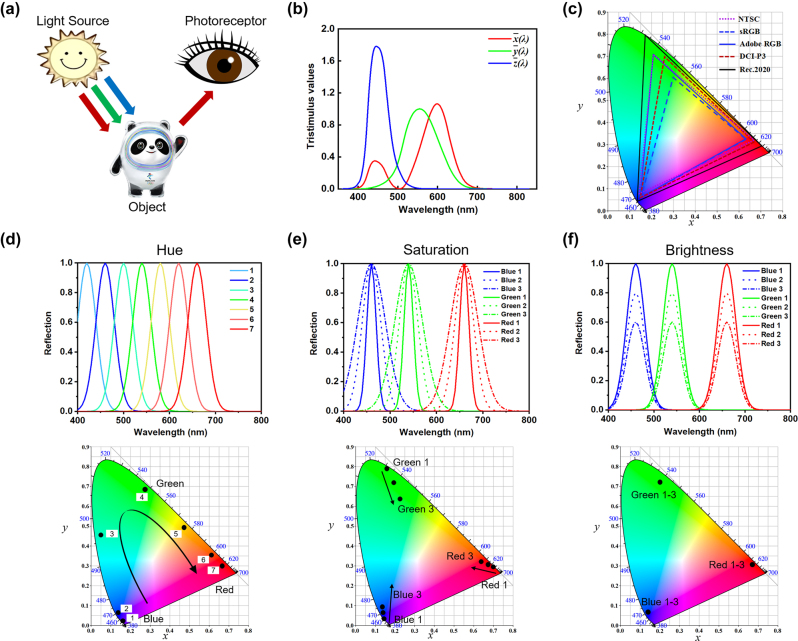
CIE 1931 *XYZ* color space. (a) Schematic diagram of the color perception process, where the object (Bing Dwen Dwen) reflects, transmits, or absorbs certain amount of the incident EM waves through various physical and chemical processes. The subsequently reflected (transmitted) light is received by the photoreceptors on human retina, generating signals to the visual cortex for the final color perception. (b) Color matching functions $\bar{x}\left(\lambda \right)$ , $\bar{y}\left(\lambda \right)$ and $\bar{z}\left(\lambda \right)$ . (c) The CIE 1931 *XYZ* chromaticity diagram and five popular color gamut standards. (d) Reflection spectra with different central wavelengths (upper panel) and the corresponding coordinates in the CIE *XYZ* color space (lower panel) to illustrate the hue property of a color. (e) Reflection spectra with different bandwidths (upper panel) and the corresponding coordinates in the CIE *XYZ* color space (lower panel) to illustrate the saturation property of a color. (f) Reflection spectra with different peak intensities (upper panel) and the corresponding coordinates in the CIE *XYZ* color space (lower panel) to illustrate the brightness property of a color.

Color itself is not a physical parameter, but rather a subjective perception of an individual observer through a complex visual process [40–42]. When the electromagnetic (EM) radiation from a light source illuminates an object, it will reflect, transmit, or absorb certain amount of the incident EM waves through various physical and chemical processes. The subsequently reflected (transmitted) light is then received by the photoreceptors on human retina, generating signals to the visual cortex for the final color perception (Figure 2(a)). Therefore, human color perception depends on various factors including the EM spectrum of the illumination source, the EM properties of the object, as well as the response functions of the observer’s eye and brain.

Generally speaking, human eyes are sensitive to incident light with free-space wavelength roughly between 400 nm (blue) and 800 nm (red). So, the reflection, transmission, and absorption properties of an object within the above wavelength range (commonly referred to as the visible range) play a critical role in its generated color, and are the focus of most structural color research. Human color perception can be described by several color appearance parameters including hue, saturation, and brightness [43–46]. Hue refers to the color family of a specific color (e.g., red, green, blue, an intermediate color, etc.), and is determined by the color’s dominant wavelength over the visible spectrum. Saturation, also known as purity, indicates the monochromatic level of a specific color. The higher saturation a color shows, the less white-light component it contains. Brightness refers to how much light appears to be shone from the object. It describes the intensity level of a color.

For various practical applications such as paint production, color printing, and digital display, it is essential to build a mathematical relation between the eye-received visible EM spectrum and the corresponding physiologically perceived colors by human. Driven by such need, different color spaces are proposed to quantify the color’s properties [47–50]. For example, the CIE 1931 color spaces (including the RGB color space and the *XYZ* color space) designed by the International Commission on Illumination (CIE) in 1931 are the first defined quantitative links between the visible EM spectra and the human physiologically perceived colors. The CIE 1931 *XYZ* color space is much more convenient for chromaticity calculation compared with the CIE 1931 RGB color space due to the absence of negative tri-stimulus values [51–53]. To provide a more uniform color spacing, the CIE 1976 L^*^a^*^b^*^ color space was then designed in 1976 [54–56]. In the following part, we will elaborate on two popular color spaces, the CIE 1931 *XYZ* and the CIE 1976 L^*^a^*^b^*^, which are widely used in the design and evaluation of different structural colors.

### 2.1 CIE 1931 *XYZ* color space

The CIE 1931 *XYZ* color space is developed according to the color matching experiments containing all color sensations perceivable by a person with average vision [57, 58]. The tristimulus values, typically represented by *X*, *Y*, and *Z* coordinates, can be obtained by solving the corresponding integral equations (Eq. (1)) consisting of the color matching functions ( $\bar{x}\left(\lambda \right)$ , $\bar{y}\left(\lambda \right)$ and $\bar{z}\left(\lambda \right)$ , Figure 2(b)) [53, 59], relative spectral power distribution of the illuminating light source (*I*(*λ*)), and reflection or transmission spectrum of an object (*R*(*λ*)).

(1) $$\left\{\begin{array}{c} X=\frac{1}{k}\int_{\lambda_{1}}^{\lambda_{2}}\bar{x}\left(\lambda \right)I\left(\lambda \right)R\left(\lambda \right)d\lambda \;\\ Y=\frac{1}{k}\int_{\lambda_{1}}^{\lambda_{2}}\bar{y}\left(\lambda \right)I\left(\lambda \right)R\left(\lambda \right)d\lambda \;\\ Z=\frac{1}{k}\int_{\lambda_{1}}^{\lambda_{2}}\bar{z}\left(\lambda \right)I\left(\lambda \right)R\left(\lambda \right)d\lambda \; \end{array}\right.$$

Where $\left[\lambda_{1},\lambda_{2}\right]$ is the spectrum range under study, and *k* is a normalizing factor defined as:

(2) $$k=\int_{\lambda_{1}}^{\lambda_{2}}\bar{y}\left(\lambda \right)I\left(\lambda \right)d\lambda$$

Standard illuminants D65, C, and E are the most frequently used light sources. D65 is a statistical representation of an average daylight with an associated color temperature of ∼6500 K. Illuminant C represents a daylight simulator with color temperature of ∼6774 K. Illuminant E is an equal-energy radiator with a color temperature of ∼5455 K.

The CIE 1931 *XYZ* chromaticity diagram (Figure 2(c)) is created as a perceptive color map that can contrast different color stimuli. Each point in the diagram denotes a certain color and the associated coordinate (*x*, *y*) can be obtained based on Eq. (3).

(3) $$\left\{\begin{array}{c} x=\frac{X}{X+Y+Z}\;\\ y=\frac{Y}{X+Y+Z}\;\\ z=\frac{Z}{X+Y+Z}\; \end{array}\right.$$

The color gradually changes from blue to green, and then to red when moving the coordinate point from the left bottom corner to the right bottom corner clockwise in the 1931 *XYZ* chromaticity diagram. It is worth noting that different kinds of color occupy areas with different sizes in this diagram (non-uniform color spacing). Also, the coordinate points closer to the diagram’s outer edge refer to colors of higher saturation, while points closer to the central point refer to colors of lower saturation. In the extreme case, the central point (*x* = 0.33, *y* = 0.33) denotes a white color.

The color gamut is usually shown by an enclosed area of the output colors from a device on the chromaticity diagram. The larger area the color gamut occupies, the wider color range the device can display [60–63]. A few frequently used color gamut standards, including NTSC (National Television System Committee) [64], sRGB (standard red green blue) [65, 66], Adobe RGB [67, 68], DCI-P3 (Digital Cinema Initiative) [69], and Rec. 2020 (ITU-R Recommendation BT. 2020) [70], are listed in Table 1. The triangular areas enclosed by the primary coordinates of these color gamut standards are plotted on CIE 1931 *XYZ* chromaticity diagram (Figure 2(c)).

**Table 1:** Popular color gamut standards.

Color gamut standard	Primary coordinates in CIE *XYZ* color space			Establisher	Year
	R	G	B		
NTSC	(0.67, 0.33)	(0.21, 0.71)	(0.14, 0.08)	US FCC	1953
sRGB	(0.64, 0.33)	(0.30, 0.60)	(0.15, 0.06)	IEC	1999
Adobe RGB	(0.64, 0.33)	(0.21, 0.71)	(0.15, 0.06)	Adobe	1998
DCI-P3	(0.68, 0.32)	(0.27, 0.69)	(0.15, 0.06)	DCI	2010
Rec.2020	(0.71, 0.29)	(0.17, 0.80)	(0.13, 0.05)	ITU	2012

US FCC represents the Federal Communications Commission of the United States. IEC represents the International Electrotechnical Commission. Adobe represents the Adobe Systems Incorporated. DCI represents the Digital Cinema Initiatives. ITU represents the International Telecommunication Union.

Next, we will utilize the CIE 1931 *XYZ* chromaticity diagram to evaluate a group of colors that are based on different reflection spectra from the objects under study. For the ease of description, we choose to use a Gaussian-like distribution to construct the reflection spectrum from an object:

(4) $$f\left(\lambda \right)=A\exp\left(-\frac{{\left(\lambda -\lambda_{\text{c}}\right)}^{2}}{2\Delta \lambda^{2}}\right)$$

where, *A* represents the peak reflection intensity, λ refers to the incident wavelength, *λ*_c_ denotes the central wavelength of the reflection spectrum, and Δλ is the linewidth.

Three representative cases will be evaluated in the following discussion, which are (i) a group of reflection spectra with the same peak intensity and linewidth, but different central wavelengths; (ii) a group of reflection spectra with the same peak intensity and central wavelength, but different linewidths; and (iii) a group of reflection spectra with the same central wavelength and linewidth, but different peak intensities. In all cases, standard illuminant E is employed as the light source.

First, we will evaluate colors generated from a group of reflection spectra with the same peak intensity and linewidth, but different central wavelengths. The upper panel in Figure 2(d) plots seven different reflection spectra having identical reflection peak intensity (*A* = 1.0) and linewidth (Δλ = 20 nm), but different central wavelengths (*λ*_c_ = 420, 460, 500, 540, 580, 620, and 660 nm). As the central wavelength increases, the corresponding color coordinate point in the CIE 1931 *XYZ* diagram moves clockwise from the lower left corner to the upper left corner, and then to the lower right corner (Figure 2(d), lower panel). The associated color gradually varies from blue to green, and then to red. This indicates that the hue of a given color is largely determined by the central wavelength of its spectrum, and different areas in the CIE 1931 diagram correspond to colors of different hues.

Next, we will evaluate colors generated from reflection spectra with the same peak intensity and central wavelength, but different linewidths. Three different groups of reflection spectra are constructed (Figure 2(e), upper panel). For Group #1, #2, and #3, the peak intensities are all set as *A* = 1.0, and the central wavelengths are respectively set as *λ*_c_ = 460 nm, 540 nm, and 660 nm. Each group contains three reflection spectra of different linewidths (∆*λ* = 10 nm, 20 nm, 30 nm). As shown in the lower panel of Figure 2(e), the calculated color coordinate points from the same group occupy areas of similar hue on the CIE diagram. Moreover, colors associated with narrower reflection spectra are located closer to the edge of the diagram, exhibiting a higher saturation.

Finally, we will evaluate colors generated from reflection spectra with the same central wavelength and linewidth, but different peak intensities. Similar to the previous case, three groups of spectra are constructed (Figure 2(f), upper panel). For Group #1, #2, and #3, the linewidths are all set as ∆*λ* = 20 nm, and the central wavelengths are respectively set as *λ*_c_ = 460 nm, 540 nm, and 660 nm. Each group contains three reflection spectra of different peak intensities (*A* = 1.0, 0.8, and 0.6). As displayed in the lower panel of Figure 2(f), calculated color coordinates from the same group occupy identical point on the CIE diagram. Intuitively, colors associated with spectra of larger peak intensities should exhibit higher brightness levels. This indicates that the CIE 1931 chromaticity diagram is not capable of truthfully describing the brightness character of a color. In order to adequately describe all three characters (hue, saturation, and brightness) of a certain color, different color spaces need to be adopted. Next, we will describe one of those color spaces- the CIE 1976 L^*^a^*^b^*^ color space.

### 2.2 CIE 1976 L^*^a^*^b^*^ color space

**Figure 3:**
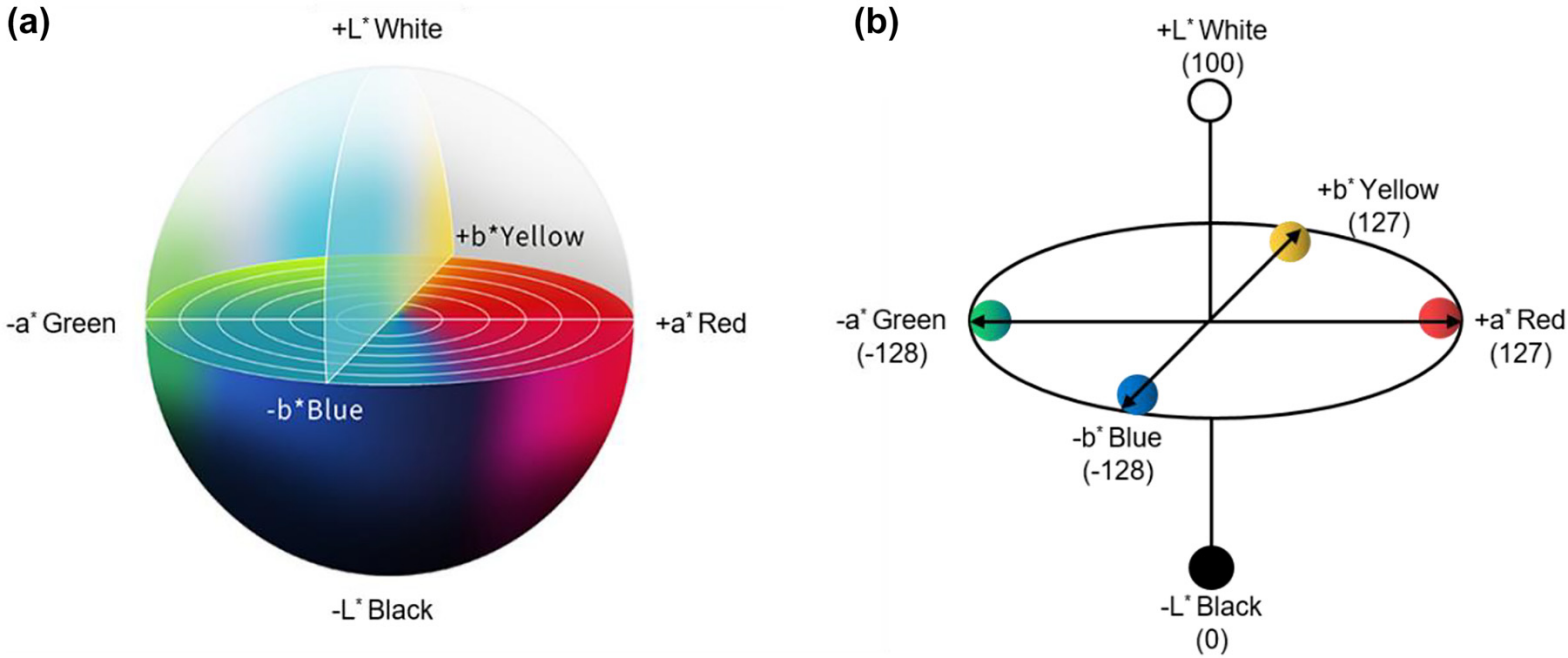
CIE 1976 L^*^a^*^b^*^ color space. (a) The CIE 1976 L^*^a^*^b^*^ chromaticity diagram. Modified with permission [71]. Copyright 2014, Multidisciplinary Digital Publishing Institute. (b) The L^*^, a^*^, and b^*^ values in the CIE 1976 L^*^a^*^b^*^ color space. L^*^ represents the color’s brightness level, whose value varies from 0 to 100 as the brightness level increases from black to white. a^*^ and b^*^ represent the color’s chromaticity level, whose values range from −128 to 127 as the chromaticity level shifts from green to red alone the a* axis and from blue to yellow along the b* axis.

The CIE 1976 L^*^a^*^b^*^ chromaticity diagram (Figure 3(a)) provides a 3D color map that enables an accurate measurement and comparison of all perceivable colors using three variables (i.e., L^*^, a^*^, and b^*^). Different from the CIE 1931 *XYZ* color space, the L^*^a^*^b^*^ color space can properly represent all three characters (hue, saturation, and brightness) of a given color with a uniform color spacing. In other words, Euclidean distance between coordinate points in the L^*^a^*^b^*^ color space roughly corresponds to the amount of change humans perceive between different colors. Any point in the L^*^a^*^b^*^ 3D coordinate system can be described by three variables, L^*^, a^*^, and b^*^, based on the following equations.

(5) $$\left\{\begin{array}{c} L^{*}=116f\left(\frac{Y}{Y_{n}}\right)-16\\ a^{*}=500\left[f\left(\frac{X}{X_{n}}\right)-f\left(\frac{Y}{Y_{n}}\right)\right]\\ b^{*}=200\left[f\left(\frac{Y}{Y_{n}}\right)-f\left(\frac{Z}{Z_{n}}\right)\right] \end{array}\right.$$

where

(6) $$f\left(t\right)=\left\{\begin{array}{cc} t^{1/3}\; & t>{\left(\frac{6}{29}\right)}^{3}\\ 7.787t+0.138\; & t\leq {\left(\frac{6}{29}\right)}^{3} \end{array}\right.$$

*X*, *Y*, and *Z* are the three tristimulus values obtained according to Eq. (1). *X*_*n*_, *Y*_*n*_, and *Z*_*n*_ describe a specified white achromatic reference illumination, and their values are associated with the employed white achromatic reference illuminant source. For the commonly used standard illuminant D65, *X*_*n*_, *Y*_*n*_, and *Z*_*n*_ are set as 95.0489, 100, and 108.884, respectively.

The L^*^, a^*^, and b^*^ values in the CIE 1976 L^*^a^*^b^*^ color space correspond to different characters of a given color. L^*^ represents the color’s brightness level, whose value varies from 0 to 100 as the brightness level increases from black to white. a^*^ and b^*^ represent the color’s chromaticity level, whose values range from −128 to 127 as the chromaticity level shifts from green to red alone the a^*^ axis and from blue to yellow along the b^*^ axis (Figure 3(b)). For colors having larger L^*^ values, the associated coordinate points are located on horizontal planes that are closer to the north pole in the L^*^a^*^b^*^ color space. In extreme cases, the coordinate point for a purely white color is located on the north pole (L^*^ = 100), while that for a purely black color is located on the south pole (L^*^ = 0). Colors with the same L^*^ values are located on the same horizontal plane. On a given horizontal plane, the distance from the center point represents the saturation level, or purity of a color. Coordinate points that are further away from the center point represent colors with higher saturation.

## 3 Physical principles

Vivid structural colors can be generated from either stacked planar thin films or subwavelength optical metasurfaces [23, 24, 72, 73]. Constructive or destructive interference effects can be engineered to take place in multilayer thin film stacks, which are commonly modeled as Fabry–Pérot (FP) cavities or one-dimensional (1D) photonic crystals, leading to peaks (dips) in the structures’ transmission (reflection) spectra [74–79]. The generated colors can be tuned by adjusting the total number and arrangement of the constituent layers, as well as the material and thickness of each layer. Structural colors based on stacked multi-layer thin films have advantages of low cost, high scalability, and compatibility with mass production. The bright colors seen in an oil slick or a soap bubble are examples of thin-film based structural colors in nature.

Another method to generate structural colors is to exploit interaction between light and subwavelength nanostructure, which supports various resonance effects including guided mode resonance [80–84], plasmon resonance [85–87], Mie resonance [88, 89], etc. The generated colors can be tuned by adjusting the constituent materials and geometry parameters (e.g., height, period, lateral size, etc.) of the nanostructures. Compared to structural colors based on thin film stacks, structural colors generated from subwavelength nanostructures exhibit fine spatial resolution (can be beyond the light diffraction limit) and higher degree of design freedom. The vivid colors seen in the peacock feather or neon tetra fish scale are examples of nanostructure-based structural colors in nature.

In the following part, we will elaborate on several key physical mechanisms employed for structural color generation. We will first discuss design methods using multilayer thin film stacks, which include FP cavity and 1D photonic crystal. Then, we will analyze resonance effects based on light–matter interaction inside nanostructures, which are categorized into guided mode resonance, plasmon resonance, and Mie resonance.

### 3.1 Thin-film-stack-based structural color generation

Transfer matrix method (TMM) [90–92] is one of the popular methods for analyzing the electromagnetic responses of a multilayer thin film structure, where 2 × 2 matrices are used to describe the light reflection (transmission) properties of stratified layers made of homogeneous materials.

**Figure 4:**
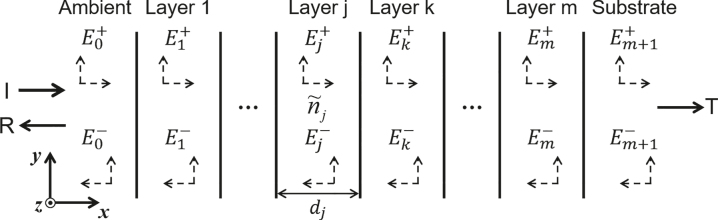
Schematic drawing of a multilayer thin film structure. The structure consists of m layers and is sandwiched between an ambient environment (layer 0) and a substrate (layer *m* + 1), both of which are considered to be semi-infinite. For layer *j* inside the thin film stack, its thickness is denoted as d_j_ and the complex index of refraction is represented by ${\tilde{n}}_{j}$ . The electrical field components propagating along the +*x* and −*x* directions are denoted as $E_{j}^{+}$ and $E_{j}^{-}$ , respectively. *I* represents the incident intensity, *R* and *T*, respectively denote the reflection and transmission intensity. Modified with permission [92]. Copyright 1999, American Institute of Physics.

Figure 4 shows the schematic drawing of a multilayer structure with m layers sandwiched between a semi-infinite ambient medium (layer 0) and a semi-infinite substrate (layer *m* + 1). A plane wave is incident from the ambient medium along the +*x* direction. For layer *j*, its thickness is denoted as *d*_*j*_, and the complex index of refraction is represented by ${\tilde{n}}_{j}$ . The electrical field components at the input (ambient side), $E_{0}^{+}$ and $E_{0}^{-}$ , are related to the electrical field components at the output (substrate side), $E_{m+1}^{+}$ and $E_{m+1}^{-}$ , by the transfer matrix *M*:

(7) $$\left[\begin{array}{c} E_{0}^{+}\\ E_{0}^{-} \end{array}\right]=M\left[\begin{array}{c} E_{m+1}^{+}\\ E_{m+1}^{-} \end{array}\right]$$

where *M* can be written as:

(8) $$M=\left[\begin{array}{cc} M_{11} & M_{12}\\ M_{21} & M_{22} \end{array}\right]=\left(\overset{m}{\underset{v=1}{\prod }}I_{\left(v-1\right)v}L_{v}\right)\cdot I_{m\left(m+1\right)}$$

Here, *L*_*j*_ is a layer matrix describing the light propagation through layer *j*, and is described by:

(9) $$L_{j}=\left[\begin{array}{cc} {\text{e}}^{-i\xi_{j}d_{j}} & 0\\ 0 & {\text{e}}^{\text{i}\xi_{j}d_{j}} \end{array}\right]$$

where

(10) $$\xi_{j}=\frac{2\pi }{\lambda }\sqrt{{\tilde{n}}_{j}^{2}-n_{0}^{2}\sin\theta }$$

*n*_0_ is the refractive index of the ambient medium (layer 0), and *θ* is the angle of incidence on the input side (layer 0).

*I*_*jk*_ is an interface matrix describing light reflection and transmission at the interface between layer *j* and *k*, and is given by:

(11) $$I_{jk}=\frac{1}{t_{jk}}\left[\begin{array}{cc} 1 & r_{jk}\\ r_{jk} & 1 \end{array}\right]$$

For an incident light with transverse electric (TE) polarization (electric field perpendicular to the plane of incidence), the reflection and the transmission coefficients, *r*_*jk*_ and *t*_*jk*_, are expressed as:

(12) $$r_{jk}^{\text{TE}}=\frac{\sqrt{{\tilde{n}}_{j}^{2}-n_{0}^{2}\sin\theta }-\sqrt{{\tilde{n}}_{k}^{2}-n_{0}^{2}\sin\theta }}{\sqrt{{\tilde{n}}_{j}^{2}-n_{0}^{2}\sin\theta }+\sqrt{{\tilde{n}}_{k}^{2}-n_{0}^{2}\sin\theta }}$$

(13) $$t_{jk}^{\text{TE}}=\frac{2\sqrt{{\tilde{n}}_{j}^{2}-n_{0}^{2}\sin\theta }}{\sqrt{{\tilde{n}}_{j}^{2}-n_{0}^{2}\sin\theta }+\sqrt{{\tilde{n}}_{k}^{2}-n_{0}^{2}\sin\theta }}$$

For an incident light with transverse magnetic (TM) polarization (electric field parallel to the plane of incidence), *r*_*jk*_ and *t*_*jk*_ are expressed as:

(14) $$r_{jk}^{\text{TM}}=\frac{{\tilde{n}}_{k}^{2}\sqrt{{\tilde{n}}_{j}^{2}-n_{0}^{2}\sin\theta }-{\tilde{n}}_{j}^{2}\sqrt{{\tilde{n}}_{k}^{2}-n_{0}^{2}\sin\theta }}{{\tilde{n}}_{k}^{2}\sqrt{{\tilde{n}}_{j}^{2}-n_{0}^{2}\sin\theta }+{\tilde{n}}_{j}^{2}\sqrt{{\tilde{n}}_{k}^{2}-n_{0}^{2}\sin\theta }}$$

(15) $$t_{jk}^{\text{TM}}=\frac{2{\tilde{n}}_{j}{\tilde{n}}_{k}\sqrt{{\tilde{n}}_{j}^{2}-n_{0}^{2}\sin\theta }}{{\tilde{n}}_{k}^{2}\sqrt{{\tilde{n}}_{j}^{2}-n_{0}^{2}\sin\theta }+{\tilde{n}}_{j}^{2}\sqrt{{\tilde{n}}_{k}^{2}-n_{0}^{2}\sin\theta }}$$

Consider that when light is incident from the ambient side, there is no electrical field component propagating along the −*x* direction on the substrate side ( $E_{m+1}^{-}=0$ ). Therefore, the complex reflection and transmission coefficients of the multilayer structure can be expressed as:

(16) $$r=\frac{E_{0}^{-}}{E_{0}^{+}}=\frac{M_{21}}{M_{11}}$$

(17) $$t=\frac{E_{m+1}^{+}}{E_{0}^{+}}=\frac{1}{M_{11}}$$

According to the Fresnel equations, the reflectivity *R* and transmittance *T* of the multilayer structure are:

(18) $$R=|r{|}^{2}$$

(19) $$T=\frac{\text{Re}\left({\tilde{n}}_{m+1}\cos\theta_{m+1}\right)}{n_{0}\cos\theta }|t{|}^{2}$$

Herein, ${\tilde{n}}_{m+1}$ is the refractive index of the substrate (layer *m* + 1), and *θ*_*m*+1_ is the refraction angle on the output side (layer *m* + 1).

**Fabry**–**Pérot cavity**. FP cavity, whose basic form consists of an optically transparent dielectric medium sandwiched by two metallic mirrors, is one of the simplest resonators for generating structural colors [93–96]. A wide range of colors can be created by simply adjusting the dielectric layer thickness.

**Figure 5:**
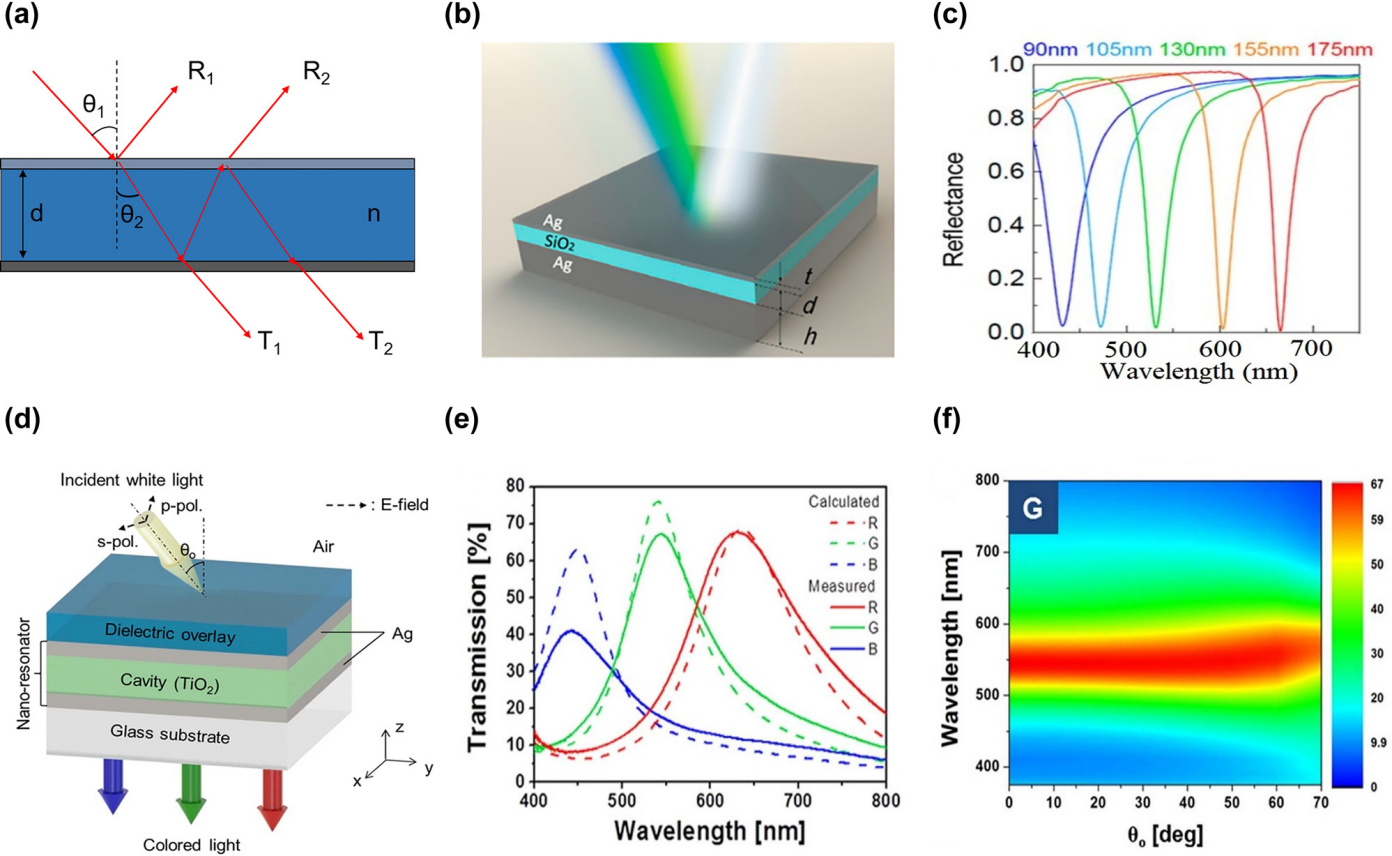
FP resonance for structural color generation. (a) Schematic diagram of a typical FP cavity model, which is composed of two metal mirrors and a dielectric layer between them. Here, *n* and *d* respectively represent refractive index and thickness of the dielectric layer. *θ*_1_ and *θ*_2_ represent angles of incidence and refraction at the metal/dielectric interface on the input side, respectively. (b) Schematic diagram of the Ag-SiO_2_-Ag based FP cavity. Here, *t*, *d*, and *h* represent thickness of the top Ag, the middle SiO_2_, and the bottom Ag layers, respectively. (c) Simulated reflectance spectra with the cavity length d ranging from 90 nm to 175 nm. The top and bottom Ag layer thicknesses are fixed as 30 and 100 nm, respectively. Modified with permission [101]. Copyright 2015, American Chemical Society. (d) Schematic diagram of the TiO_2_-Ag-TiO_2_-Ag based FP cavity for angle-robust structural color generation. (e) The calculated (dashed curves) and measured (solid curves) transmission spectra of the red, green and blue colors, where the TiO_2_ cavity lengths are set as 100 nm, 75 nm, and 50 nm, respectively. Thicknesses of the over-coated TiO_2_ layer, the top Ag layer, and the bottom Ag layer are respectively fixed as 60 nm, 23 nm, and 23 nm for all designs. (f) Angularly-resolved transmission spectra of the green-color device under p-polarized illumination. The device exhibits close-to-identical transmission spectra as the illumination angle increases up to 70°. Reproduced with permission [103]. Copyright 2015, Nature Publishing Group.

To gain a better understanding of its working mechanism, a standard FP cavity model is applied to analyze its operation [97]. The employed model is a symmetric FP cavity where a transparent dielectric layer is sandwiched between two identical metallic mirrors, and both the input and output sides have the same refractive indices (Figure 5(a)). Here, *n* and *d* denote the refractive index and thickness of the dielectric layer, respectively. For the ease of discussion, material absorption and dispersion of the dielectric layer is neglected. But the conclusions drawn from this simplified model also apply to more complicated cases where material absorption and dispersion exist.

The reflectivity *R*_FP_ as well as the transmittance *T*_FP_ of a symmetric FP cavity can be written as [98, 99]:

(20) $$R_{\text{FP}}=\frac{2R\cdot \left(1-\cos\delta \right)}{1+R^{2}-2R\cos\delta }$$

(21) $$T_{\text{FP}}=\frac{{\left(1-R\right)}^{2}}{1+R^{2}-2R\cos\delta }$$

where *R* is the reflection coefficient of the metal film, and *δ* is the total phase shift accumulated during a single round trip within the cavity [93, 100]. *δ* equals to *δ*_*a*_ + *δ*_*b*_ + *δ*_prop_, where *δ*_*a*_ and *δ*_*b*_ denote the reflection phase shifts associated with the metal–dielectric interfaces, and *δ*_prop_ is the round-trip propagation phase shift in the cavity:

(22) $$\delta_{\text{prop}}=\left(\frac{2\pi }{\lambda }\right)\cdot n\cdot 2d\cdot \cos\theta_{2}$$

where *θ*_2_ represents angle of refraction at the metal/dielectric interface. Compared to the propagation phase shift *δ*_prop_, the reflection phase shifts, *δ*_*a*_ and *δ*_*b*_, are relatively small in many cases. As a result, the total phase shift *δ* is mainly determined by *δ*_prop_. Reflection minima (transmission maxima) occurs at certain incident wavelengths when the associated phase shift between two adjacent reflected (transmitted) lights is an integral number of 2*π* radians (i.e., *δ* = 2*mπ*). Increasing the dielectric layer thickness (*d*) leads to an increase of the phase shift for a given angle of incidence (Eq. (22)), and therefore, induces a shift in the cavity’s resonant wavelength. Consequently, diverse colors (resonance spectra) across the entire visible band can be easily achieved by adjusting the dielectric layer thickness.

Li and co-workers propose a stacked three-layer design for FP-cavity-based structural color generation [101]. The device is composed of a silicon dioxide (SiO_2_) layer sandwiched by two silver (Ag) mirrors, with thicknesses of the top Ag, the middle SiO_2_, and the bottom Ag layers, respectively, labeled as *t*, *d*, and *h* (Figure 5(b)). Ag is chosen for the metallic layer by its low extinction coefficient and absence of inter-band transition in the visible range. SiO_2_ is chosen for the cavity layer by its high visible transparency. To construct reflection-mode devices, the bottom Ag layer is chosen to be optically opaque (*h* = 100 nm). By fixing the thickness of the top Ag layer (*t* = 30 nm) and adjusting that of the middle SiO_2_ layer (*d*) from 90 nm to 175 nm, reflectance dips with central wavelengths gradually varying from 450 nm to 650 nm are obtained (Figure 5(c)). Transmission-mode colors can also be realized by reducing the thickness of the bottom Ag layer to 30 nm while maintaining the same top Ag layer thickness. Five different transmission spectra are obtained by adjusting the SiO_2_ layer thickness (*d*) from 100 nm to 200 nm with a step of 25 nm. The obtained transmission resonance peaks are distributed across the whole visible range, and the measured curves exhibit a fair similarity to the simulated ones.

The afore-mentioned FP cavities generate pre-designed vivid colors under normal incidence. However, their resonant wavelengths exhibit a blue shift as the angle of incidence increases, leading to performance degradation and restricting the design’s usage in many displaying and imaging devices. One effective method to mitigate the angle-dependent issue of FP cavities is to employ high-refractive-index materials for the middle layer, such as silicon nitride (Si_3_N_4_) [102], titanium dioxide (TiO_2_) [103], and tantalum pentoxide (Ta_2_O_5_) [104], etc. Figure 5(d) illustrates an angle-robust, transmission-type structural color design having a four-layer structure of TiO_2_-Ag-TiO_2_-Ag [103]. The bottom three layers of Ag, TiO_2_ and Ag form an FP cavity with increased angular robustness. The thicknesses of both Ag layers are chosen to be 23 nm, while the thickness of the middle TiO_2_ layer is adjusted to generate different transmitted colors. An additional 60 nm-thick TiO_2_ layer is over-coated on top of the FP cavity to further alleviate the structure’s angle-sensitive response and simultaneously suppress light reflection from the structure. Transmission resonances with peak wavelengths of 636 nm, 541 nm, and 450 nm, which respectively correspond to eye-perceived red (R), green (G), and blue (B) colors, are achieved by setting the middle TiO_2_ layer thickness as 100 nm, 75 nm, and 50 nm, respectively (Figure 5(e)). For a designed green-color device, its transmission spectrum is characterized at different angles of incidence ranging from 0° to 70°. The experimentally measured spectra exhibit close-to-identical transmission peaks around 541 nm (Figure 5(f)) for p-polarized incident light, verifying the device’s angle-robust response.

The angle-dependent response of an FP cavity can be explained using the afore-mentioned resonator model. For an FP cavity shown in Figure 5(a), the resonant wavelength of the reflection (transmission) spectrum is expressed as: $\lambda_{r}=2d\cdot \sqrt{n^{2}-\text{si}{\text{n}}^{2}\theta_{1}}$ . The variation in the resonant wavelength with respect to the change in angle of incidence can be written as:

(23) $$\left|\frac{\Delta \lambda_{r}}{\Delta \theta_{1}}\right|\approx \frac{2d\cdot \sin\theta_{1}\cdot \cos\theta_{1}}{\sqrt{n^{2}-\text{si}{\text{n}}^{2}\theta_{1}}}$$

It can be seen that the angular sensitivity of the device’s reflection (transmission) spectrum is, on one hand, proportional to the thickness of the middle dielectric layer (*d*), and on the other hand, inversely-proportional to the index of refraction of the middle dielectric layer (*n*). As we have discussed earlier, employing a high-refractive-index dielectric layer can effectively facilitate the device’s angularly-robust response. Next, we will show how reducing the cavity length would benefit the device’s performance.

**Figure 6:**
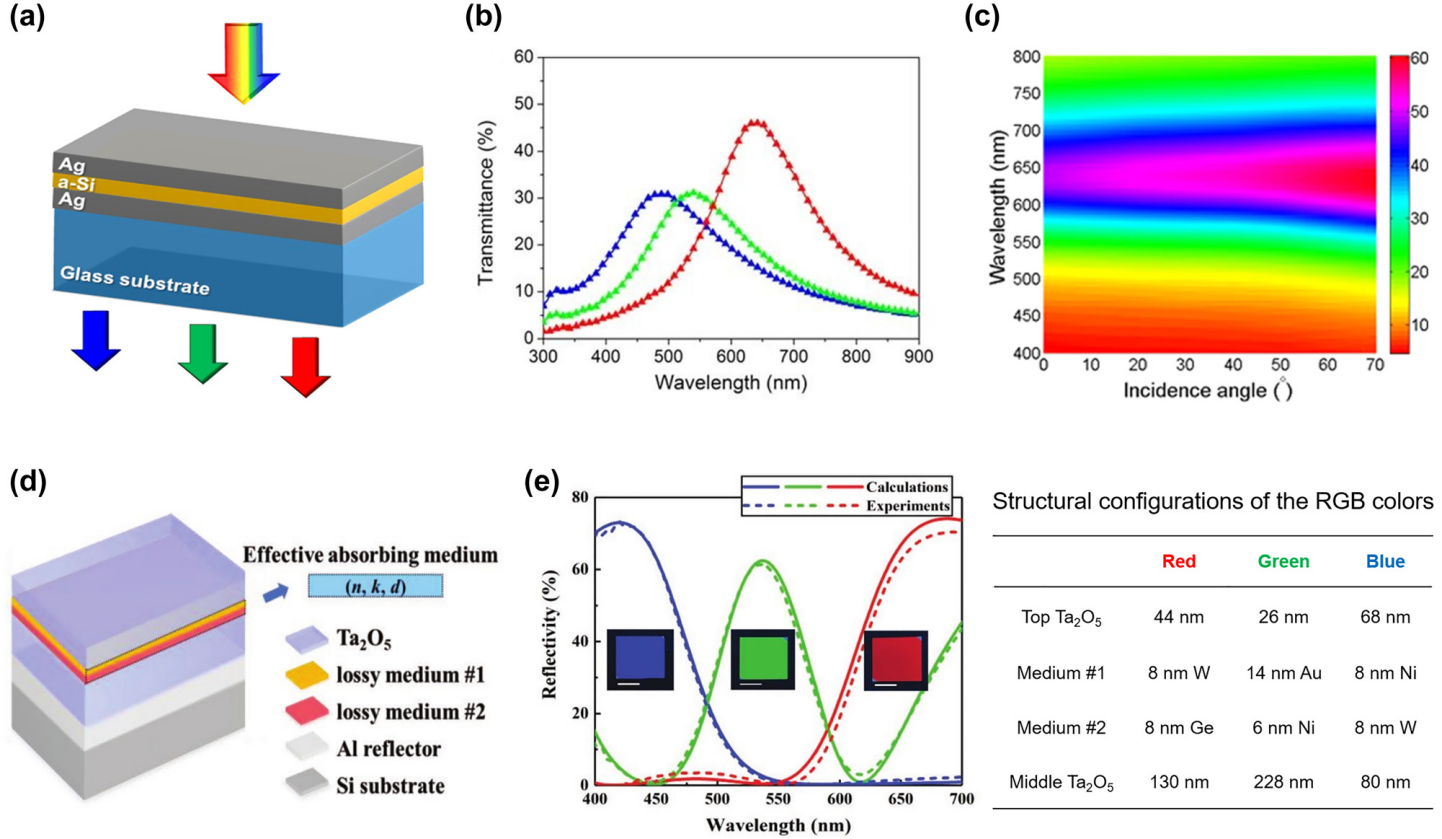
Absorbing material-based FP resonance for structural color generation. (a) Schematic diagram of the Ag/α-Si/Ag based FP cavity. (b) The measured transmission spectra of red, green, and blue colors under normal incidence. The α-Si layer thicknesses are, respectively, chosen as 28 nm (red), 15 nm (green), and 9 nm (blue). For all designs, the thicknesses of both the top and bottom Ag layers are set as 18 nm. (c) Measured angularly-resolved transmission spectra with the incident angle ranging from 0° to 70° under TM polarized illumination. Reproduced with permission [106]. Copyright 2014, American Institute of Physics. (d) Schematic diagram of the dielectric/bi-layer absorber/dielectric/metal (DADM) based FP cavity for high-purity additive color generation. (e) Left panel: simulated (solid curves) and measured (dashed curves) reflection spectra of the red, green, and blue colors. Insets: optical images of the fabricated RGB samples. Scale bars: 1.0 cm. Right panel: structural configurations of the three devices for RGB color generation. Modified with permission [104]. Copyright 2019, Wiley-VCH.

One simple yet effective approach to construct an FP resonator with reduced cavity length is to deposit an ultrathin semiconductor layer (i.e., light-absorbing dielectric layer) on top of a metallic substrate [105–107]. Under such configuration, light reflections at both the bottom metal/semiconductor interface and the top medium (usually air)/semiconductor interface will generate nontrivial (neither 0 nor *π*) phase shifts. These nontrivial reflection phase shifts, together with the propagation phase shift inside the cavity, lead to resonance effects occurring in the ultrathin semiconductor layer. Kats and co-workers demonstrate an array of angle-insensitive structural colors generated from highly-absorbing amorphous Ge layers (with thicknesses varying from 7 nm to 25 nm) deposited on Au substrates [107]. For every 1 nm change in the Ge layer thickness, the measured reflection spectrum exhibits a ∼20 nm shift in its dip position. Moreover, the absorption band maintains its position for illumination angles up to 60° for both TE and TM polarized illumination. Lee and co-workers also demonstrate angle-insensitive transmission colors by sandwiching a thin amorphous silicon (α-Si) layer between two optically thin Ag layers (Figure 6(a)) [106]. To create red, green, and blue transmission colors (Figure 6(b)), thickness of the α-Si layer is set to be 28 nm, 15 nm, and 9 nm, respectively. In all designs, the thicknesses of both top and bottom Ag layers are fixed as 18 nm. As the extinction coefficient of α-Si is lower than that of Ge over the visible range, the corresponding devices exhibit resonance spectra of narrower bandwidth and higher efficiency. Similar to the afore-mentioned design using Ge as a lossy dielectric layer, the Ag/α-Si/Ag structure exhibits angle-robust transmission colors (Figure 6(c)).

As can be seen from various studies mentioned earlier, an FP-cavity-based device typically generates subtractive colors (e.g., cyan, magenta, and yellow) in its reflection mode and additive colors (e.g., red, green, and blue) in transmission mode. This is due to the fact that resonance-induced absorption inside the FP cavity is usually narrowband. In order to realize additive reflection colors with high purity, constituent materials with high extinction coefficients are required for broadening the FP cavity’s absorption bandwidth. For such purpose, lossy metals (e.g., Ni, Ti, W, etc.) and semiconductors (e.g., Ge, α-Si, etc.) are typically chosen. For example, Yang and co-workers design asymmetric FP resonators to generate bright and pure additive reflection colors, which are based on a three-layer structure of Ni (6 nm), SiO_2_, and Al (100 nm) on Si substrate [108]. The thin Ni layer works as a broadband absorber and the thick Al layer as a bottom reflective mirror. By adjusting thickness of the SiO_2_ layer from 120 nm to 270 nm, different colors can be generated.

Yang and co-workers demonstrate another reflection-type device having a dielectric/bi-layer absorber/dielectric/metal (DADM) configuration (Figure 6(d)) to generate bright additive colors [104]. In this design, the FP cavity’s absorbing layer is made of two lossy media with different absorbing characteristics over the visible band. Combination of these two materials further enhances the device’s absorption bandwidth, and therefore, improves the reflection color’s purity. Moreover, the top dielectric layer acting as an antireflection (AR) coating can further reduce the intensity of undesired sideband reflection. A 100 nm-thick Al bottom mirror is used to provide high reflection and prevent light transmission, and loss-less Ta_2_O_5_ with high refractive index is used as both the dielectric layer and AR layer to promote the device’s angular insensitivity. RGB colors are achieved by properly choosing the two absorbing media and adjusting the thickness of each layer (Figure 6(e)).

**Figure 7:**
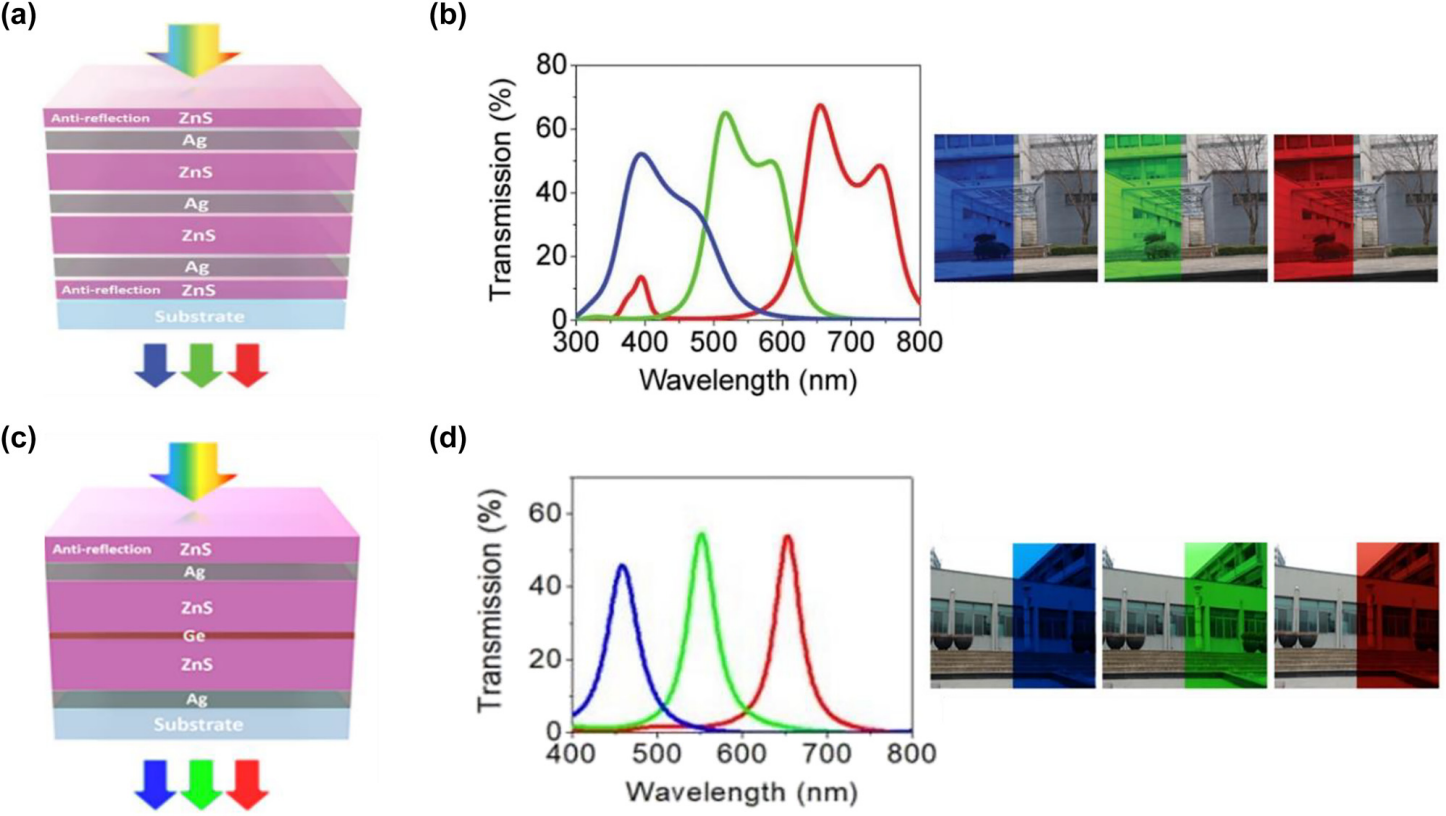
Dual-FP cavity resonance for structural color generation. (a) Schematic representation of the dual-FP cavity with a ZnS antireflection (AR) coating on each side, where each cavity consists of two optically thin Ag films and a ZnS layer. (b) Measured transmission spectra (left panel) and optical images (right panel) of the fabricated samples. For each red (R), green (G), and blue (B) color device, thickness of the ZnS (Ag) layer is chosen as 104 (32), 70 (34), and 40 (28) nm, respectively. For each device, thickness of the ZnS AR coating is set as half of that of the ZnS layer inside the FP cavity. Modified with permission [110]. Copyright 2017, Wiley-VCH. (c) Schematic diagram of transmission-type structural color generation with a dual-FP cavity. (d) Measured transmission spectra (left panel) and optical images (right panel) of the fabricated samples. The thickness of each layer from top to bottom is chosen as 55 nm/30 nm/225 nm/13 nm/30 nm for the red-color device, 45 nm/35 nm/185 nm/5 nm/35 nm for the green-color device, and 35 nm/35 nm/140 nm/5 nm/35 nm for the blue-color device. Modified with permission [76]. Copyright 2019, Nature Publishing Group.

Generally speaking, it is difficult to obtain structural colors with both high brightness and high purity using a single FP cavity, due to the intrinsic tradeoff between these two performance parameters and the limited degree of design freedom for single-FP-cavity devices. For example, the reflection (or transmission) intensity of off-resonance wavelength components needs to be suppressed as much as possible for improving the color purity. This can be achieved by increasing the thicknesses of absorbing layers or choosing materials with higher extinction coefficients. Unfortunately, the intensity of resonant peaks will be sacrificed at the same time, leading to degradation of the color brightness. To mitigate such issue, dual FP cavity-based devices have been exploited [76, 109, 110]. Lee and co-workers demonstrate efficient high-purity structural colors by employing a dual FP cavity [110]. The proposed device is composed of two cascaded identical FP cavities with metal–dielectric–metal structures, as well as a dielectric antireflection (AR) coating on each side (Figure 7(a)). Each FP cavity consists of two optically-thin Ag films and a zinc sulfide (ZnS) layer. Measured spectra under normal incidence display transmission peaks with efficiencies over 50% and off-resonance sidebands with largely suppressed intensities (Figure 7(b)). The associated images of fabricated samples display RGB colors with both high purity and brightness.

Another dual-cavity design of improved color purity using the high-order resonance suppression approach is proposed [76]. Figure 7(c) illustrates the schematic of the proposed device, which consists of an antireflection coating (ZnS) and a “compound dielectric layer” switched by two thin Ag mirrors, where the “compound dielectric layer” is made of ZnS, Ge, and ZnS. The designed FP cavity in the form of ZnS/Ag/ZnS/Ag/substrate has a relatively long cavity length, and thus, supports several high-order resonance modes which exhibit narrower linewidths compared to the fundamental resonance mode. Inserting a thin but highly-absorbing Ge layer into the cavity effectively eliminates the 5th order resonance mode (which occurs at the shorter wavelength range), while at the same time, barely affects the 3rd order FP resonance. Figure 7(d) displays three measured transmission spectra under normal incidence, which all exhibit sharp resonances with peak efficiencies over 50% as well as greatly suppressed sidebands. Photos of the fabricated devices exhibit red, green, and blue colors with fairly high purity.

**Figure 8:**
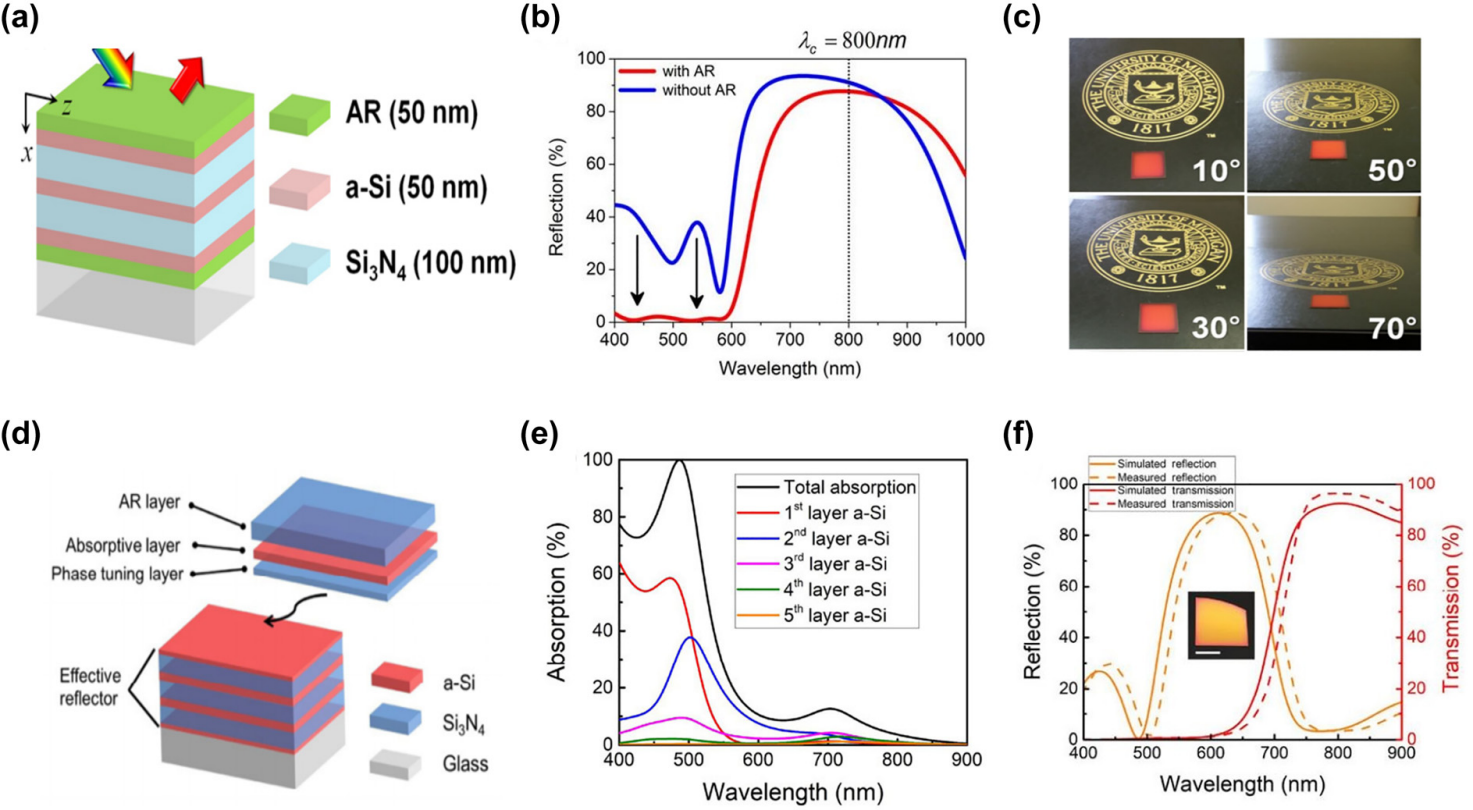
1D photonic crystal resonance for structural color generation. (a) Schematic diagram of the proposed 1D photonic crystal for generating red color, which consists of alternating layers of α-Si and Si_3_N_4_ with a dielectric (Si_3_N_4_) AR coating on each side. (b) The reflection spectra with (red solid curve) and without (blue solid curve) the AR coating. (c) Optical images of a fabricated sample under four different viewing angles. Modified with permission [113]. Copyright 2015, Wiley-VCH. (d) Schematic diagram of the 1D photonic crystal-based device for reflective CMY color generation. The device is composed of PC stacks (alternating a-Si and Si_3_N_4_ layers) with additional three layers (thin Si_3_N_4_/a-Si/thick Si_3_N_4_) on top. (e) Total absorption and separated absorption in each α-Si layer over the visible band. (f) Calculated (yellow solid curve) and measured (yellow dashed curve) reflection spectra of the proposed device. The red curves are transmission spectra of the bottom PC stacks. Inset: optical image of a fabricated sample. Scale bar: 1 cm. Reproduced with permission [114]. Copyright 2019, Tsinghua University Press.

**Photonic crystal**. Another approach for structural color generation is based on 1D photonic crystal (PC) resonators, which are composed of multiple thin films with alternating high and low index materials [111–113]. Various structural colors can be produced via properly adjusting the thickness of each layer. Lee and co-workers design a 1D PC device, which generates red color with high angular tolerance (up to ±70°) and polarization insensitivity [113]. Schematic diagram of the proposed device is shown in Figure 8(a), which is composed of alternating Si_3_N_4_ and α-Si thin films and two dielectric Si_3_N_4_ AR coatings. To generate a vivid red color (with central wavelength *λ*_c_ = 800 nm), thickness of each alternating layer inside the PC is designed to be one quarter wavelength (i.e., λ_c_/4*n*). Based on this design rule, thicknesses of the Si_3_N_4_ (*n* = 2) and α-Si (*n* = 4) layers are set as 100 nm and 50 nm, respectively. Moreover, AR coatings on both the top and bottom sides are chosen to be 50 nm thick in order to suppress reflection of incident light with wavelengths ranging from 400 nm to 600 nm (Figure 8(b)). Figure 8(c) shows the optical images of a fabricated device under four oblique viewing angles. Vivid red colors are maintained over a wide angular range.

In spite of additive colors (such as the afore-mentioned red color), subtractive colors including cyan (C), magenta (M), and yellow (Y) can also be created using the 1D PC configuration [114]. Schematic diagram of a PC-based device is shown in Figure 8(d), which is composed of PC stacks (alternating a-Si and Si_3_N_4_ materials) with additional three layers (thin Si_3_N_4_/a-Si/thick Si_3_N_4_) incorporated on top of the device. This structure can be effectively simplified into an asymmetric FP cavity by treating the bottom PC stacks as a reflective mirror for the visible band. Cyan, magenta, and yellow colors are successfully created by properly designing thicknesses of the added thin Si_3_N_4_ and a-Si layers while maintaining high near-infrared (NIR) transmission (the red curves in Figure 8(f)) without changing the bottom PC stacks. To better understand the working principle of this design, a yellow-color device is analyzed as an example. The yellow color is created by suppressing light reflection within 400 nm–500 nm while maintaining high reflection intensity for the remaining visible spectrum. The reflection suppression for wavelengths between 400 nm and 500 nm is realized through the absorption in α-Si layers, especially the 1st α-Si layer (the red solid curve in Figure 8(e)). The device’s measured (yellow dashed curve) and calculated (yellow solid curve) reflection spectra are plotted in Figure 8(f), with the image of a vivid yellow-color sample shown in the inset. Furthermore, the proposed device exhibits great angular insensitivity (up to ±60°) for both TE and TM polarizations, benefiting from the employed high refractive index materials.

### 3.2 Subwavelength nanostructure array based structural colors

Subwavelength nanostructures support different kinds of resonances and have been widely employed for generating structural colors. Their reflection and transmission spectra, as well as electric and magnetic field profiles can be computed using various techniques such as finite difference time domain (FDTD) method, finite element method (FEM), and rigorous coupled wave analysis (RCWA). In the following part, we will elaborate on several representative resonance phenomena supported by subwavelength nanostructures and their applications in creating structural colors of diverse functionalities.

**Guided mode resonance**. Guided mode resonance (GMR) can be considered as a re-distribution of light-wave energy induced by the coupling between externally propagating diffracted light and modulated waveguide leakage wave [115–119]. When diffracted light from the grating is phase matched with the leaky mode supported by the waveguide, a sharp resonant reflection or transmission peak occurs with electric and magnetic fields well confined inside the waveguide layer. GMR usually exhibits narrow linewidth and high efficiency, and has been employed for structural color generation [81], [82], [83, 120].

**Figure 9:**
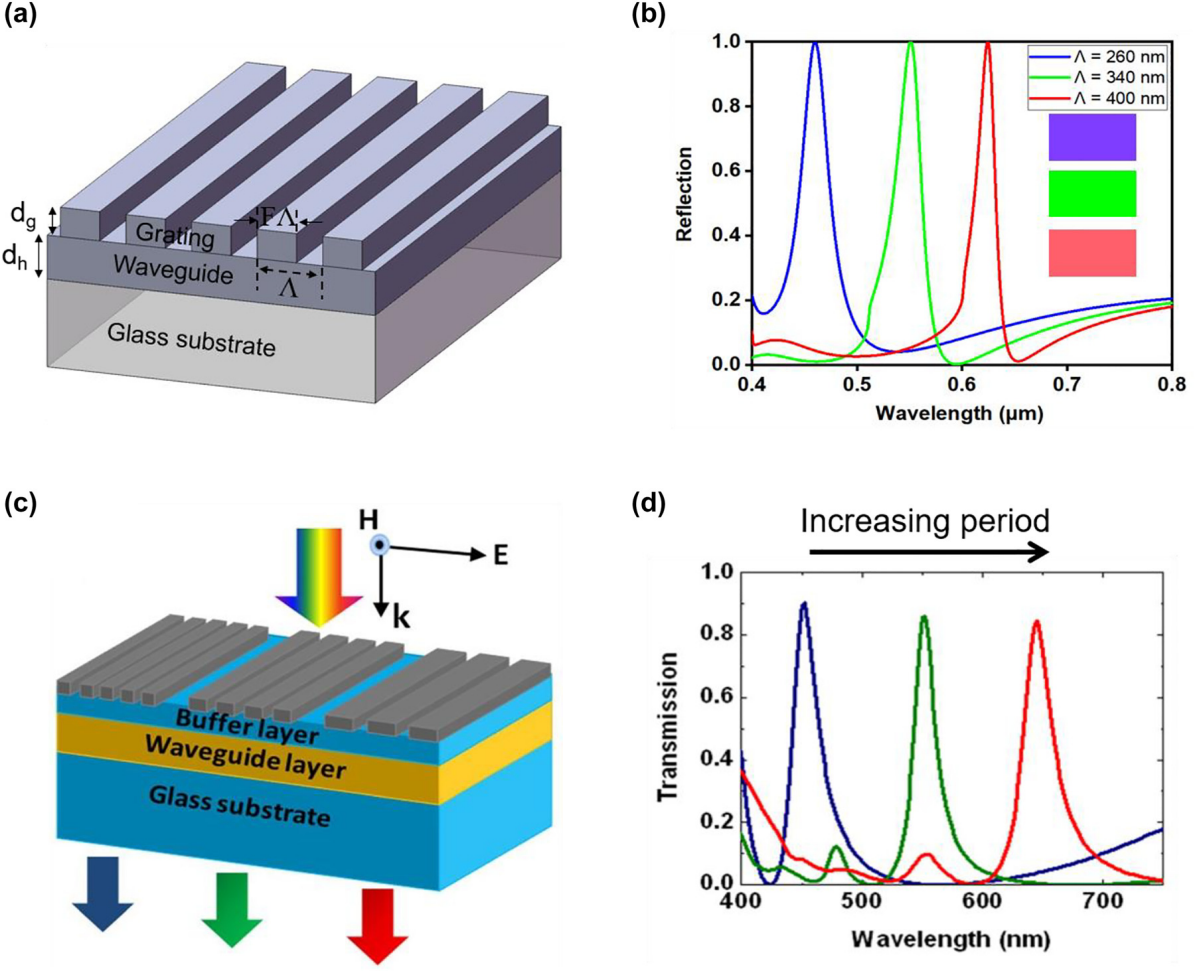
Guided mode resonance for structural color generation. (a) Schematic diagram of a representative GMR-based structural color device, consisting of a grating layer, a waveguide layer, and a substrate. *d*_g_ and *d*_h_ represent the grating depth and planar waveguide thickness, respectively. *F* denotes the grating filling ratio, defined as the ratio of its width to period. (b) Calculated reflection spectra of three GMR devices under normally-incident TM polarized light (with electric field oscillating perpendicular to the 1D grating). The device consists of TiO_2_ gratings (*d*_g_ = 80 nm) patterned over a thin TiO_2_ waveguide layer (*d*_h_ = 60 nm) on a glass substrate. The grating period (Λ) is respectively chosen as 260 nm, 340 nm, and 400 nm, while the filling ratio (*F*) is fixed as 0.5 for all three samples. Inset shows the perceived colors based on the reflectance spectra. (c) Schematic illustration of a transmission-type GMR-based structural color device, comprising an Ag grating layer, a SiO_2_ buffer layer, a Si_3_N_4_ waveguide layer, and a glass substrate. (d) Calculated transmission spectra under TM polarized normal incidence. In the calculation, the filling ratio is fixed as 0.75, and the grating periods for RGB colors are respectively chosen as 280 nm, 340 nm, and 400 nm. Thicknesses of grating layer, the buffer layer, and the waveguide layer are set as 40 nm, 50 nm, and 100 nm, respectively. Modified with permission [128]. Copyright 2011, American Institute of Physics.

Schematic diagram of a typical GMR-based structural color device is illustrated in Figure 9(a), which is composed of a grating layer, a waveguide layer, and a substrate. *d*_g_ and *d*_h_ represent depth of the grating and thickness of the planar waveguide layer, respectively. *F* denotes filling ratio of the grating, defined as the ratio of its width to period (Λ). The grating period is chosen to be smaller than the wavelength of incident beam, so that only the zeroth-order diffraction beam can propagate in free space while all higher-order diffraction beams are evanescent. In most cases, the medium on the incident side is air (with a refractive index of 1.0), and the substrate is glass (with a refractive index of ∼1.5). The waveguide layer is usually made of dielectric medium with high refractive index over the visible, such as titanium dioxide (TiO_2_) [121, 122], hafnium oxide (HfO_2_) [123, 124], silicon nitride (Si_3_N_4_) [81, 84, 125, 126], and zinc sulfide (ZnS) [127], to achieve high diffraction efficiency. Both dielectric and metallic materials can be employed to construct the subwavelength gratings. Figure 9(b) shows three reflection spectra generated from a GMR device consisting of TiO_2_ gratings patterned over a thin TiO_2_ homogeneous waveguide layer on a glass substrate. The perceived colors based on the reflectance spectra are shown in the insets, exhibiting R, G, and B colors of high purity and brightness.

An example of transmission-type GMR-based structural color design is depicted in Figure 9(c), which is composed of a subwavelength Ag grating layer, a SiO_2_ buffer layer, a Si_3_N_4_ waveguide layer, and a glass substrate [128]. The triple dielectric layer (SiO_2_ buffer layer, Si_3_N_4_ waveguide layer, and glass substrate) forms a new hybrid waveguide, and the Ag grating layer provides additional momentum for an incident light to be coupled into the waveguide layer. As the grating period increases from 280 nm to 400 nm, three representative transmission spectra respectively for RGB colors are generated under normally-incident TM polarized light (Figure 9(d)). Compared to the afore-mentioned TiO_2_-based device, this device’s peak diffraction efficiencies are less than 100% due to the absorption of Ag metallic gratings.

**Figure 10:**
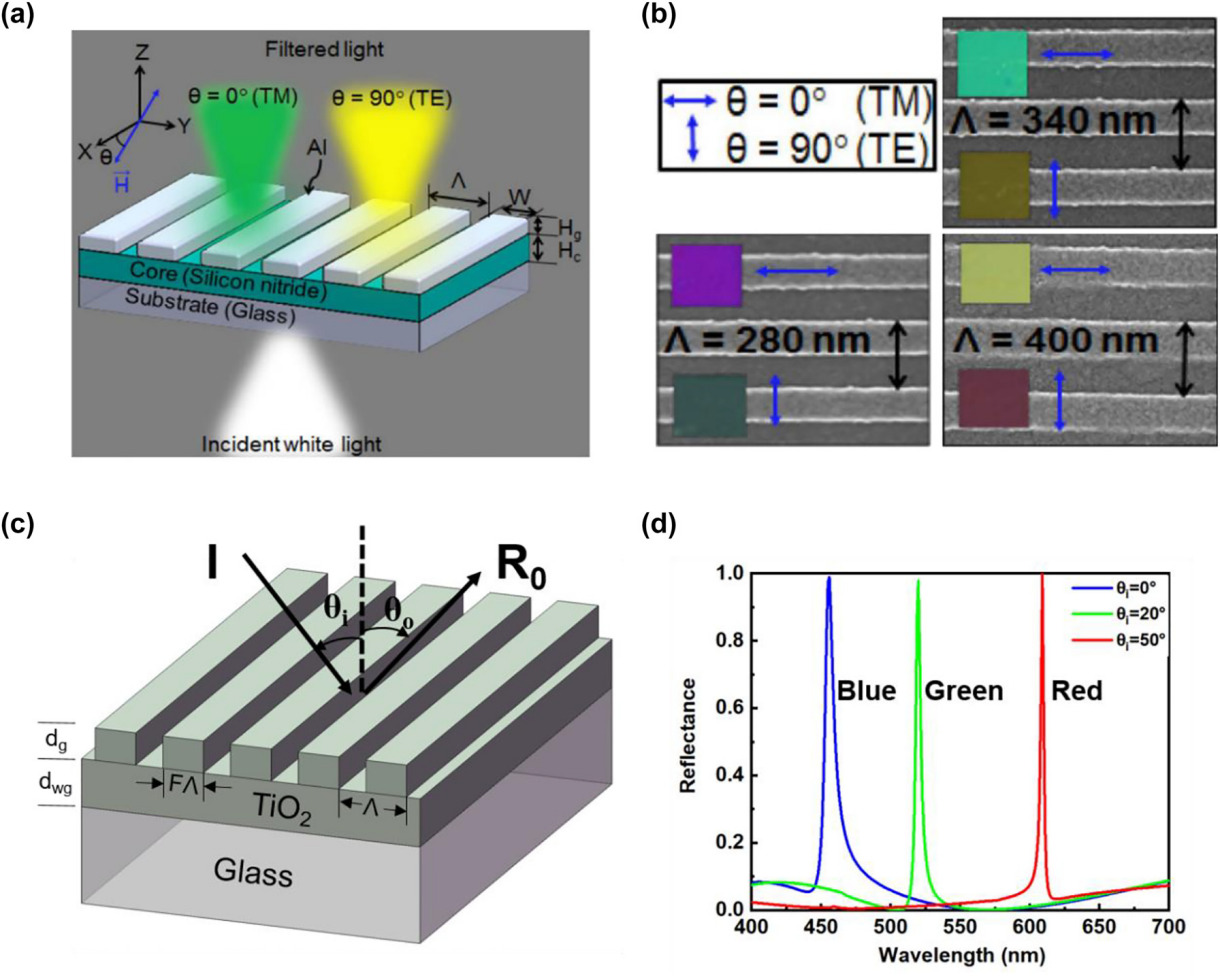
Guided mode resonance for tunable structural color generation. (a) Schematic diagram of a GMR-based nanostructure for polarization-dependent structural color generation. The device is composed of a 40 nm thick (*H*_*g*_) Al grating layer, a 100-nm thick (*H*_C_) Si_3_N_4_ waveguide layer and a glass substrate. Λ and *W* represent the period and width of the Al grating. TM polarization refers to the electrical field vector oscillating along *θ* = 0° direction, and TE polarization refers to the electrical field vector oscillating along *θ* = 90° direction. (b) SEM images of fabricated 1D-Al-grating based devices and the associated optical photos under TM- and TE-polarized illumination. Periods of the Al gratings are set as 280 nm, 340 nm, and 400 nm, respectively. The filling ratio is fixed as 0.5 for all samples. Modified with permission [129]. Copyright 2017, Nature Publishing Group. (c) Schematic diagram of a GMR-based device for angle-dependent structural color generation. The device consists of a 55 nm thick (*d*_g_) TiO_2_ grating, a 110 nm thick (*d*_wg_) TiO_2_ waveguide layer, and a glass substrate. *θ*_*i*_ and *θ*_*o*_ represent angles of the incidence and reflection, respectively. Here, refractive index of the lossless TiO_2_ is set as 2.2 over the visible range. (d) Calculated reflection spectra under TM polarized illumination (electric field vector orthogonal to the grating). Angles of incidence for red, green, and blue colors are set as *θ*_*i*_ = 50°, 20° and 0°, respectively. The TiO_2_ grating has a period of 260 nm and a filling ratio of 0.5.

For a 1D subwavelength grating based GMR device, it exhibits different resonant characteristics and reflection (or transmission) spectra for illumination light polarized along and orthogonal to its grating axis [115]. Based on this principle, switchable structural colors can be obtained by varying the state of polarization of the incident light [82, 129]. For example, a polarization-state-controlled structural color device is proposed, consisting of a 40 nm-thick (*H*_g_) 1D Al grating over a 100 nm-thick (*H*_c_) Si_3_N_4_ planar waveguide layer (Figure 10(a)) [129]. When the state of polarization of the illumination beam is switched from TM to TE, the devices exhibit different transmitted colors (Figure 10(b)).

In spite of obtaining different colors by modulating the state of polarization, tunable structural colors can also be generated through varying the angle of incidence of the illumination light [115]. This angle-dependent spectral feature can be explained in terms of the propagation constant (*β*) of the wave vector [130]:

(24) $$\beta_{m}=2\pi \left(\frac{1}{\Lambda }+m\frac{\sin\theta }{\lambda_{r}}\right)$$

where *m* denotes the diffraction order and *θ* is the angle of incidence. As *θ* increases, propagation constant corresponding to the backward-diffracted wave vector (*m* = −1) decreases while propagation constant corresponding to the forward-diffracted wave vector (*m* = +1) increases. Moreover, *λ*_*r*_ is inversely-proportional to *β*. As a result, the resonant wavelength varies with the incident angle *θ*, leading to diverse color generation. A GMR-based angle-dependent structural color device is proposed, consisting of a subwavelength grating layer (TiO_2_), a waveguide layer (TiO_2_), and a glass substrate (Figure 10(c)). Three distinct reflection curves corresponding to red, green, and blue colors are generated under TM polarized illumination (electric field vector oscillating orthogonal to the grating) from the same structure, by setting the illumination angle *θ*_*i*_ as 50°, 20°, and 0°, respectively (Figure 10(d)).

**Plasmon resonance**. Plasmon polaritons are electromagnetic oscillations bound to metal–dielectric interfaces. They can be further categorized into two types: surface plasmon polaritons (SPPs) and localized surface plasmons (LSPs), where SPPs refer to propagating polaritons that travel along metal–dielectric interfaces, and LSPs refer to electron oscillations that are bound to the surfaces of isolated metallic nanostructures [131]. By their unique properties such as subwavelength confinement and large field enhancement, plasmon polaritons have found various applications in imaging, sensing, lasing, energy harvesting, and spectrum filtering [132–141]. Researchers have explored various plasmonic structures for vivid structural color generation and we will elaborate on some representative examples in the following text.

**Figure 11:**
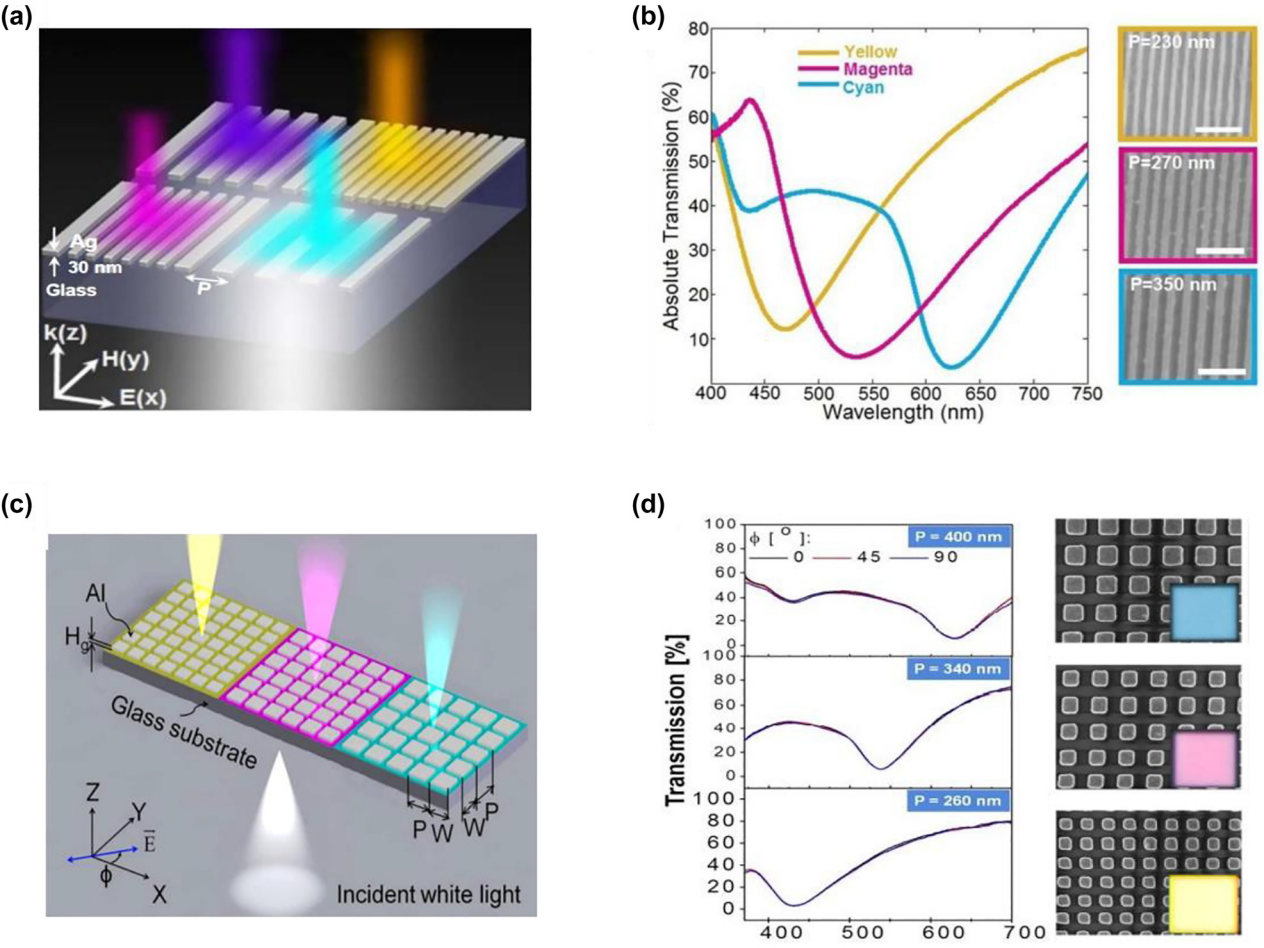
Plasmon resonance for structural color generation. (a) Schematic diagram of a 1D plasmonic nanostructure, consisting of 30 nm-thick Ag nano-gratings arrayed over a period of P on a glass substrate. (b) Left panel: experimentally measured transmission spectra under normally-incident, TM polarized light. For the three transmission colors of yellow, magenta, and cyan, the grating’s filling ratio is fixed at 0.5 while its period is set as 230 nm, 270 nm, and 350 nm, respectively. Right panel: corresponding SEM images. Scale bar: 1 μm. Reproduced with permission [142]. Copyright 2013, Nature Publishing Group. (c) Schematic diagram of a 2D plasmonic nanostructure, consisting of 40 nm thick (*H*_g_) Al nano-patches on a glass substrate. (d) Left panel: Transmission spectra of three different samples measured under illumination light of different polarization directions. Periods of the Al nano-patches are chosen as 400 nm, 340 nm, and 260 nm, respectively, and the filling ratio (W/P) is fixed as 0.5. Right panel: corresponding SEM images and generated colors (insets) of the three samples. Modified with permission [143]. Copyright 2014, American Chemical Society.

Transmission-type, subtractive colors with peak intensities of ∼70% have been realized by a plasmonic device with an ultrathin nanostructured metallic film [142]. Figure 11(a) shows the schematic drawing of the designed structure, consisting of a 30 nm-thick, 1D Ag nano-grating array on a glass substrate. The device exhibits enhanced reflection as well as absorption at its resonant wavelength when illuminated by a normally-incident, TM polarized light (electric filed along the *x*-axis), leading to a dip in the device’s transmission spectrum. Three distinct transmission colors of yellow, magenta, and cyan can be produced by fixing the grating’s filling ratio as 0.5 and setting its period respectively as 230 nm, 270 nm, and 350 nm (Figure 11(b)).

The afore-mentioned 1D metallic grating based structural color device exhibits a high polarization-sensitive response, which is undesirable for various imaging and sensing applications. To reduce sensitivity to the polarization state of illuminating light, 2D symmetric nanostructure arrays can be employed. A representative metasurface design composed of 2D arrays of aluminum (Al) nano-patches on a glass substrate is illustrated in Figure 11(c) [143]. Similar to the 1D metallic grating, different transmission colors can be obtained by varying the period of 2D symmetric nano-patches (Figure 11(d)). Moreover, the device exhibits close-to-invariant transmission spectra as polarization state of the incident light varies.

The spectral responses as well as generated colors of SPP-based plasmonic devices usually exhibit high sensitivity to the incident angle of the illumination light. This is due to the fact that different frequency components of the incident light are coupled to the SPPs as the angle of incidence varies [144]. Such angular sensitivity largely limits the device’s application in various fields such as displaying and imaging. To mitigate the above issue, structural colors that are based on LSPs have been explored.

**Figure 12:**
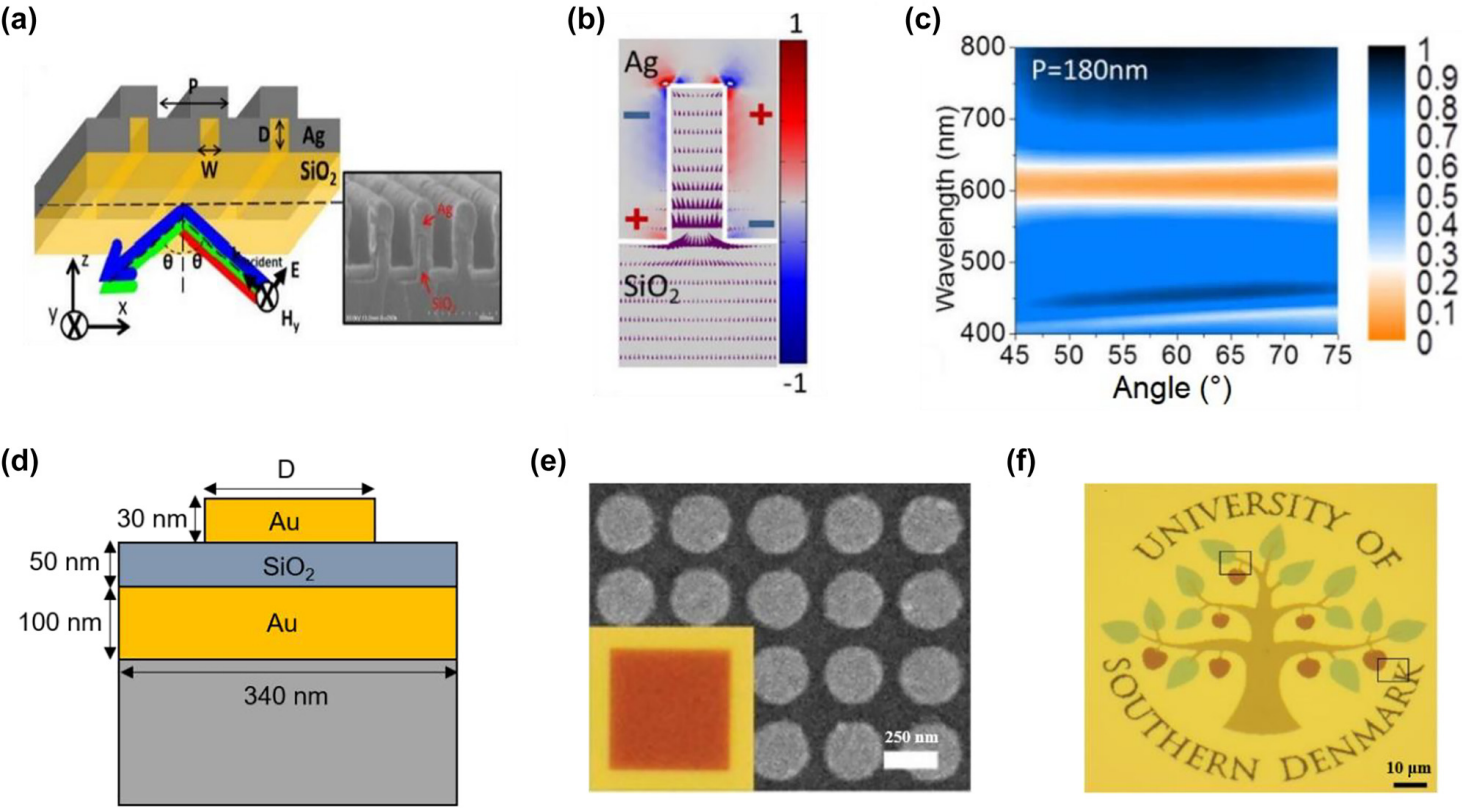
Plasmon resonance for angle-insensitive structural color generation. (a) Schematic diagram of a metallic nanoslit-based angle-insensitive structural color device, where an optically-opaque Ag layer is conformally coated on a 1D subwavelength-pitch fused silica grating layer. The associated SEM image of a fabricated device is displayed in the inset. (b) Polarization charge and Poynting vector distribution of light funneled into a nano-slit, represented by the red–blue surface plot and purple arrows, respectively. (c) The angularly-resolved reflection spectra of a device under TM polarized illumination (magnetic field oscillating along the grating direction). The period, width, and height of the grating are respectively chosen as 180 nm, 50 nm, and 130 nm. The device exhibits almost-identical subtractive colors as the angle of incidence varies. Modified with permission [145]. Copyright 2013, Nature Publishing Group. (d) Schematic illustration of a metal-insulator-metal (MIM) configuration for angle-insensitive structural color generation. The unit cell consists of a circular Au nanodisk, a continuous Au layer, and a SiO_2_ layer in between. (e) SEM image of a fabricated sample consisting of arrays of 270 nm-diameter Au nanodisks. Inset is the associated optical photo. (f) A colored logo of the University of Southern Denmark, created using the designed MIM plasmonic structures. Modified with permission [153]. Copyright 2014, American Chemical Society.

Figure 12(a) illustrates the schematic of an angle-independent plasmonic device which relies on LSPs, as well as an SEM image of a fabricated sample [145]. In this design, an optically-opaque Ag layer is conformally coated onto a subwavelength-pitch fused silica (SiO_2_) grating. Instead of relying on coupling the incident light energy into the grating-assisted SPP mode, geometric parameters of the 1D grating (width, height, and pitch) are deliberately chosen such as the incident light energy is trapped inside the isolated nano-slit and coupled to a metal–insulator–metal Fabry–Pérot (MIMFP) mode, a localized resonant mode facilitated by the light funneling effect [146–148] (Figure 12(b)). As the angle of incidence varies, the device exhibits close-to-identical subtractive colors in reflection (Figure 12(c)). It is worth noting that although this device is designed to operate only under TM polarized illumination (magnetic field oscillating along the grating direction) due to the employed 1D grating structure, both polarization- and angle-insensitive devices based on similar working principles can be realized by employing 2D subwavelength grating structures [86, 149].

Metal–insulator–metal (MIM) configurations which support gap-plasmon resonance (GPR), a representative kind of LSPs, have also been employed to realize angle-independent structural color devices [85, 150–152]. Schematic diagram of a GPR-based device is illustrated in Figure 12(d). Each unit cell consists of an Au nano-disk hovering on a continuous Au film with a thin SiO_2_ film sandwiched between them [153]. A subwavelength period of 340 nm is chosen to minimize high-order diffraction from the structure under normal incidence. Different GPRs of resonant frequencies over the whole visible range can be realized by tuning diameter of the Au nano-disk as well as thickness of the SiO_2_ dielectric layer. The SEM image of a fabricated sample consisting of 270 nm-diameter Au nano-disk arrays is displayed in Figure 12(e), showing high-fidelity circular disk patterns. The inset is an associated optical photo, exhibiting a homogeneous generated color. Using the designed plasmonic structures, a high-quality and fine-resolution colored University of Southern Denmark logo is successfully fabricated (Figure 12(f)).

**Mie resonance**. Mie scattering theory can describe the light scattering behavior of a particle with size similar to or larger than the wavelength of incident light [154–156]. It applies to scattering objects with various shapes and different constituent materials (such as dielectrics, metals, as well as semiconductors). The theory also accommodates cases of incident light with a wide range of frequencies. Over recent years, all-dielectric Mie resonators have been exploited for generating structural colors with high spatial resolution, low optical loss, and robust angular insensitivity [25, 88, 89].

**Figure 13:**
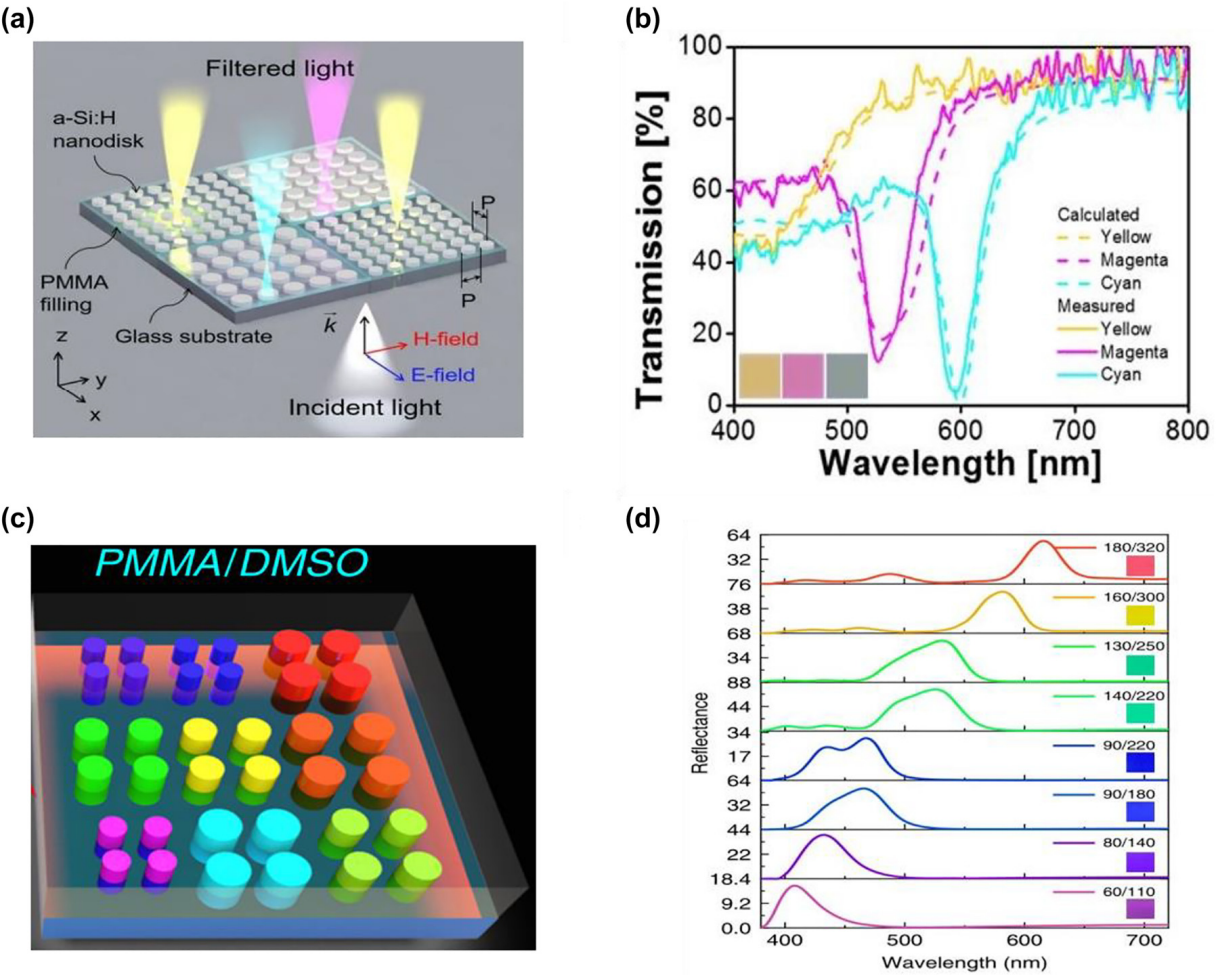
Si based Mie resonance for structural color generation. (a) Schematic illustration of Mie resonators for transmission-type structural color generation, where each unit consists of a 2D α-Si:H nanodisk arrayed on a glass substrate. The nanodisks are embeded inside PMMA. (b) Calculated (dashed curves) and measured (solid curves) transmission spectra of yellow, magenta, and cyan colors. Insets: photos of the generated colors. The height of the nanodisk is fixed as 80 nm, and its period (diameter) is chosen as 150 nm (80 nm), 330 nm (130 nm) and 379 nm (170 nm) to generate the yellow, magenta, and cyan color, respectively. Reproduced with permission [165]. Copyright 2017, Nature Publishing Group. (c) Schematic illustration of Mie resonators for reflection-type structural color generation. The device is composed of arrays of Si nanodisks on a sapphire substrate, over-coated with a refractive index matching layer (PMMA or DMSO). (d) Experimentally measured reflection spectra of the 100-nm thick Si metasurfaces with different lattice sizes (Diameter (R), Period (*l*)). Insets: recorded colors under bright-field microscope. Modified with permission [166]. Copyright 2020, Nature Publishing Group.

Both individual nanosphere and nanowire made of Si have been demonstrated for displaying distinct colors [157–159]. However, in many cases, the generated colors can only be observed under dark-field illumination due to the low scattering efficiency of an individual Mie resonator. To enhance the scattering efficiency, many researchers have explored devices made of arrays of Mie resonators [160–164]. For example, Park and co-workers develop transmission-type structural colors based on periodically arranged Si resonators [165]. Figure 13(a) illustrates the schematic of the proposed device, which is composed of arrays of α-Si:H nanodisks on a glass substrate. Polymethyl methacrylate (PMMA), which has a refractive index around 1.49 in the visible and serves as an index-matching layer, is employed to fill in the gap between adjacent Si nanodisks. The incident light can be filtered into distinct colors based on the electric dipole (ED) and magnetic dipole (MD) resonances supported within α-Si:H nanodisks via Mie scattering. Figure 13(b) shows the spectra of three different transmission-type colors (yellow, magenta, and cyan). The nanodisk height for all three colors is fixed as 80 nm, while the lattice spacing and the diameter respectively changes from 150 nm to 370 nm and from 80 nm to 170 nm.

In addition to transmission-type colors, Si-based Mie resonators can also produce reflection-type structural colors. For instance, Yang and co-workers demonstrate reflective RGB colors by exploiting Si-based resonators [166]. Schematic diagram of the proposed device is shown in Figure 13(c), which consists of periodic arrays of Si nanodisks on a sapphire substrate and a refractive index matching layer (PMMA or DMSO (dimethyl sulfoxide)). Both the resonant bandwidth and off-resonance intensity of the reflection spectrum are sufficiently suppressed due to the reduced refractive index contrast between the sapphire substrate and refractive index matching layer. Consequently, structural colors with high purity and large gamut are generated. Meanwhile, the intrinsic high refractive index of Si facilitates a diffraction-limit spatial resolution of the generated colors. Figure 13(d) shows the measured reflection spectra of an array of samples and the associated microscope images. Various structural colors ranging from red to blue are generated by fixing the height of Si nanodisk as 100 nm, and adjusting its diameter (lattice spacing) from 180 nm (320 nm) to 60 nm (110 nm).

**Figure 14:**
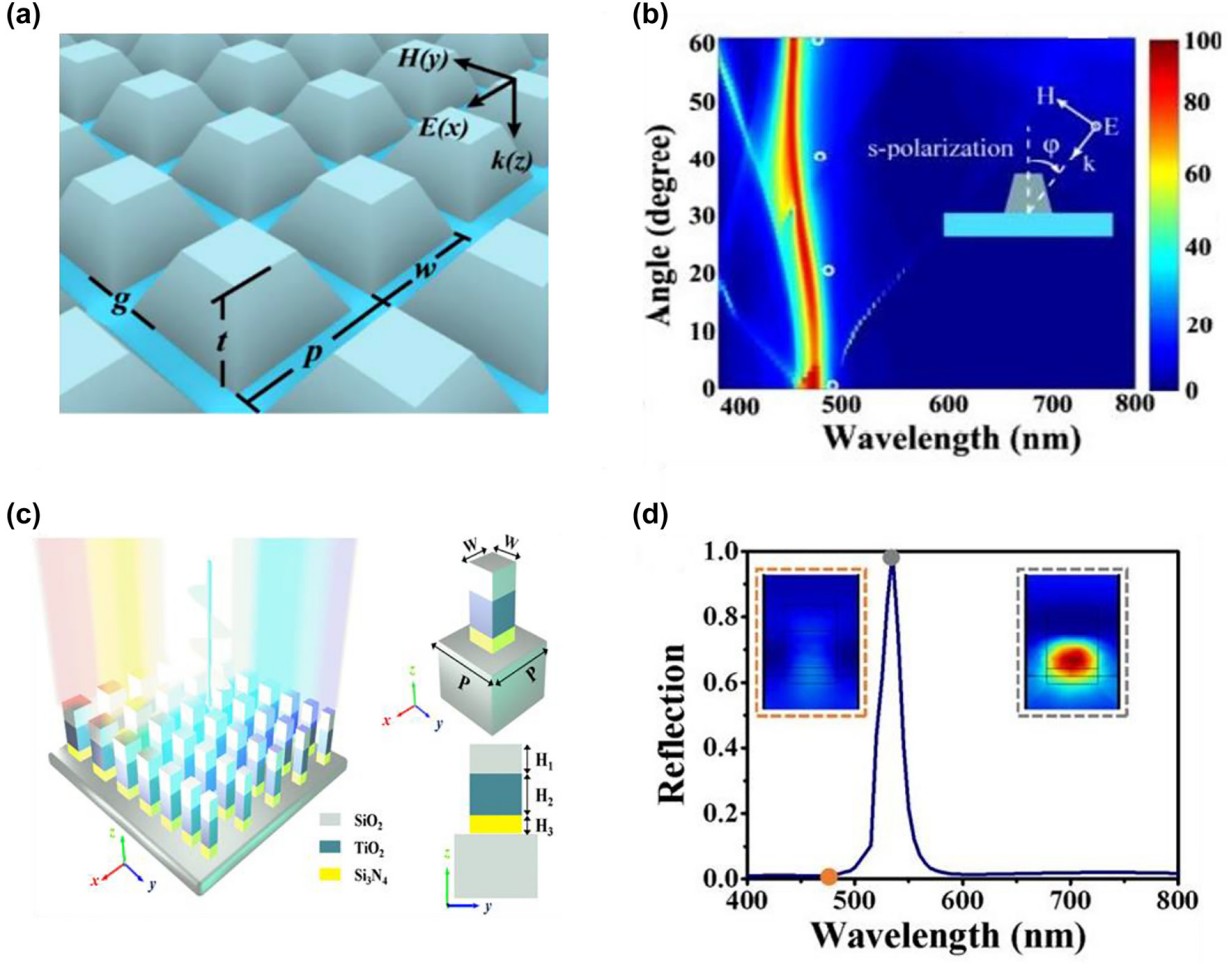
TiO_2_ based Mie resonance for structural color generation. (a) Schematic diagram of the TiO_2_-based metasurface, consisting of arrays of TiO_2_ blocks on a glass substrate. Here, p, w, g and *t* represent the period, the bottom square width, the gap between the adjacent units, and the block height, respectively. Cross-section of each block is a trapezoid with the trapezoidal corner around 72°. (b) Angularly-resolved reflection spectra under s-polarized illumination. Inset shows the angle and polarization direction of the incident light. Reproduced with permission [168]. Copyright 2017, American Chemical Society. (c) Schematic illustration of the multi-dielectric-stack metasurface, where each nanopillar is made of a vertically stacked 100 nm-thick (*H*_1_) SiO_2_ capping layer, a 140 nm-thick (*H*_2_) TiO_2_ spacer layer, and a 60 nm-thick (*H*_3_) Si_3_N_4_ bottom layer. (d) Simulated reflection spectrum of an array of stacked-layer nanoblocks, which have a period of 350 nm and width of 200 nm. Insets show the magnetic field distribution (|*H*|) at 480 nm (orange dashed box) and 535 nm (gray dashed box). Reproduced with permission [167]. Copyright 2019, American Chemical Society.

Si-based Mie resonators, however, suffer from severe absorption loss in the visible band (especially in the blue region), leading to generated colors with limited efficiency and broadened bandwidth. To mitigate such limitation, alternative dielectric materials with both high refractive index and low absorption loss over the visible, such as TiO_2_, Si_3_N_4_, and ZnS, have been exploited for creating high-performance structural color devices [167–170]. Among various materials, TiO_2_ has been widely accepted as a good candidate by its high refractive index and zero absorption loss over the visible. For example, Sun and co-workers demonstrate distinct colors by utilizing TiO_2_-based resonators [168]. Figure 14(a) shows the schematic diagram of the proposed device composed of arrays of TiO_2_ blocks on an ITO (15 nm in thickness) coated glass substrate, where the cross-section of each block is a trapezoid with the trapezoidal corner around 72°. The periodic arrays of TiO_2_ blocks generate distinct structural colors via both ED and MD resonances. Meanwhile, the proposed device exhibits a weak angle-dependent feature (Figure 14(b)). The peak wavelengths of the reflection spectra are maintained within 450–470 nm as the incident angle increases from 0° to 60°.

Yang and co-workers propose another design that can realize structural colors with high purity based on resonators having a multi-dielectric-stack configuration [167]. Figure 14(c) illustrates the schematic diagram of the proposed device, which is composed of stacked-layer nanoblocks arrayed over a silica substrate. Each nanoblock is made of a vertically stacked 100 nm-thick (H_1_) SiO_2_ capping layer, a 140 nm-thick (H_2_) TiO_2_ spacer layer, and a 60 nm-thick (H_3_) Si_3_N_4_ bottom layer. In contrast to the all-TiO_2_ structure, this stacked-layer structure exhibits reflection spectrum with suppressed high order resonance and therefore, generates colors with improved saturation. Such improvement is due to the fact that the SiO_2_ layer and the Si_3_N_4_ layer, acting as index matching layers, suppress the excitation of multipolar modes at shorter wavelengths. Figure 14(d) shows the simulated reflection spectrum of the proposed device, exhibiting only one resonant peak at 535 nm. The magnetic field distribution (|*H*|) at 480 nm shown in the inset (orange dashed box) further illustrates the suppressed resonance at shorter wavelengths.

## 4 Design methods

The design task of nanostructures for structural color generation is often high-dimensional. It could involve many degrees of freedom, including but not limited to the geometry and arrangement of meta-atoms, selection of constituent materials, number of layers, state of illumination light (angle of incidence, state of polarization), etc. In addition, different spectra could lead to the same perceived color by human eyes, a phenomenon called color metamerism [171]. Such ambiguous “many-to-one” relationship between a series of design parameters and the finally generated color makes the high-dimensional color design task even more challenging. Thus, manually designing nanostructures for color generation could be time-consuming and might end up with sub-optimal device structures. To accelerate the device design process, intelligent inverse design algorithms that can automatically find structures corresponding to specific color requirements have been applied to the design tasks of various types of structural-color-generating devices [95, 172], [173], [174], [175], [176], [177]. These inverse design methods can be categorized into two types: optimization-based approach and machine learning-based approach.

**Figure 15:**
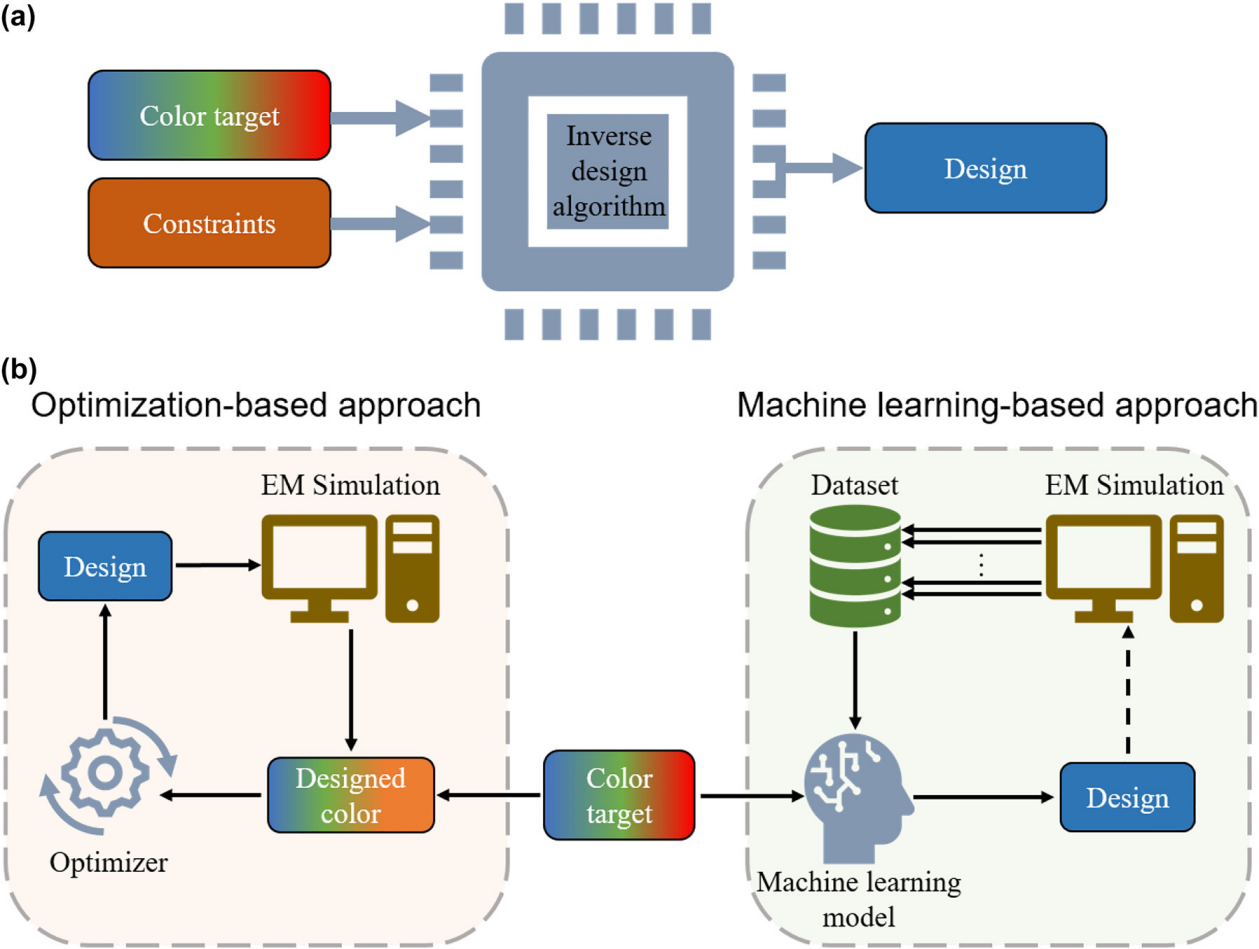
Inverse design paradigms of structural color generating metasurfaces. (a) An inverse design algorithm takes in the color target and relevant design constraints as the input to generate the design. (b) Optimization-based inverse design approaches (left panel) involve iterative evaluation of designs through EM simulation and continuous update of intermediate designs until a pre-set target is met. Machine-learning based approaches (right panel) require the designer to first synthesize a dataset containing pairs of designs and color labels through EM simulation. A machine learning model is later trained based on the synthesized dataset to predict the optimized design. After the model has been trained, machine learning-based inverse design approaches can directly predict optimized designs corresponding to the desired color target without the time-consuming iterative evaluation of various intermediate designs required by the optimization-based approaches. When reinforcement learning—a family of machine learning algorithm—is used, additional designs predicted by the model will be simulated. Both the designs and their simulated color responses will be added to the dataset to expand the explored design space, which can facilitate the learning of a more accurate model (indicated by the dotted arrow).

During an inverse design process, the algorithm takes the targeted color and relevant design constraints as inputs, and generates corresponding device designs (Figure 15(a)). Optimization-based approaches rely on iterative evaluation of designs through EM simulation and continuous update of intermediate designs until a pre-set target is fulfilled (Figure 15(b), left panel). In contrast, machine learning-based approaches first train models based on a large training dataset, and then directly predict the optimized design for a given color target without relying on any iterative EM simulation (Figure 15(b), right panel). An exception is reinforcement learning, a family of machine learning algorithms. During the learning process, it will iteratively collect data to improve its design accuracy, which can be considered a combination of optimization and machine learning.

This part will give a detailed comparison between the two different approaches based on case studies from previous structural color works. In addition, we will provide recommendations on how researchers should select structural color inverse design methods to best suit their research needs. For generic photonic inverse design methods, we refer readers to these comprehensive reviews [178–181].

### 4.1 Optimization-based approaches

Particle swarm optimization (PSO) and genetic algorithm (GA) are two most widely used optimization algorithms for the design of structural color devices. Both methods aim to minimize the difference between the targeted color and the color generated by the designed nanostructure. We will elaborate on these two methods in the following part.

**Particle swarm optimization**. Particle swarm optimization (PSO), as one representative bio-inspired algorithm, has been employed for designing metasurfaces of complicated configurations [172, 182]. In order to identify a globally optimized design that adjusts the merit function to an appropriate value, PSO first creates a set of particles with randomly-distributed initial positions. The coordinates of each particle’s position correspond to a specific group of design parameters. During the optimization process, each particle’s position is updated with a velocity determined by the position the particle itself has encountered and the best position found by all particles. The quality of each position is then evaluated by EM simulation. After sufficient rounds of iteration, the positions of all particles will converge to the same value, and an optimized design is obtained.

**Figure 16:**
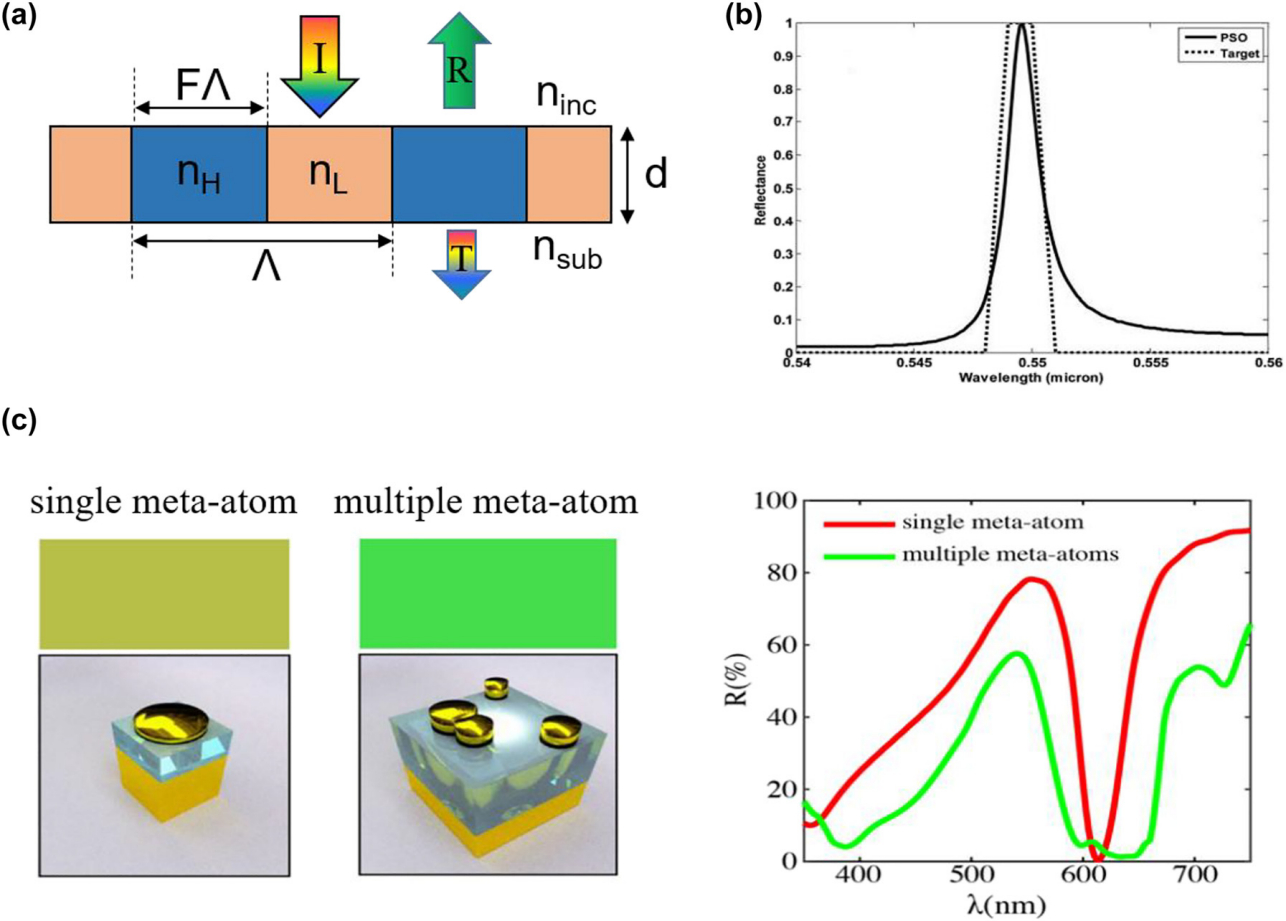
Optimization-based approaches for structural color device design. (a) Schematic diagram of a guided mode resonance grating structure. Three optimization parameters used in the PSO process are the grating thickness (*d*), period (Λ), and filling ratio (*F*). (b) Target reflection spectrum (dotted curve) and calculated reflection spectrum (solid curve) based on the optimized device parameters through PSO. Modified with permission [182]. Copyright 2007, Optica Publishing Group. (c) GA-based optimization for structural color generation. Optimizing the design parameters of the multiple-meta-atom metasurface leads to a better purity for green color compared to the single-meta-atom metasurface. Modified with permission [185]. Copyright 2020, American Chemical Society.

PSO has been successfully employed in the design task of guided mode resonance (GMR) based structural color metasurfaces [182]. For the GMR 1D grating structure shown in Figure 16(a), its thickness (*d*), grating period (Λ), and filling ratio (*F*) are the three design parameters used in the PSO process. During the optimization process, each particle’s position (described with the aforementioned parameters, *d*, Λ, and *F*) is updated according to a fitness function defined as a root mean square error function between the target reflection spectrum and the designed counterpart by PSO. After 6000 rounds of iteration, an optimized set of structure parameters (*d* = 335.9 nm, Λ = 279.1 nm, *F* = 783.0 nm) is obtained, which generates reflection spectrum (solid curve in Figure 16(b)) in good correspondence to the target one (dotted curve in Figure 16(b)).

**Genetic algorithm**. Similar to PSO, genetic algorithm (GA) is also a bio-inspired optimization algorithm and maintains a group of intermediate designs throughout its optimization process [183, 184]. Instead of mimicking the swarming behavior of flocks or fish, GA models the natural selection procedure based on carefully designed selection, crossover, and mutation rules. In each round of iteration, GA first computes the fitness scores of a set of solutions. Solutions with higher fitness scores will be selected with a higher probability to proceed to the next crossover step. During crossover, selected solutions from the previous step are randomly paired to generate offspring solutions with a small probability of mutation, where some design parameters are randomly changed by chance. Such iterative selection–crossover–mutation process will repeat until a convergence criterion is met.

Liu and co-workers employ GA for designing multiplexed metasurfaces, whose unit cell consists of multiple meta-atoms [185]. For a conventional metasurface device, its unit cell is usually composed of only one meta-atom. This makes the design relatively simple and straightforward, but at the same time, limits the device performance for certain structural color applications (Figure 16(c)). Although extending metasurface design from single meta-atom to multiple meta-atoms could potentially improve the device performance, the substantially increased number of parameters makes traditional design methodology relying on physical intuition and parameter sweeping impractical. With the aid of GA optimization, the researchers successfully obtain multiplexed metasurface designs that can generate high-purity red, green, and yellow colors. These multiplexed metasurfaces all contain four metal disks in each unit cell. During the GA optimization process, the unit cell period (*T*), height (*H*) and diameter (*D*) of the disk, and thickness (*T*) of the dielectric layer underneath the metal disks are optimized to minimize the Euclidean distance between the designed color and the color target in the CIE diagram.

GA can also be employed for multi-objective optimization, where several potentially conflicting merit functions are being maximized at the same time [173, 184]. Wiecha and co-workers apply non-dominated sorting genetic algorithm II (NSGA-II), a widely used multi-objective GA method, for designing dielectric metasurfaces with polarization-multiplexed resonances [173]. The antenna structure consists of unit cells with four rectangular Si blocks. By optimizing the design parameters including the position A, length L, and width W of each Si block, the authors aim to maximize the antenna scattering efficiency for perpendicular polarization states at two different wavelengths *λ*_*x*_ = 550 nm and *λ*_*y*_ = 450 nm, which are conflicting design goals. Starting with a set of random designs, NSGA-II successfully identifies a design that maximizes the overall scattering efficiency for both polarization states.

### 4.2 Machine learning-based approaches

Machine learning-based methods for structural color design can be grouped into model-based optimization (MBO), generative modeling (GM), and reinforcement learning (RL). MBO first trains a forward surrogate model that can efficiently predict the color of a certain design. Then, EM simulations are replaced by the trained surrogate model to speed up the structural color design process. Unlike MBO, GM directly learns a conditional distribution of design parameters given a color target. A diverse set of designs can be sampled from the learned conditional distribution. Most MBO and GM methods rely on static datasets, which may not lead to a satisfying design performance when the color target differs too much from the training data distribution. RL methods overcome this limitation by actively exploring the design space through a user-defined reward function that measures the quality of various designs.

**Model-based optimization**. In model-based optimization, a surrogate forward model that can predict the color based on the design parameters is first trained on a dataset containing an array of designs and associated color labels. Afterwards, a search process or another machine learning model is used for querying the surrogate forward model to find the optimal set of design parameters.

**Figure 17:**
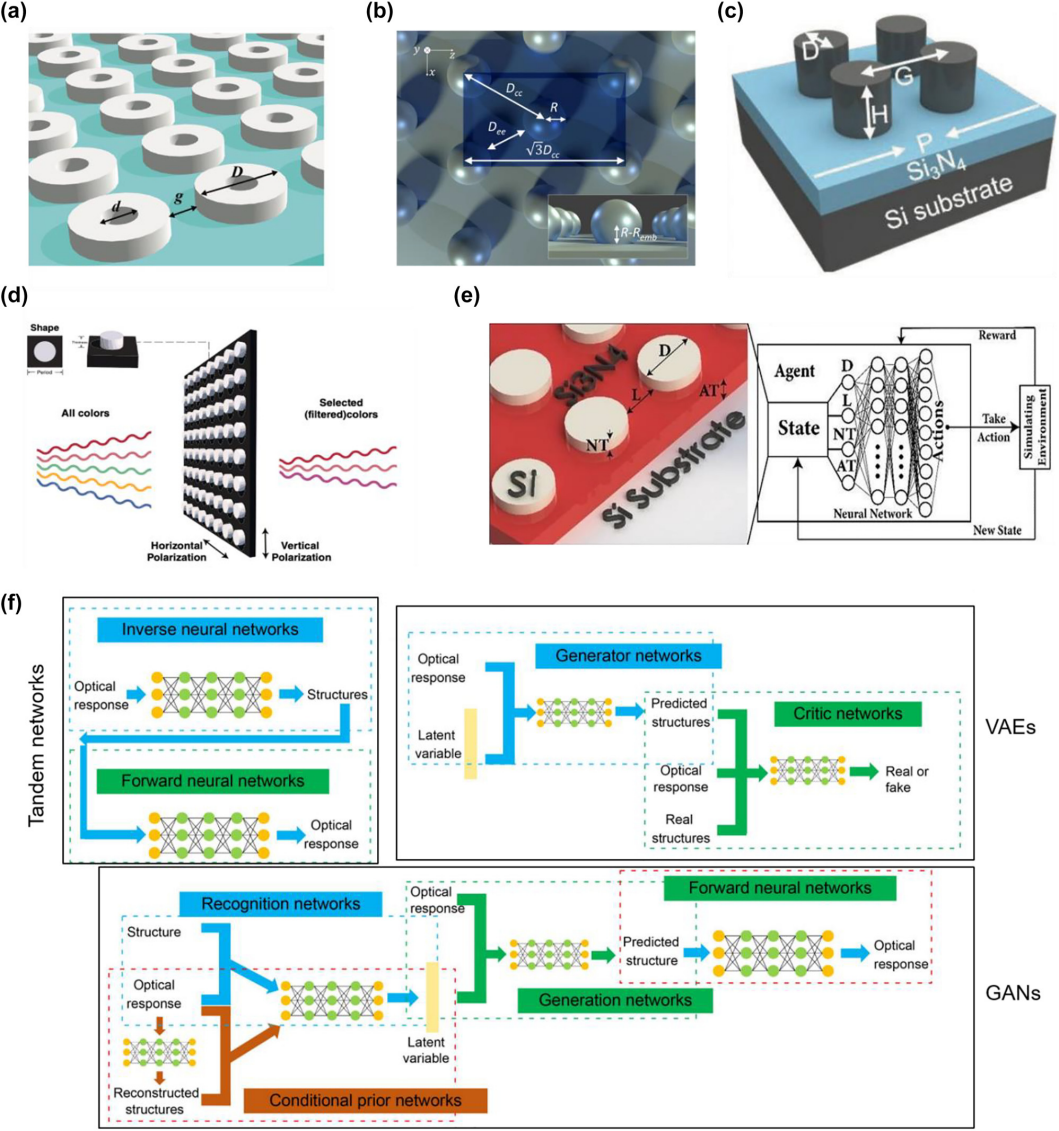
Machine learning-based approaches for structural color design. (a) Dielectric ring structure for structural color generation. The inner diameter *d*, outer diameter *D*, and the inter-ring gap g are predicted for a given color target. Reproduced with permission [186]. Copyright 2019, Royal Society of Chemistry. (b) Laser-induced nanoparticles for structural color generation. The optimal laser machining parameters are optimized with a deep neural network model. Reproduced with permission [175]. Copyright 2019, Nature Publishing Group. (c) Silicon nanorod structure designed by tandem networks. The design parameters are the diameter *D*, height *H*, gap *G*, and period *P*. Modified with permission [174]. Copyright 2019, Wiley-VCH. (d) Free-form silicon metasurface structure designed by GANs. Modified with permission [191]. Copyright 2021, Wiley. (e) Silicon nanorod structure designed by the reinforcement learning algorithm. Reproduced with permission [195]. Copyright 2019, Optica Publishing Group. (f) Neural network architectures for tandem networks (top left), Variational autoencoders (VAEs, top right), and generative adversarial networks (GANs, bottom). Modified with permission [192]. Copyright 2022, China Science Publishing and Media (CSPM) LTD.

Huang and co-workers train kernel ridge regression models to predict the CIE xy coordinates for dielectric ring arrays (Figure 17(a)) and dielectric pyramid arrays with different design parameters [186]. The trained models are later used as the surrogate models to replace the computationally-expensive EM simulation. With the surrogate models, the authors search the optimal design parameters that can lead to the target design with a simple greedy search process, which iteratively updates each design parameter and evaluates the design’s performance based on the surrogate models. Deep neural networks have also been trained as surrogate models to predict the structural color given device parameters during fabrication. Baxter and co-workers train neural networks to predict the RGB values of plasmonic nanoparticles (Figure 17(b)) based on laser machining parameters [175]. Similarly, a greedy search process is later applied to find the laser machining parameters that can produce the desired color.

Instead of relying on searching the input space of a trained forward model, Gao and co-workers apply a tandem network that combines a forward model and an inverse model to predict the metasurface designs (Figure 17(c)) for user-specified CIE xyY coordinates [174]. Specifically, the forward model and the inverse model are trained in two separate steps. After the forward model is trained, its parameters would be fixed when training the inverse model. The tandem network has recently been improved by using the L^*^a^*^b^*^ target to allow more uniform color design accuracy [95], and introducing a mean squared error regularizer to avoid outputting infeasible designs [176]. Roberts and co-workers augment the labeled data with unlabeled synthetic data containing only the CIE *xy* coordinates to improve the inverse design accuracy [187]. In addition to patterned metasurfaces, tandems networks have also been used for the inverse structural color designs based on nanoparticles [188].

**Generative models**. Variational autoencoders (VAEs) [189] and generative adversarial networks (GANs) [190] are both popular generative modeling methods. They directly learn the conditional distribution of design parameters for the target color. A diverse set of solutions can be sampled from the learned conditional distribution. Han and co-workers train GANs for designing transmissive metasurface color filters based on free-form meta-atoms (Figure 17(d)) [191]. Their results show that GANs can output free-form metasurface designs for a wide range of possible transmission spectra within a few seconds. Ma and co-workers compare the performance of both VAEs and GANs against the tandem network, an MBO method, on designing nanopillar structures and free-form structures all based on Si [192]. Neural network architectures of all three models are shown in Figure 17(f). The authors find that generative models including VAEs and GANs outperform tandem networks in terms of design diversity, which helps users to select designs that are more amenable to fabrication process. In addition, the authors show that the design accuracy of generative models can be improved by sampling multiple designs and verifying the sampled designs through EM simulation. Ren and co-workers also present similar findings in their recent work comparing the performance of various deep learning models for artificial electromagnetic material inverse design [193].

**Reinforcement learning**. Most model-based optimization and generative modeling methods are trained with static datasets, which may end up with sub-optimal design accuracy when the color target deviates too much from the training data distribution. Reinforcement learning (RL) addresses this issue by actively collecting additional data through a trial-and-error learning process [194]. Similar to optimization-based approaches, RL also requires an iterative color property evaluation through the design process. However, RL improves upon conventional optimization-based approaches by learning a mapping between the color target and the optimal design parameters through intelligently exploring. As more data are explored through the learning process, RL can gradually acquire a more efficient strategy for designing a wide variety of colors. Sajedian and co-workers apply RL to design silicon metasurfaces that can generate targeted CIE xyz coordinates (Figure 17(e)) [195]. Wang and co-workers develop an RL approach that allows designing multilayer thin-film based structures with a variable number of constituent layers [196]. The developed method has been used for designing a 14-layer broadband absorber exhibiting a close-to-perfect black color.

### 4.3 Comparison between optimization and machine learning based approaches

Both optimization and machine learning approaches have advantages and disadvantages when applied to the structural color design task. The significant difference between optimization-based approaches and machine learning-based approaches is whether a model is learned through the design process. Learning a model allows one to efficiently predict the design for an unseen color target, while optimization-based approaches always require a time-consuming iterative search process for every new design target. However, collecting a large dataset for training accurate machine learning models is not trivial. If the researchers expect to only solve for a handful of designs, optimization-based inverse design approaches could be more practical. When many designs are needed (e.g., finding structures for reconstructing all color pixels in a high-resolution image), synthesizing datasets for training machine learning models becomes worthwhile because the dataset generation process is a one-time cost. As more and more new design targets are encountered, machine learning-based approaches can save significantly more time than optimization-based approaches.

**Figure 18:**
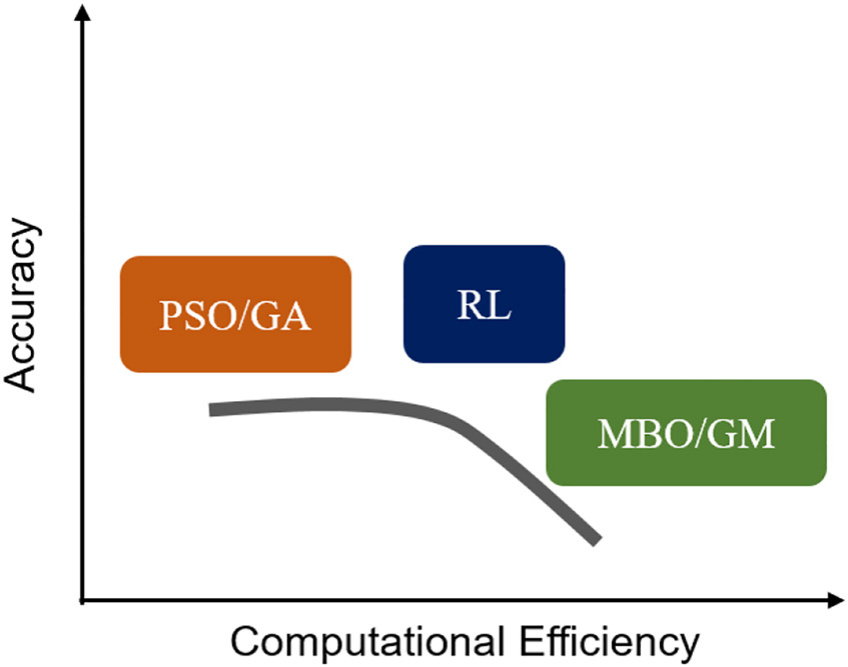
Comparison between different inverse design methods for structural color generation in terms of accuracy and computational efficiency.

In addition to efficiency, researchers should also consider the design accuracy of different inverse design methods (Figure 18). Because optimization-based approaches and RL involve iterative dataset collection that allows an active design space exploration, they are often more accurate than static model-based methods. Additionally, advanced RL methods could perform better than conventional optimization-based approaches [196]. However, both approaches are slow due to the requirement of iterative EM simulation. Thus, if researchers value efficiency more than accuracy, MBO or GMs should be used for the design process, while PSO, GA, and RL are more suitable for obtaining highly accurate designs for a small set of color targets. Moreover, we believe that combining optimization and machine learning to form a hybrid method may bring the best of both worlds. Wang and co-workers recently propose a method that combines a neural network model and PSO for efficient structural color design [197]. The proposed method can provide designs for an array of multilayer thin films to reconstruct images with more than 200,000 pixels within few hours.

## 5 Fabrication techniques

### 5.1 Multi-layer thin film based structural colors

Structural colors based on multilayer thin films are commonly fabricated using an array of vacuum-based thin film deposition methods, including physical vapor deposition (e.g., thermal/electron-beam evaporation, magnetron sputtering, etc.) [198–203], chemical vapor deposition (e.g., low pressure chemical vapor deposition-LPCVD, plasma enhanced chemical vapor deposition-PECVD, etc.) [204–206], and atomic layer deposition (ALD) [207–209]. To further reduce the fabrication cost and avoid the need for vacuum-based systems, alternative thin film deposition methods have been explored. Examples include electrochemical deposition and self-assembly method. Moreover, to realize monolithic color integration, planar thin film structures with different constituent layer thicknesses need to be fabricated over the same substrate. Towards this goal, various lithography techniques have been employed together with the afore-mentioned thin film deposition techniques.

**Electrochemical deposition**. Electrochemical deposition (electrodeposition) is a process of coating conductive or semi-conductive materials onto substrates using an electric field and redox reaction [210–212]. The employed electrochemical cells generally consist of a pair of electrodes and electrolyte solution in between. When a voltage is applied on the electrodes and an induced current passes through the electrolyte solution, metal ions in the electrolyte solution become solid metals and subsequently, get deposited on the cathode surface. Electrochemical deposition can be performed under mild conditions and allows precise growth of layers with nanometer thicknesses onto surfaces with arbitrary shapes.

**Figure 19:**
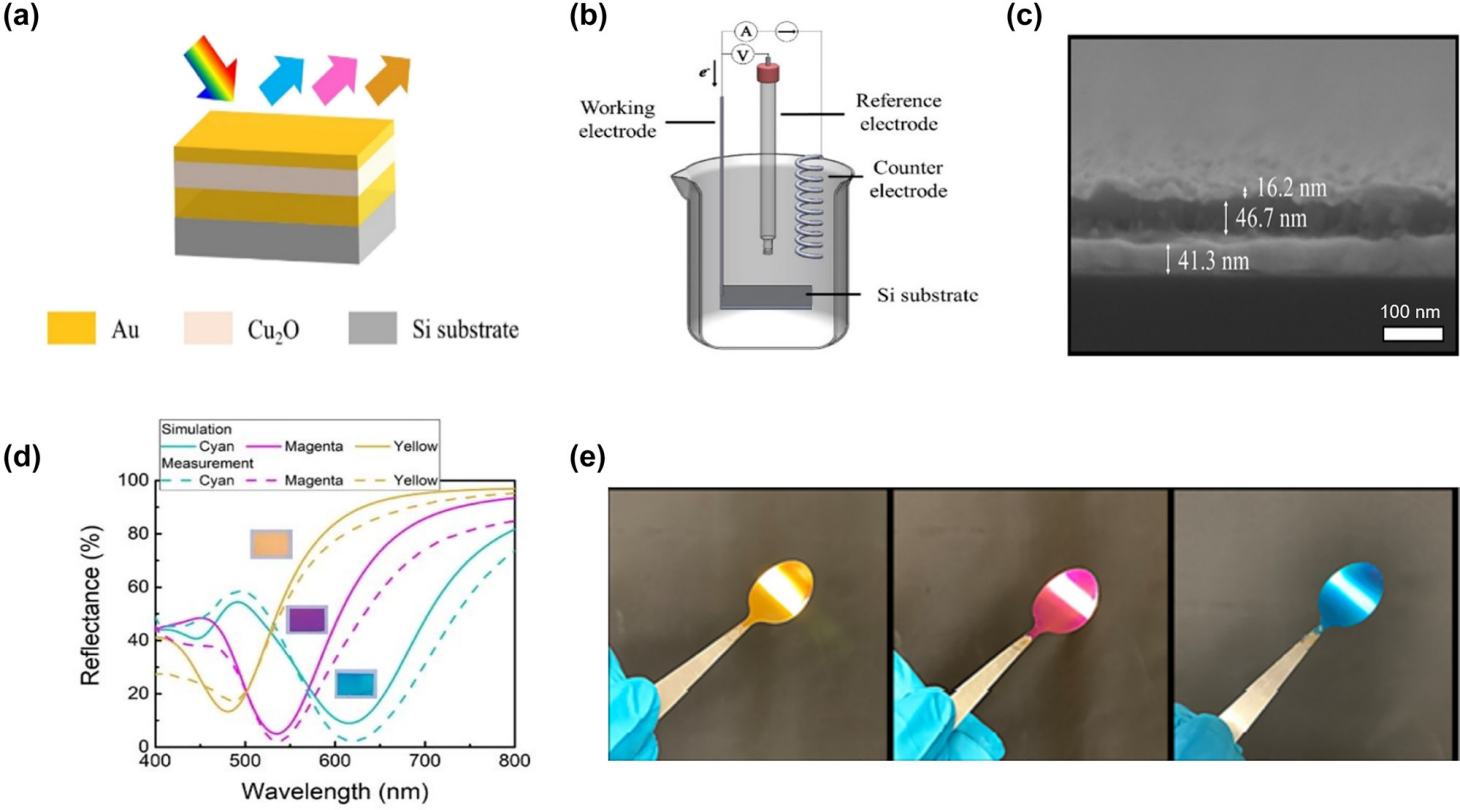
Electrochemical deposition for fabricating multi-layer thin film based structural colors. (a) Schematic diagram of the FP cavity with an Au-Cu_2_O-Au configuration over a heavily n-doped crystalline Si substrate. (b) A standard three-electrode electrochemical cell setup, consisting of a reference electrode, a working electrode, and a counter electrode. (c) Cross-sectional SEM image of a fabricated sample, showing the thickness of each electrodeposited layer. (d) Simulated (solid curves) and measured (dashed curves) reflection spectra from the cyan-, magenta-, and yellow-color samples. Insets are the associated optical images (∼1.5 cm × 1.0 cm). (e) Photos of the colored stainless-steel spoons with the electrodeposited multilayer thin films. Reproduced with permission [213]. Copyright 2019, American Chemical Society.

Ji and co-workers report a simple and low-cost electrodeposition procedure under ambient condition (*T* < 50°) for fabricating metal–dielectric–metal (MDM) FP-type structural colors [213]. The device is composed of two Au films separated by a Cu_2_O layer, and is coated on a heavily-doped n-type crystalline Si substrate (Figure 19(a)). The employed electrochemical cell (Figure 19(b)) has three electrodes, which are the reference electrode, the working electrode (n-type Si substrate), and the counter electrode (Pt mesh). A cross-sectional SEM image of one fabricated sample is displayed in Figure 19(c), showing the thickness of each electrodeposited layer. By fixing the top (bottom) Au layer thickness as 15 nm (40 nm) and setting Cu_2_O layer thickness respectively to 70 nm, 45 nm, and 20 nm, three reflection colors (cyan, magenta, and yellow) are realized. Measured reflection spectra from the fabricated samples show good correspondence to numerically calculated ones (Figure 19(d)). Benefiting from the high refractive index of Cu_2_O (*n* ∼ 2.5 in the visible range), the reflection spectra show robust angular insensitivity. In addition, to demonstrate the technique’s capability of depositing onto irregular-shape surfaces, the Au/Cu_2_O/Au stacks are coated onto stainless-steel spoons. Figure 19(e) shows the glossy and uniform colors from the layers on the curved surfaces of spoons.

**Self-assembly**. Self-assembly method can be regarded as a process to organize components of a system (such as molecules, polymers, colloids, and macroscopic particles) into ordered or functional structures using local interactions among these components [214–216]. Self-assembly approaches have been shown as simple and cost-effective methods for scalable production of structural color devices [217–220].

**Figure 20:**
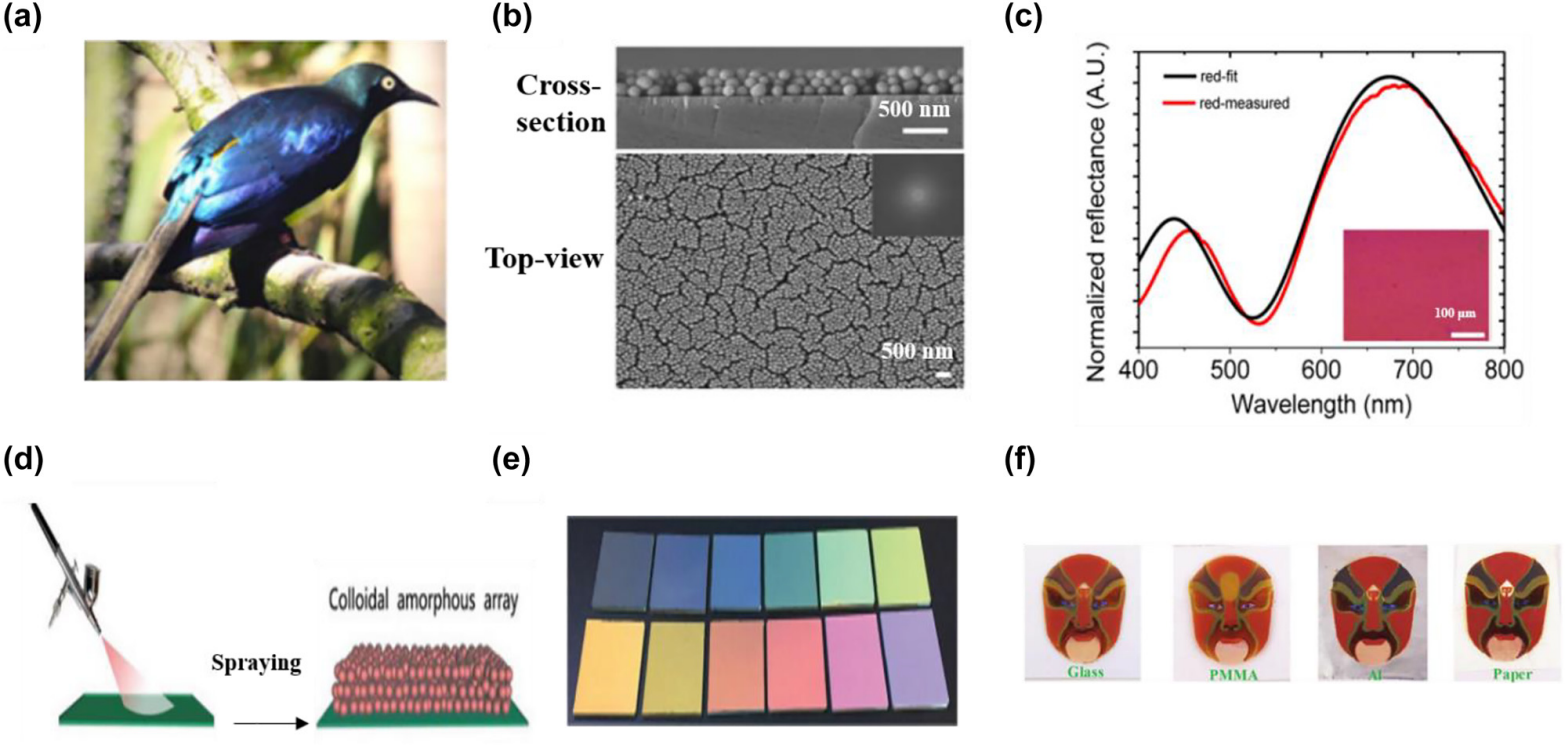
Self-assembly approach for fabricating multi-layer thin film based structural colors. (a) Photo of a bird. The color in its feather is produced by the self-assembled melanosomes. (b) Cross-sectional and top-view SEM images of a fabricated sample composed of synthetic melanin nanoparticle films. (c) Measured (red curve) and simulated (black curve) reflection spectra of a red-color sample under normal incidence. Inset shows the associated optical image. Modified with permission [217]. Copyright 2015, American Chemical Society. (d) Schematic of the fabrication process of PDA@SiO_2_ array via the spraying-drying method. (e) Optical images of the fabricated devices. Particle sizes of the PDA@SiO_2_ are around 172, 193, 204, 215, 232, and 252 nm (upper panel, left to right) and 264, 273, 286, 291, 309, and 323 nm (lower panel, left to right). (f) Photos of multicolor Chinese opera facial makeups fabricated on four different substrates. Reproduced with permission [218]. Copyright 2018, Royal Society of Chemistry.

Inspired by the self-assembled melanosomes in avian feathers which produce vivid colors (Figure 20(a)), Xiao and co-workers construct colored thin films through an evaporation based self-assembly process [217]. The device fabrication process consists of two main steps, which are synthetic melanin nanoparticle (SMNP) synthesis and evaporation based self-assembly. The SMNPs are synthesized through oxidation and self-polymerization of dopamine molecules at room temperatures in a solution consisting of water, ethanol, and ammonia. The sizes of synthesized SMNPs are determined by the molar ratio of ammonia and dopamine hydrochloride. In the self-assembly step, a clean silicon wafer is held vertically in the prepared solution at 60 °C until all its solvent contents are evaporated. A coated SNMP layer with an averaged particle diameter of 146 ± 15 nm is then obtained, and the associated SEMs are displayed in Figure 20(b). Nanometer-scale cracks, which result from film shrinkage during the evaporation process, are found to be evenly distributed in the obtained film. Measured (red curve) and simulated (black curve) reflectance spectra of a fabricated red-color sample are displayed in Figure 20(c). The two curves show good correspondence with each other, both of which exhibit two distinct peaks in the visible range with the primary one centered around 675 nm.

Liu and co-workers demonstrate another efficient approach for constructing non-iridescent structural colors by assembling self-adhesive poly-dopamine (PDA) coated SiO_2_ nanoparticles (PDA@SiO_2_) via a spraying-drying method (Figure 20(d)) [218]. PDA, produced by the autoxidation of dopamine (DA), can adhere to most commonly-used substrates with high strength. Different colors can be generated by altering the size of the silica core, content of the PDA, as well as arrangement of the PDA@SiO_2_. Optical images of fabricated samples are displayed in Figure 20(e), where the samples exhibit non-iridescent structural colors with hue ranging from blue to purple-red as the size of PDA@SiO_2_ increases. Multicolor patterns of a Chinese opera facial makeup are fabricated on four different substrates using this method (Figure 20(f)), showing the method’s good compatibility with various substrate types.

**Monolithic color integration**. One way to create different colors over the same substrate using the multilayer thin film structures (i.e., realizing monolithic color integration) is to create stepwise micro- or nano-cavities. Towards such goal, grayscale lithography [221–224] and nanoimprint lithography [225–230] have been exploited. During the device fabrication process, a thickness-modulated photoresist is commonly employed either as the dielectric layer in the cavity or as the etching mask to transfer the stepwise thickness profile onto the underneath dielectric layer.

**Figure 21:**
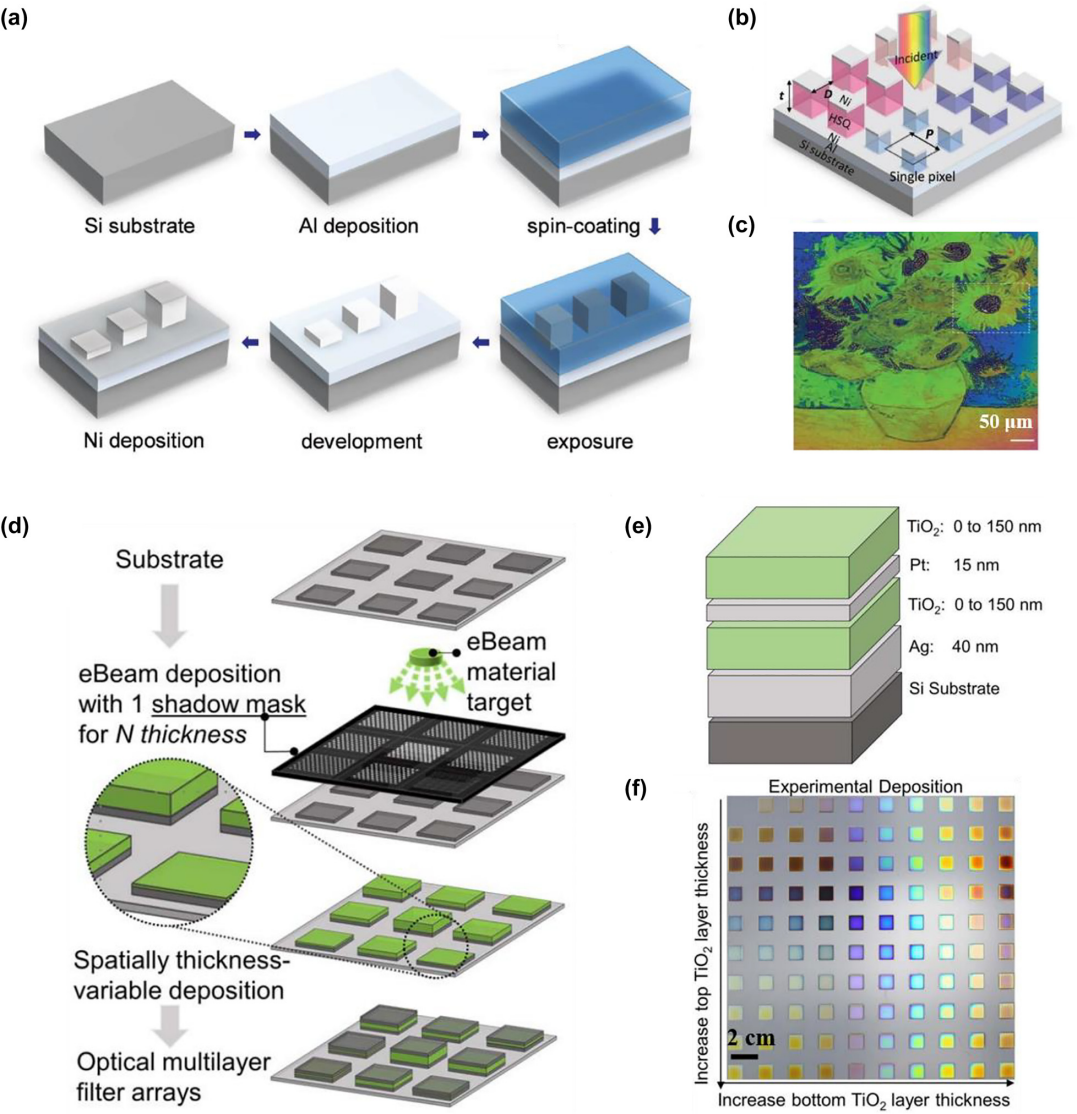
Grayscale lithography for monolithic color integration using multilayer thin film structures. (a) Schematic illustration of the direct write grayscale lithography for fabricating FP cavities with different cavity thicknesses. (b) Schematic diagram of monolithic full-color printing based on grayscale-patterned asymmetric FP resonators. Each individual color pixel is composed of an Al back reflector, an HSQ dielectric layer, and a Ni top layer. (c) Full-color reproduction of Vincent van Gogh’s “Still Life: Vase with Twelve Sunflowers”. Reproduced with permission [231]. Copyright 2017, Wiley-VCH. (d) Schematic representation of the grayscale stencil lithography which can generate patterns of spatially-varying thicknesses with a single shadow mask. (e) Schematic diagram of the FP cavity based device, which is composed of TiO_2_/Pt/TiO_2_/Ag stacked thin films on a Si substrate. (f) Experimental deposition results of reflective color arrays. Reproduced with permission [232]. Copyright 2020, Optica Publishing Group.

Grayscale lithography is a commonly used approach to produce three-dimensional (3D) structures at both the micro- and nano-scale. Figure 21(a) illustrates the manufacturing flow of a full-color FP-based device using the direct-write grayscale lithography [231]. Each individual color pixel is composed of a bottom Al mirror, a middle HSQ layer, and a top Ni layer (Figure 21(b)). Different colors are obtained by adjusting thickness of the middle HSQ dielectric layer (i.e., the device’s cavity length). The bottom Al layer is first deposited onto a Si substrate through electron-beam evaporation. Then, a layer of negative tone electron beam resist (hydrogen silsesquioxane-HSQ) is spun coated over the Al layer. By setting a proper exposure dose during the grayscale patterning process and optimizing the subsequent development condition, HSQ layers with different thicknesses are patterned simultaneously. Finally, a thin Ni layer is deposited on the substrate. Using this method, a full-color microscale painting which reproduces of Vincent van Gogh’s “Still Life: Vase with Twelve Sunflowers” is obtained (Figure 21(c)).

Besides the direct-write grayscale lithography, grayscale stencil lithography with a single shadow mask has been used for creating stepwise cavity thicknesses for multilayer thin film based structural colors (Figure 21(d)) [232]. Steady or movable shadow masks made of semiconductors or metals are used to define the open areas for material deposition. The total amount of deposited materials over a certain substrate area is determined by the filling ratio of aperture arrays in the customized stencil. Multispectral reflective structural color filter arrays have been successfully fabricated using this method. Figure 21(e) shows the configuration of this multilayer device, which consists of TiO_2_/Pt/TiO_2_/Ag thin films on a Si substrate. The thicknesses of both TiO_2_ layers can be tuned from 0 to 150 nm during fabrication, generating a palette with a wide range of reflective colors (Figure 21(f)).

**Figure 22:**
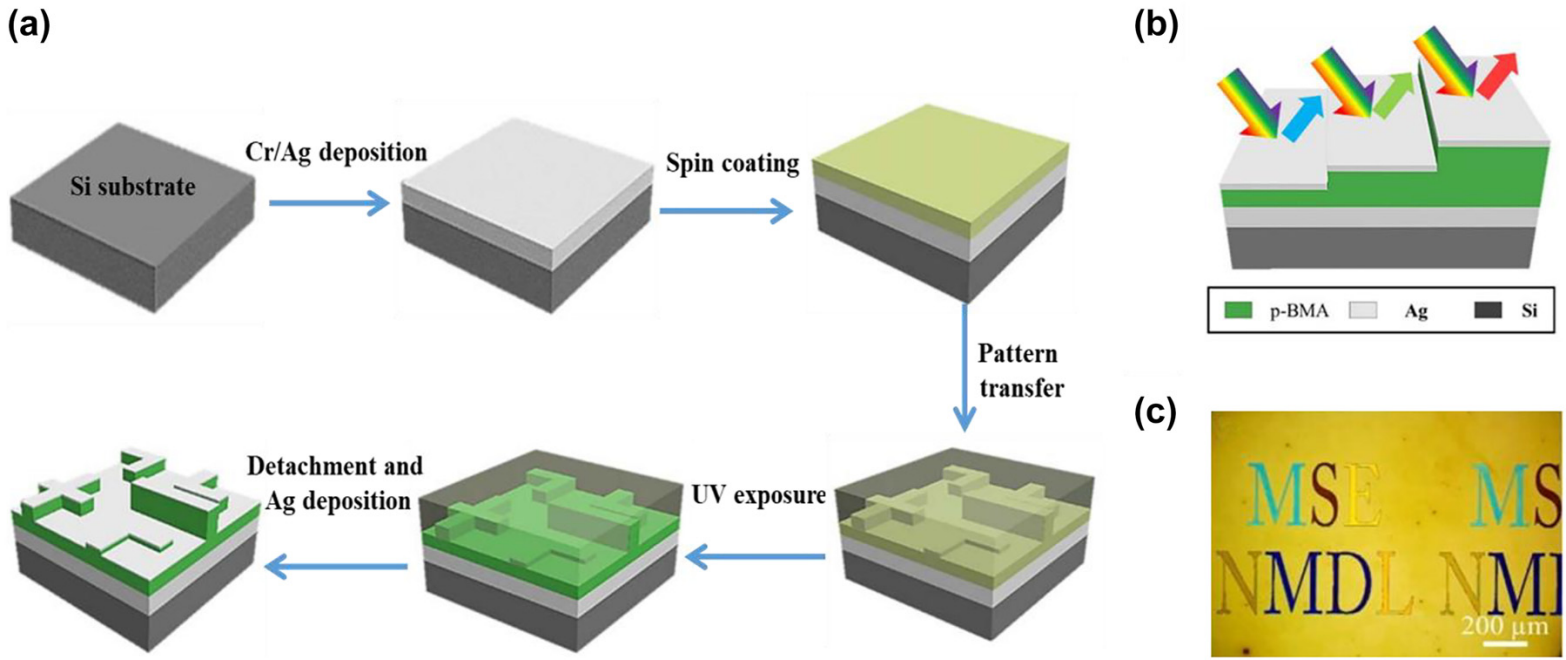
UV nanoimprint lithography (NIL) for monolithic color integration using multilayer thin film structures. (a) Fabrication process of FP cavities with different cavity thicknesses using NIL. (b) Schematic representation of the multicolor asymmetric FP absorbers integrated on the same substrate. Each absorber consists of a top semitransparent metallic layer (Ag), a middle dielectric layer of spatially-varying thickness (p-BMA (poly benzyl methacrylate)), and a bottom reflection layer (Ag). (c) Photo of a fabricated sample in the form of colored letters. Modified with permission [233]. Copyright 2020, IOP Publishing.

Nanoimprint lithography (NIL) transfers patterns from the mold into the target material through mechanical pressing, with the aid of heating or ultraviolet irradiation. By preparing molds with spatially-varying depth profiles, NIL can be utilized for fabricating FP-based structural color devices with diverse dielectric thicknesses on the same substrate. Baek and co-workers employ NIL to fabricate FP-based structural colors (Figure 22(a)), which are composed of two Ag mirrors and a dielectric medium sandwiched in between (Figure 22(b)) [233]. Key fabrication steps include bottom Cr/Ag layer deposition onto the Si substrate, pattern transfer through UV NIL, and top Ag layer deposition. Photo of a fabricated device in the form of colored letters is displayed in Figure 22(c). In addition, roll-to-roll (R2R) NIL has attracted great attention by its advantages of reduced cost and enhanced throughput compared with conventional NIL [234–236], and has shown great potential in creating large-scale structural color devices. Recently, a R2R-based physical vapor deposition process has been employed for manufacturing the “structural blue paint” used in Toyota Lexus LC Structural Blue Edition [237, 238].

### 5.2 Subwavelength nanostructure array based structural colors

Electron-beam lithography (EBL) followed by thin-film deposition and (or) etching is one of the most frequently used methods to fabricate subwavelength nanostructure based structural color devices [239–242]. In such process, patterns of the designed nanostructures are first created in the resist layer by EBL. Subsequently, those patterns are transferred to the device’s constituent materials through lift-off, etching, or a combination of two. Recently, a resist-based Damascene process has been demonstrated to fabricate high-aspect-ratio dielectric metasurfaces, where the device pattern is first defined in the resist layer through EBL, and then transferred to the dielectric material through low-temperature atomic layer deposition (ALD) followed by back-etching [243–247]. Although EBL exhibits great flexibility and high resolution in fabricating devices of various structures and materials, its high cost and low throughput have impeded the method’s usage for many practical applications. To mitigate the above issues, several alternative methods have been exploited for fabricating subwavelength nanostructure based structural color devices. Examples include self-assembly, nanoimprint lithography, fluid-guided printing, and digital inkjet printing. We will give a detailed discussion in the following parts.

**Figure 23:**
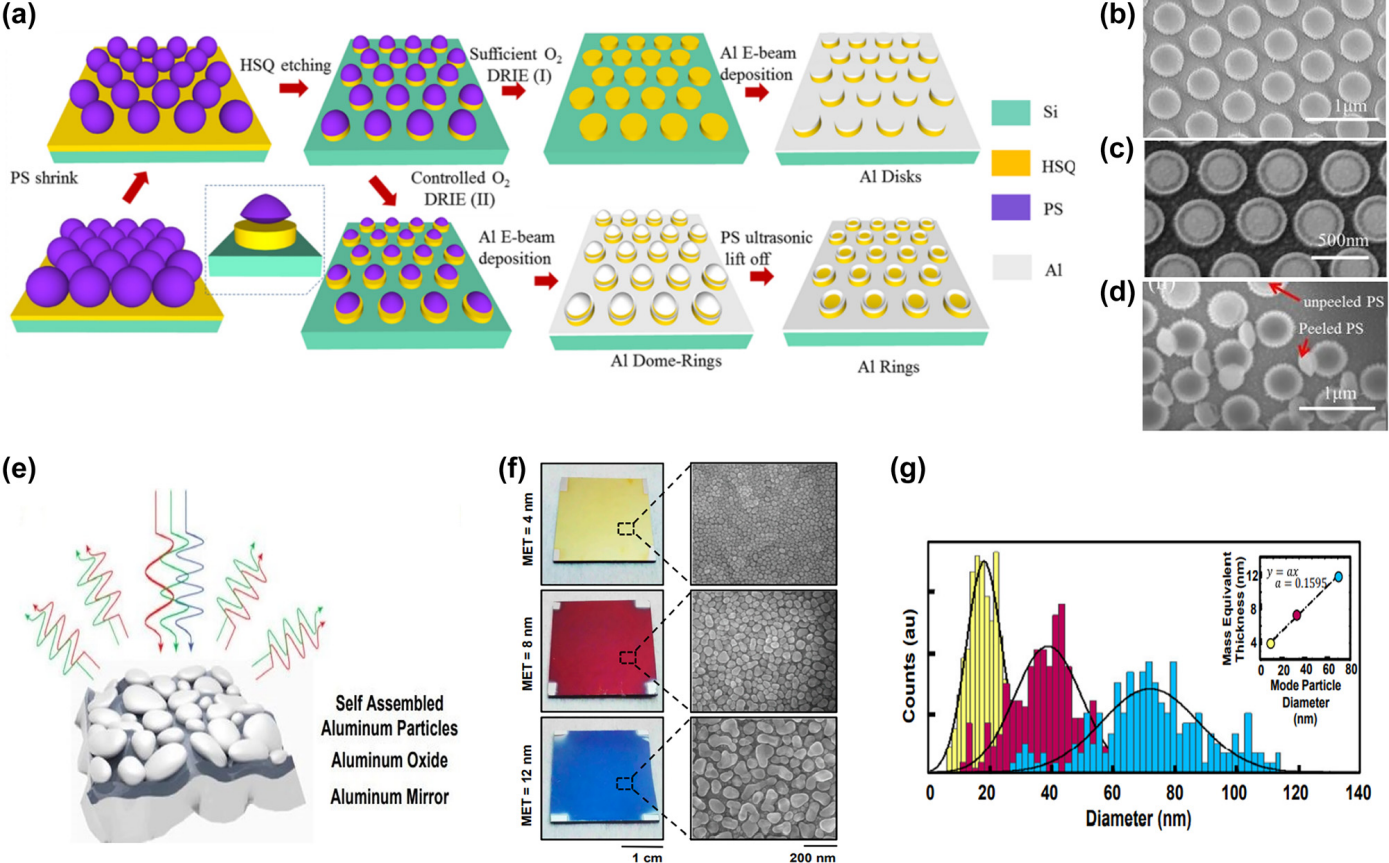
Self-assembly approach for fabricating subwavelength nanostructure array based structural colors. (a) Schematic flow chart for creating arrays of Al disks, dome-rings, and rings assisted by a bottom-up self-assembly process. (b)–(d) SEM images of the fabricated Al disks, dome-dings, and dings, respectively. Modified with permission [252]. Copyright 2016, American Chemical Society. (e) Schematic representation of self-assembled Al particles atop an oxide spacer and Al mirror. (f) Photos (left column) and SEM images (right column) of three samples respectively exhibiting yellow, magenta, and cyan colors. (g) Histogram of the particle sizes obtained from the fabricated samples. Reproduced with permission [253]. Copyright 2020, National Academy of Sciences.

**Self-assembly**. In addition to fabricating planar multilayer thin film structures, cost-effective self-assembly method can also be utilized to create large-area periodic subwavelength nanostructures [248–251]. Figure 23(a) shows the fabrication process of arrays of Al-based disks, dome-rings, and rings using a colloidal self-assembly method [252]. In this process, HSQ is first spin-coated onto a Si substrate. Then a self-assembled monolayer of polystyrene (PS) spheres is formed atop the spin-coated HSQ film. Afterwards, oxygen-based deep RIE is performed using the PS spheres as etching mask to remove the HSQ in the exposed area over the substrate. Depending on the etching condition, the PS spheres can be fully removed or partially etched. Subsequently, a 15 nm-thick Al layer is deposited on the substrate to create Al disk or Al dome-ring arrays. By sonicating the dome-ring sample in DI wafer for ∼30 min, Al ring arrays can be obtained. SEMs of fabricated samples are displayed in Figure 23(b)–23(d), showing periodically arranged Al disks, dome-rings, and rings, respectively.

Self-assembled metallic nanoparticles can also exhibit colors with robust angular sensitivity and high purity [253]. Figure 23(e) shows a plasmonic color device having a dense array of Al nanoparticles formed atop an oxide-coated Al backplate. Arrays of closely-packed Al particles are created in an ultrahigh vacuum electron beam evaporator through a temperature and pressure dependent thin film growth mechanism. Shapes of the obtained particles depend on the equilibrium condition between the Al’s free energy and interfacial stress with substrate. Large-scale samples showing yellow, magenta, and cyan colors are fabricated (Figure 23(f), left panel), of which the associated mass equivalent thicknesses are, respectively, 4 nm, 8 nm, and 12 nm. As observed from both the SEMs (Figure 23(f), right panel) and particle size histograms (Figure 23(g)), the size of Al particles increases as the sample’s reflection spectrum moves to the longer wavelength range.

**Figure 24:**
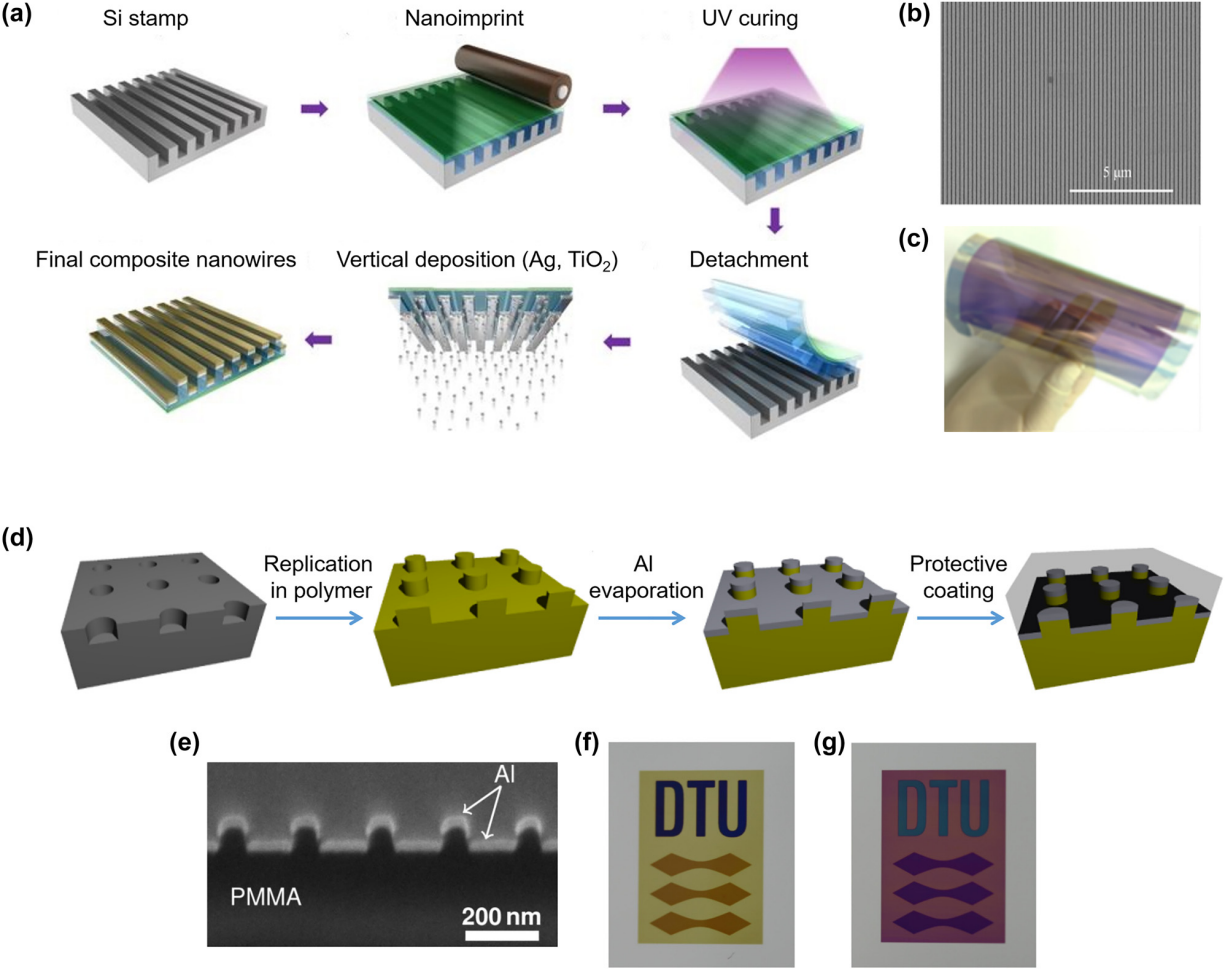
Nanoimprint lithography for fabricating subwavelength nanostructure array based structural colors. (a) Fabrication process of large-scale, flexible, and polarization-dependent structural colors with composite nanowire structures. (b) and (c) SEM and photo of a fabricated sample. Modified with permission [256]. Copyright 2018, American Chemical Society. (d) Fabrication scheme of 2D plasmonic nanostructures. (e) Cross-sectional SEM image of a fabricated sample, showing the PMMA polymer pillar array covered by a metallic Al layer. (f) and (g) Optical images of a fabricated sample without (f) and with (g) the protective coating (Ormocomp, Micro resist technology GmbH). Modified with permission [257]. Copyright 2014, American Chemical Society.

**Nanoimprint lithography**. Nanoimprint lithography (NIL) can also be employed for fabricating nanostructure-based structural color devices [254, 255]. Flexible wafer-scale polarization-dependent structural color devices, which consist of Ag-TiO_2_ composite nanowires, are fabricated through NIL and electron-beam evaporation (Figure 24(a)) [256]. In the NIL process, imprint resin is first coated on the Si stamp having line patterns. Then, a polymethyl methacrylate (PMMA) film is covered on the resin. After UV curing, the whole PMMA-patterned resin structure is peeled off the Si stamp. Finally, Ag and TiO_2_ thin films are sequentially coated onto the patterned resin surface. SEM image of one fabricated sample and photo of a large-area color sample are respectively displayed in Figure 24(b) and (c). A similar fabrication process has been applied for creating 2D plasmonic color filters (Figure 24D), comprising Al metallic disks on top of dielectric nano-pillars (Figure 24(e)) [257]. A thick protective coating layer is deposited on top of the fabricated device to protect it against environmental damage. Photos of a colored sample before and after the coating are shown in Figure 24(f) and (g), respectively. Compared to the as-fabricated sample, the coating-protected sample shows a redshift in its color due to an increase in the effective refractive index of the nanostructures’ surrounding environment.

**Figure 25:**
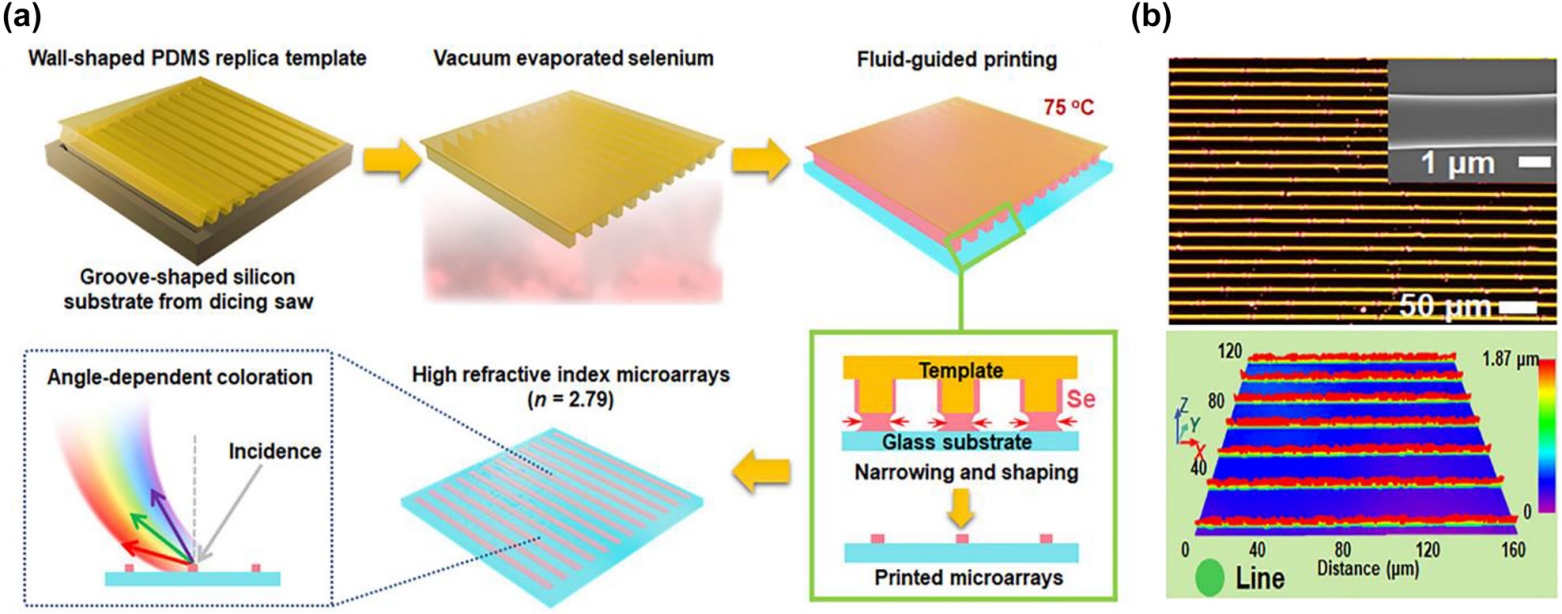
Fluid-guided printing for fabricating subwavelength nanostructure array based structural colors. (a) Schematic illustration of the fluid-guided printing process for preparing selenium microarrays. The fabricated device exhibits an angle-dependent coloration appearance. (b) Top panel: dark-field optical microscope image of the Se microline arrays. Inset: the associated SEM image. Bottom panel: 3D optical profile image of a fabricated sample. Modified with permission [259]. Copyright 2021, Elsevier.

**Fluid-guided printing**. Fluid-guided printing [258, 259] is another method for fabricating large-scale structural color devices. The fabrication flow of a Selenium (Se)-based structural color device is illustrated in Figure 25(a) [259]. First, a flexible PDMS template is replicated from a groove-shaped Si substrate. Then, Se is evaporated onto the patterned PDMS template. When Se is heated above its glass transition temperature, the resulting material flow leads to the structure’s downsizing and shaping with the guidance of the template. After cooling down to room temperature for ∼5 min, high refractive index (*n* ∼ 2.79) Se microline arrays are formed on the substrate. The sample’s reflection spectrum exhibits a high angular sensitivity due to the high refractive index of Se and the associated strong optical confinement effect inside the fabricated structure. The sample’s dark-field optical microscopy image and 3D height profile are shown in Figure 25(b), showing the structure’s uniform orientation and smooth edge.

**Figure 26:**
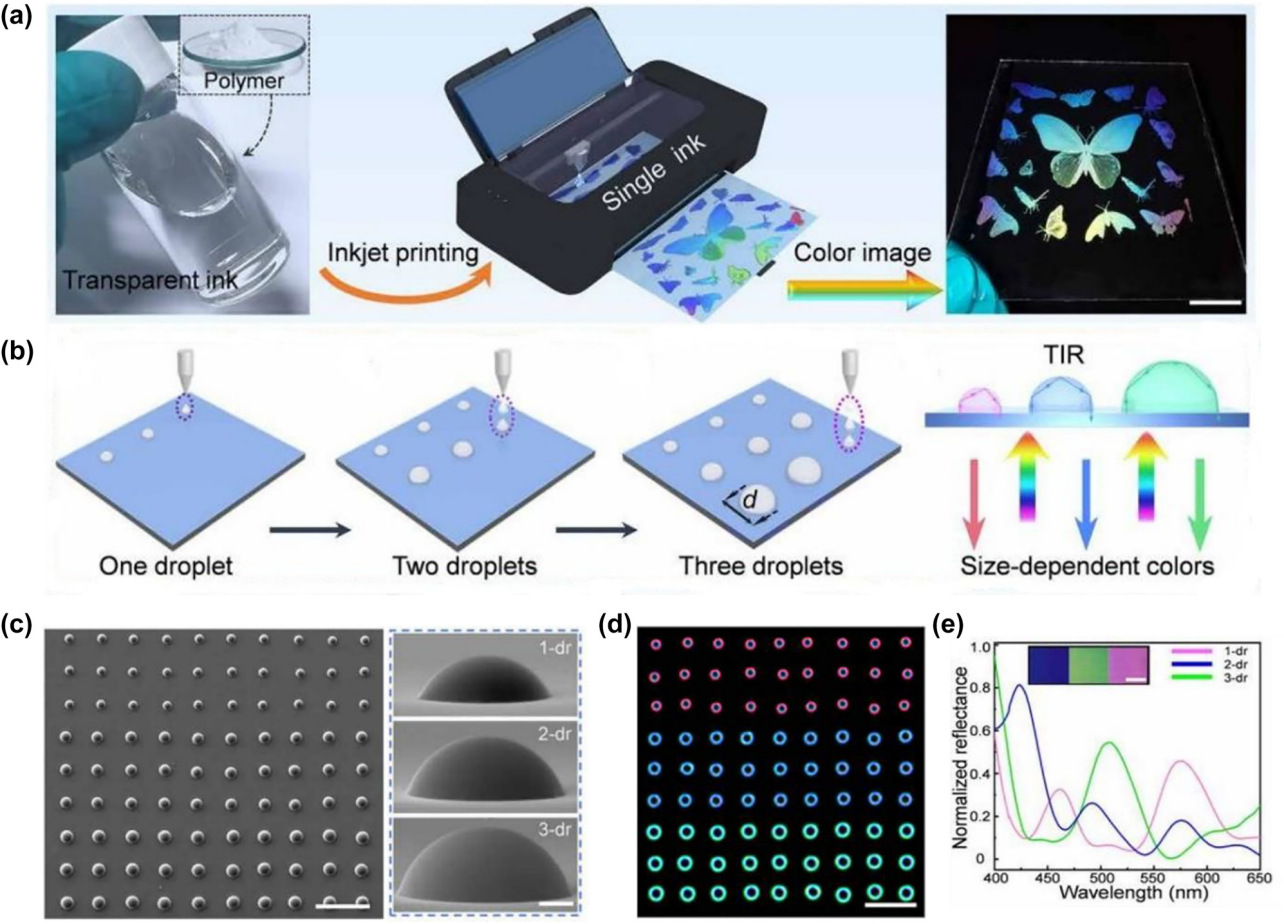
Digital inkjet printing for fabricating subwavelength nanostructure array based structural colors. (a) The process of structural-color printing with a single transparent polymer ink. (b) Schematic illustration of the droplet-by droplet printing process. (c) Top-view (left) and cross-sectional (right) SEM images of printed microdomes with different diameters. (d) Dark-field optical micrographs of the microdomes observed from the bare-glass side. (e) Reflection spectra of the printed structural-color panel at the macroscale. Inset shows the associated optical images. Scale bar: 1 mm. Modified with permission [263]. Copyright 2021, American Association for the Advancement of Science.

**Digital inkjet printing**. Digital inkjet printing [260–262] with transparent inks has also been exploited for creating full-color and high-photorealistic structural color devices (Figure 26(a)) [263]. During the printing process, microdomes with different sizes are prepared by a droplet-by-droplet method (Figure 26(b)). Controlling both the ink volume and the substrate wettability leads to the total internal reflection (TIR) effect inside the microdome occurring at different wavelengths, which consequently results in different reflection colors from the printed sample. Vivid structural colors with controlled gamut, saturation and brightness have been realized by properly programming the arrangement of different microdomes during the inkjet printing process. Figure 26(c) shows the top-view and cross-sectional SEM images of printed microdome arrays with different diameters, which all exhibit a symmetrical morphology with smooth surface. Figure 26(d) shows the optical images of generated colors (red, blue, and green) from arrays of microdomes with diameters of 16.2 μm, 20.3 μm, and 23.5 μm, respectively. Measured reflection spectra are displayed in Figure 26(e).

## 6 Dynamic structural color generation

Dynamic devices with tunable color generating functionalities can further expand the application of structural color technology, and thus, have become a hot research topic over recent years. As discussed earlier, the reflection (transmission) spectrum of a color-generating nanostructure can be affected by various factors, including state of polarization of the incident light, refractive index of its constituent material, as well as geometric configuration of the nanostructure. Based on this principle, researchers have demonstrated an array of tunable color-generating devices [25, 264, 265]. In the following part, we will survey some representative work.

**Figure 27:**
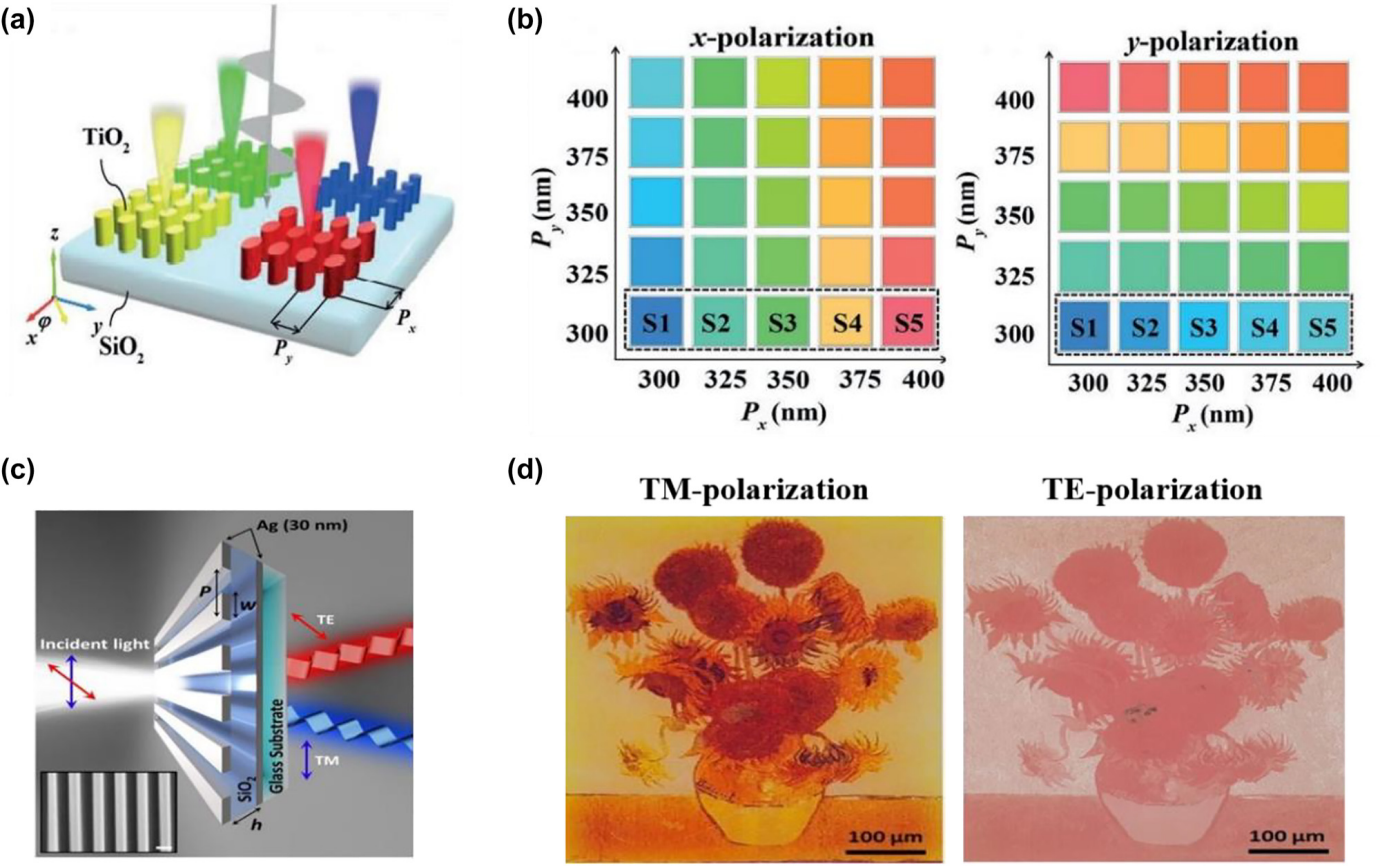
Dynamic structural color generation based on state of polarization of the incident light. (a) Schematic illustration of a polarization-dependent structural color device composed of arrays of TiO_2_ elliptic nanopillars on a silica substrate. Periods along the *x*- and *y*-directions are denoted as *P*_*x*_ and *P*_*y*_, respectively. (b) Color palettes under *x*- and *y*-polarized incidence with *P*_*x*_ and *P*_*y*_ varying from 300 nm to 400 nm. The nanopillar height is fixed as 300 nm and the filling ratios along the two axes are fixed as 0.6. Modified with permission [268]. Copyright 2018, Wiley-VCH. (c) Schematic of a polarization-selective color device, which is composed of an Ag mirror, a 1D Ag nano-grating layer, and a SiO_2_ dielectric layer in between. TM polarization refers to electric field vector oscillating orthogonal to the grating direction, while TE polarization refers to electric field along the grating direction. (d) Optical images of a fabricated sample (showing Vincent van Gogh’s Sunflower) under TM- and TE-polarized illumination. Modified with permission [271]. Copyright 2020, American Chemical Society.

**State of polarization of the incident light.** Tuning the state of polarization of the incident light is one of the most straightforward approaches to realize dynamically tunable structural colors generated by asymmetric nanostructures. The dynamic tunability in colors stems from the structures’ diverse resonant responses to incident light with different polarization states. There has been an array of reports on polarization-based dynamic structural color generation [266–270]. For instance, Yang and co-workers propose a polarization-sensitive structural color metasurface composed of arrays of TiO_2_ elliptical nano-pixels on a silica substrate (Figure 27(a)) [268]. Various simulated colors from the nano-pixel arrays with different periodicities under *x*- and *y*-polarized illumination are displayed in Figure 27(b). Here, the periods of the nano-pixel array along *x*- and *y*-axes (*P*_*x*_ and *P*_*y*_) vary from 300 nm to 400 nm, and the filling ratios are fixed at 0.6. A sharp color contrast is shown under the two orthogonal polarizations for each array. For example, when *P*_*y*_ is fixed at 300 nm and *P*_*x*_ gradually increases from 300 nm to 400 nm, the reflection color under *x*-polarized illumination changes from blue to green and then to red, whereas the color under *y*-polarized illumination remains blueish.

Figure 27(c) shows another polarization-dependent structural color device composed of a Ag mirror, 1D Ag nano-gratings, and a SiO_2_ dielectric layer in between [271]. Strong surface plasmon resonance occurs for TM polarized incident light (electric field orthogonal to the grating direction), whereas no distinct resonance is generated for TE polarized illumination (electric field along the grating direction). Under these two polarization states, two different transmission colors are realized. A fabricated sample (showing Vincent van Gogh’s Sunflower) changes from a bright multicolor image to a pale single-color image as the polarization state changes from TM to TE (Figure 27(d)).

**Refractive index modulation**. The optical properties (real (*n*) and imaginary (*κ*) part of the refractive index) of certain materials, including liquid crystals, electrochromic polymers, phase-change materials, and phase transition metal hydrides, can be modulated by external bias voltage, heating, or the hydrogenation/oxidation treatment.

**Figure 28:**
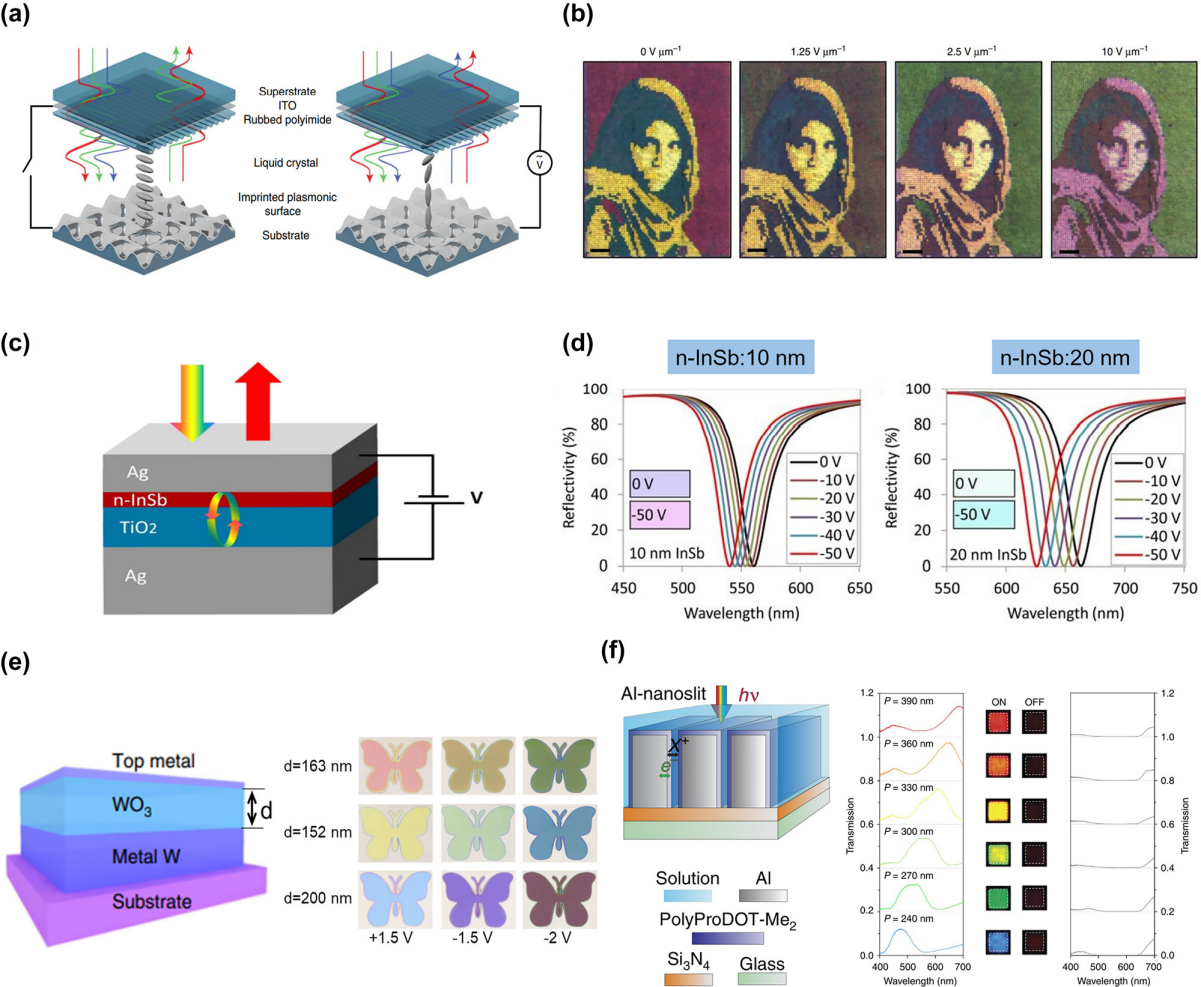
Dynamic structural color generation based on refractive index modulation through external bias voltage. (a) Schematic diagram of the plasmonic liquid crystal (LC) cell. An applied electric field across the cell reorients the liquid crystal and changes wavelengths of the absorbed light. (b) Microscope images of the Afghan Girl, showing reflection colors varying with the applied electric field. Scale bar: 100 μm. Modified with permission [279]. Copyright 2015, Nature Publishing Group. (c) Schematic diagram of an epsilon-near-zero (ENZ) material based tunable structural color device, consisting of stacked Ag/n-InSb/TiO_2_/Ag films. (d) Calculated reflection spectra from two devices (each with a 10 nm or 20 nm thick n-InSb layer) under different bias voltage levels. Insets show the predicted colors. Reproduced with permission [289]. Copyright 2018, Nature Publishing Group. (e) Left panel: schematic illustration of the inorganic electrochromic material (WO_3_) based tunable device, composed of a thick tungsten (W) bottom reflector, a WO_3_ middle layer, and a thin metal (Ag or W) top mirror. Right panel: Different color states of three butterfly-shape electrochromic devices under applied voltages of 1.5, −1.5, and −2 V. For the three devices, thickness of the WO_3_ cavity layer is respectively chosen as 163 nm, 152 nm, and 200 nm. Modified with permission [293]. Copyright 2020, Nature Publishing Group. (f) Left panel: Schematic diagram of the high-contrast full-color EC switching device composed of periodic arrays of Al nanoslits conformally coated with a thin PolyProDOT-Me_2_ layer. Right panel: Experimental results of the optical transmission spectra along with corresponding optical micrographs. The electrochromic PolyProDoT-Me_2_ layer coated on the Al nanoslit array is electrically switched between a reduced (ON) and an oxidized (OFF) state. Periods of the Al nanoslits vary from 240 nm to 390 nm with a step of 30 nm. Modified with permission [296]. Copyright 2016, Nature Publishing Group.

Liquid crystals (LCs) are anisotropic particles whose arrangement can be altered from a random orientation to a fixed one via an applied bias voltage, leading to a substantial refractive index change [272–276]. Such property has been utilized to construct tunable structural color devices [277–281]. Franklin and co-workers report a color-tunable device composed of periodic arrays of shallow nanowells and high birefringence LCs (Figure 28(a)) [279]. Dynamic colors are realized when adjusting the applied electric field (0–10 V μm^−1^). As shown in Figure 28(b), the reflective optical image (the Afghan Girl) exhibits different colors at different electric fields.

Epsilon-near-zero (ENZ) materials [282–288] exhibit significant changes in their refractive indices under an external bias voltage, and can be employed as constituent layers in electrically-tunable structural color devices. N-type doped indium antimonide (InSb), whose real-part permittivity curve exhibits a close-to-zero point in the visible region, is one representative ENZ material. A thin InSb layer experiences an increase in its carrier density under an external bias voltage, and consequently, a variation in its complex refractive index as well as ENZ wavelength over the visible. The schematic diagram of an FP-cavity-based device incorporating a thin InSb absorbing layer is shown in Figure 28(c) [289]. A compound dielectric layer made of an N-type InSb layer (of varying thickness from 10 nm to 20 nm) and a TiO_2_ layer (40 nm), together with a top thin Ag reflector (35 nm) and a bottom thick Ag mirror (100 nm), form an FP cavity generating reflection-type colors. The device’s reflection spectrum changes with the applied external bias voltage (Figure 28(d)).

Electrochromic (EC) materials [290–292] can support reversible refractive index modulations through electrically controlled redox reactions. During the reduction (gain of electrons) or oxidation (loss of electrons) process, electronic properties and refractive indices of these EC materials are considerably altered. Tungsten trioxide (WO_3_), as one widely used inorganic EC material, has been employed as the dielectric layer in an FP-cavity-based structural color device (Figure 28(e)) [293]. The proposed FP cavity is made by a thick tungsten (W) bottom reflector, a WO_3_ middle layer, and a thin metal (W or Ag) top mirror. By setting the WO_3_ layer thickness respectively as 163 nm, 152 nm, and 200 nm, three basic colors (i.e., red, yellow, and blue) are generated. Moreover, the created colors can be readily tuned by applying different bias voltages. Another FP-cavity-based device has been demonstrated for creating tunable colors, using organic poly-thieno [3,4-b]thiophene (pT34bT) polymer as the index-tuning dielectric spacer [294]. EC polymers combined with plasmonic nanostructures offer another approach for dynamic color generation [295–297]. Xu and co-workers achieve high-contrast full-color switching by employing poly(2,2-dimethyl-3,4propylenedioxythiophene) (PolyProDOT-Me_2_)) as the EC polymer (Figure 28(f)) [296]. Periodic arrays of Al nanoslits are conformally coated with a thin layer (∼15 nm) of PolyProDOT-Me_2_, and a 170 nm-thick Si_3_N_4_ layer is added beneath the Al nanoslits to further narrow the spectrum linewidth. The whole device is immersed in an electrolyte solution, along with a counter electrode (Pt) and a reference electrode. Electrons from the metal and ions from the electrolyte are injected into (reduction) or removed from (oxidation) the EC polymer when different voltages are applied. At the oxidized state (“OFF”), PolyProDOT-Me2 exhibits a broadband absorption in the visible, resulting in a uniform black color. At the reduced state (“ON”), PolyProDOT-Me2 is transparent. The incident light will pass through the EC polymer and excite the coupled plasmonic modes propagating along the nanoslits, generating distinct colors.

**Figure 29:**
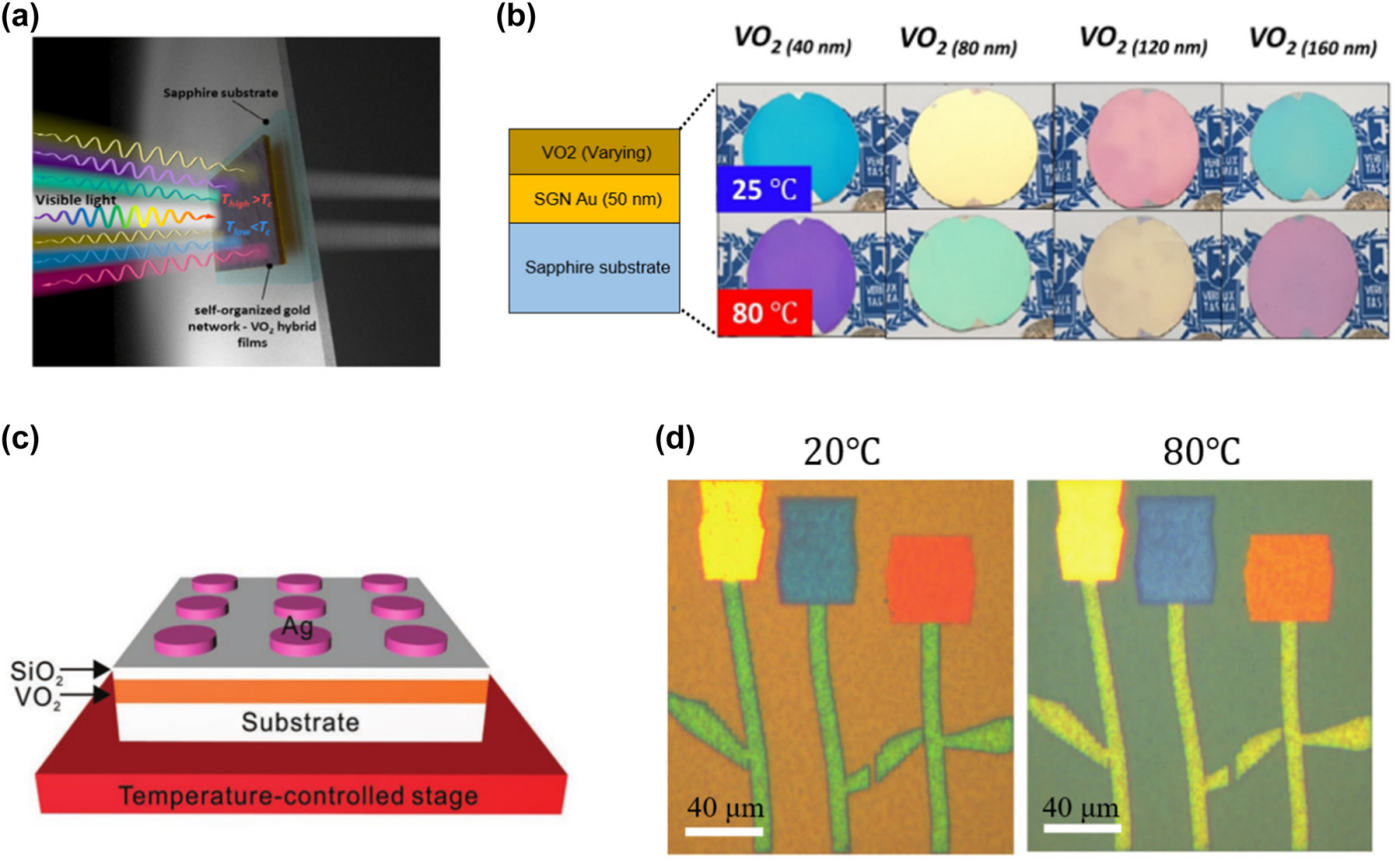
Dynamic structural color generation based on refractive index modulation through external heating. (a) Self-organized gold network–vanadium dioxide hybrid structure for dynamic structural color generation. (b) Photos of the hybrid structures with various VO_2_ thicknesses under different heating temperatures. Modified with permission [308]. Copyright 2020, Wiley-VCH. (c) Schematic diagram of a VO_2_-based tunable color device, composed of a periodic Ag nanodisk array, a SiO_2_ spacer layer, a planar VO_2_ film, and a glass substrate. (d) Images of a reflection-color sample at 20 °C and 80 °C. Modified with permission [310]. Copyright 2018, Wiley-VCH.

The phase transition in phase-change materials (PCMs) under external heating yields a significant variation in their optical permittivity [298–302]. Vanadium dioxide (VO_2_), one representative PCM, has gained great interest for tunable optoelectronic device applications by its low transition temperature, large hysteresis, and considerable refractive index contrast between different phases [303–307]. A bi-layer structure on sapphire substrate, consisting of a self-organized (SGN) Au network layer (50 nm) and VO_2_ thin film (of varying thickness) on top, has been demonstrated for generating thermally-tunable structural colors (Figure 29(a)) [308]. By setting the VO_2_ layer thickness respectively as 40 nm, 80 nm, 120 nm, and 160 nm, the device exhibits cyan, yellow, magenta, and emerald colors at room temperature (25 °C). When the device is heated to 80 °C, the VO_2_ layer undergoes an insulator-metal transition and experiences a large variation in its refractive index over the visible. Consequently, the afore-mentioned colors change to purple, aquamarine, yellow, and light-violet, respectively (Figure 29(b)). Another representative PCM, germanium telluride (GeTe), can also produce tunable reflective colors when integrated into a subwavelength layered optical cavity. For example, a device consisting of a 60 nm-thick magnesium fluoride (MgF_2_) AR coating, a 15 nm-thick GeTe layer, a 250 nm-thick SiO_2_ spacer, and a 100 nm-thick palladium/nickel chromium (Pd/NiCr) reflector is demonstrated to produce dynamic colors [301]. Results show that tunable colors ranging from red to blue are generated by adjusting the heating temperature from 165° to 190°.

Phase-change materials in combination with subwavelength nanostructures can also generate dynamic structural colors via an applied external heating [309–311]. For example, tunable colors are generated by integrating plasmonic nanostructures with a PCM (VO_2_) thin film. The device is composed of periodic Ag nanodisk array, a SiO_2_ spacer layer, a planar VO_2_ film, and a glass substrate (Figure 29(c)) [310]. The optical permittivity variation in VO_2_ induced by the phase transition yields a shift in the device’s resonant wavelength, leading to its tunable color appearance. A fabricated sample displays two different images as the temperature changes from 20 °C to 80 °C (Figure 29(d)).

**Figure 30:**
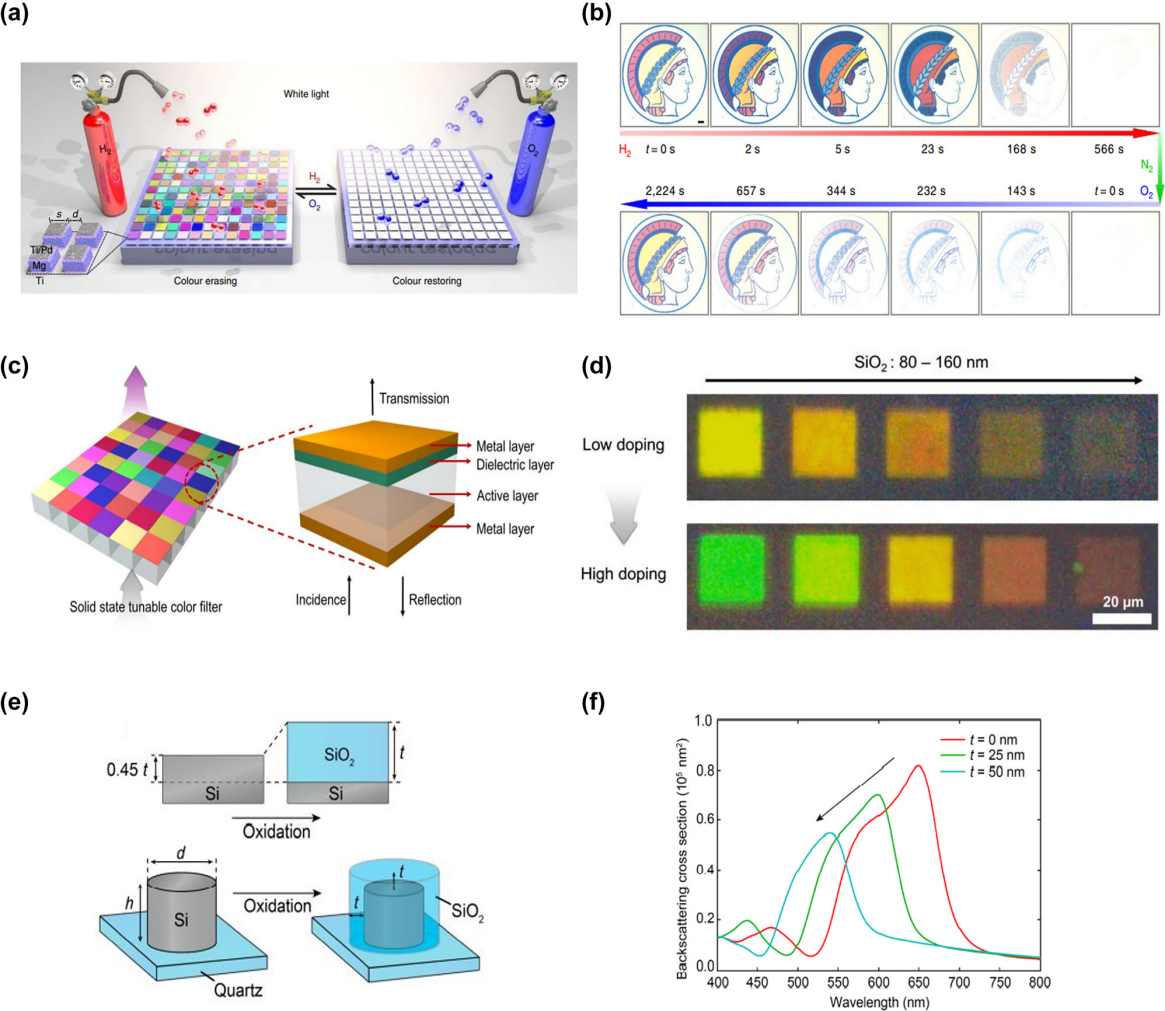
Dynamic structural color generation based on refractive index modulation through H_2_/O_2_ treatment. (a) Schematic illustration of the hydrogen-responsive plasmonic metasurface, in which each dynamic plasmonic pixel is composed of Mg nanoparticles sandwiched between Ti/Pd capping layers and a Ti buffer layer. (b) Optical micrographs of the Minerva logo, showing the color erasing and restoring performance during the hydrogenation and dehydrogenation process. Reproduced with permission [317]. Copyright 2017, Nature Publishing Group. (c) Schematic illustration of a tunable structural color design using H_2_ doped semiconductor. The top and bottom metallic layers are both Ag (30 nm), the index-changing layer is IGZO (40 nm), and the dielectric spacer layer is SiO_2_ (of varying thickness from 80 nm to 160 nm). (d) Experimental results of micro-sized printing under low and high doping conditions. The five devices are made of SiO_2_ layers of increasing thicknesses from 80 nm to 160 nm with a step of 20 nm. Reproduced with permission [318]. Copyright 2020, Chinese Laser Press. (e) Schematic diagram of an oxidized Si nanopatch structure. In order to form a SiO_2_ oxidation layer with thickness t in the Si nanostructure, it requires the consumption of Si domain with thickness 0.45*t* in both the lateral and vertical directions. (f) Backscattering spectra of three nanopatch structures, in which the diameter (*d*) and height (*h*) of Si antennas are all set as 150 nm and the oxidized layer thicknesses (*t*) are, respectively, set as 0 nm, 25 nm and 50 nm. Reproduced with permission [319]. Copyright 2018, American Chemical Society.

Dynamic structural colors can also be generated through the absorption/desorption of hydrogen or oxygen, in response to the marked changes in the intrinsic optical properties along with the metal-to-insulator transition. Among a variety of phase transition metal hydrides, Mg has attracted particular attention because of its good plasmonic response in the visible regime and reversible switching capability between the metallic (Mg) and dielectric (MgH_2_) states [312–316]. Figure 30(a) explains the concept of Mg-based dynamic color display technique [317]. Each plasmonic color pixel is composed of Ti/Pd capping layers, a Ti buffer layer, and Mg nanoparticles between them. When exposed to molecular hydrogen, the Pd capping layer catalyses hydrogen molecules into hydrogen atoms which then diffuse into the Mg particles through the Ti capping layer. As a result, dielectric MgH_2_ is formed. Figure 30(b) shows that the Mg-based vivid plasmonic structural colors are erased when all Mg nanoparticles are transformed into MgH_2_. And when exposed to oxygen, the dielectric MgH_2_ particles are converted back to the metallic Mg particles, leading to the generated colors again.

Refractive index modulation can also be realized by controlling the charge carrier concentration in an indium–gallium–zinc-oxide (IGZO) thin film [318]. Figure 30(c) depicts the structure of a tunable color device using IGZO as an index-changing layer. This transmission-type device consists of four layers, where the top and bottom Ag metallic mirrors are separated by a dielectric SiO_2_ layer and an active IGZO layer. The carrier concentration of the IGZO layer can be controlled through hydrogen plasma treatment, leading to variation in its refractive index over the visible range and subsequently change in the device’s transmission color. For example, when the IGZO layer thickness is fixed as 40 nm and the SiO_2_ layer thickness varies from 80 nm to 160 nm, the device exhibits colors ranging from yellow to red in the case of low-doping IZGO and green to red for high-doping IZGO (Figure 30(d)).

All-dielectric color control through Si oxidation, where Si reacts with oxygen to form SiO_2_, is demonstrated by Nagasaki and co-workers [319]. Figure 30(e) shows the Si oxidation process on a Si surface, where the Si domain is consumed along both the lateral and vertical directions. The conformal SiO_2_ layer becomes thicker as the oxidation time increases. The device’s resonant wavelength blue-shifts with the increase of oxidized layer thickness (Figure 30(f)).

**Figure 31:**
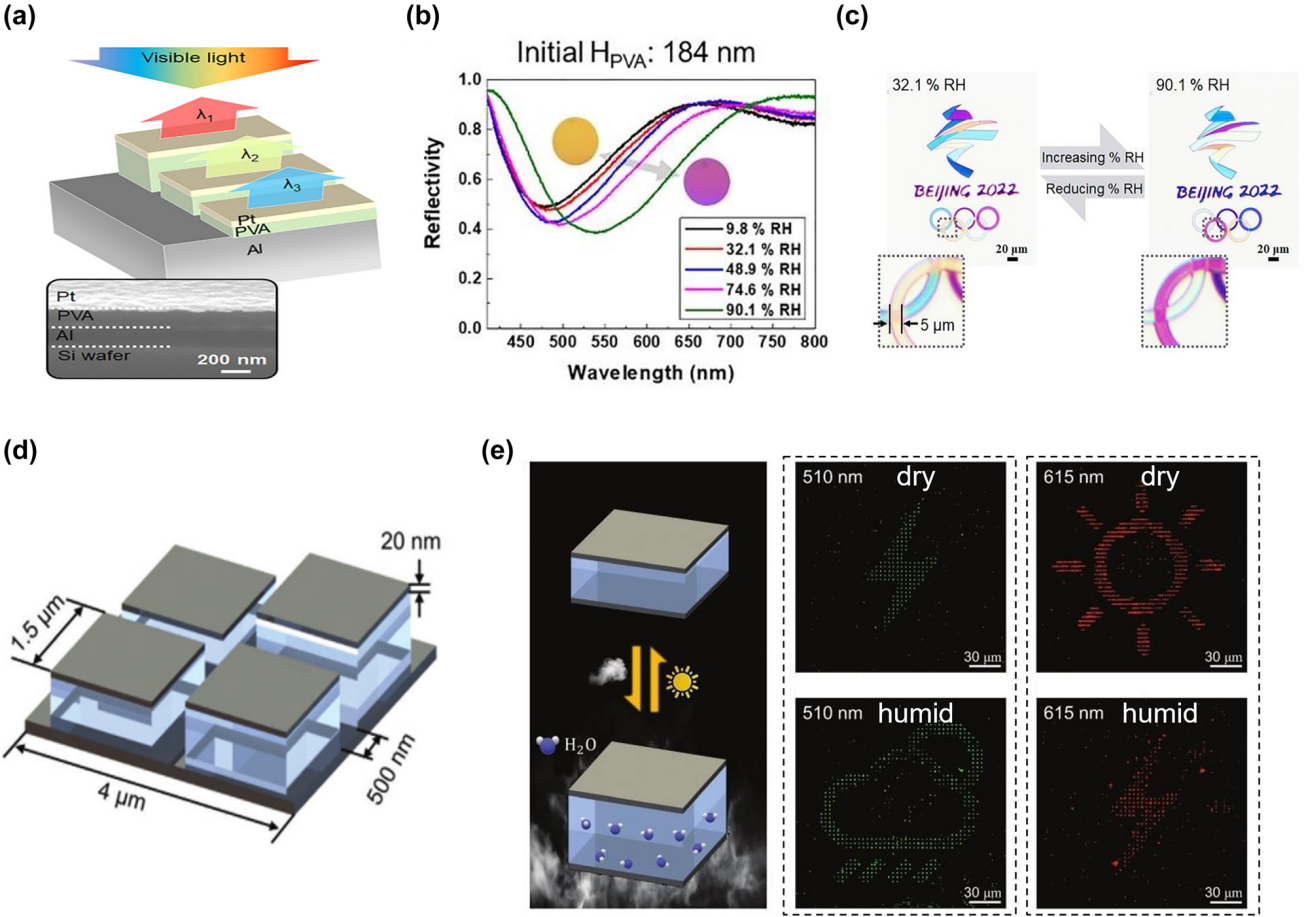
Dynamic structural color generation based on cavity length modulation using a humidity-swelling polyvinyl alcohol (PVA) layer. (a) Schematic diagram of a metal–insulator–metal (MIM) structure with spatially-varying insulator thickness for multi-color display. Inset shows the cross-sectional SEM image of stacked Pt/PVA/Al layers on a Si substrate. (b) Reflection spectra under normal incidence as the ambient humidity varies from 9.8% to 90.1% relative humidity (RH). Insets: corresponding colors at 32.1% and 90.1% RH. (c) Photos of a colored logo of the Beijing 2022 Olympic Games as the humidity is switched between 32.1% and 90.1% RH. Reproduced with permission [321]. Copyright 2021, Walter de Gruyter GmbH. (d) Schematic diagram of a super-pixel (4 × 4 μm^2^), comprising four stepwise metal–hydrogel–metal (MHM) sub-pixels (1.5 × 1.5 μm^2^) with different cavity (PVA) thicknesses. The thicknesses of both top and bottom Ag layers are 20 nm. (e) Experimentally captured, single-colored images of the PVA-based nano-printing under dry (top) and humid (bottom) conditions. Modified with permission [322]. Copyright 2022, Wiley-VCH.

**Nanostructure geometric configuration modulation.** As mentioned earlier, diverse structural colors across the entire visible band can be readily achieved by adjusting the cavity length of FP resonators. Therefore, employing materials with tunable thicknesses to form the FP cavity is one efficient approach for dynamic structural color generation [320]. Polyvinyl alcohol (PVA) hydrogel film, as one representative material with such property, exhibits a self-driven swelling and shrinking feature as the environment humidity varies. Figure 31(a) shows the schematic diagram of a PVA-based device for dynamic color generation, which is composed of a thick Al bottom reflector, a PVA dielectric layer, and a thin Pt top coating [321]. The stepwise thickness profile of the PVA layer is created by setting different exposure doses during the grayscale lithography process. An array of reflection colors covering the entire visible range are obtained by choosing proper PVA layer thicknesses. Furthermore, the PVA layer thickness can be effectively modulated as the ambient humidity varies, leading to dynamic color switching (Figure 31(b) and (c)).

Another dynamic switching of color display is also realized by utilizing the PVA core layer [322]. Figure 31(d) shows the schematic diagram of the proposed device, consisting of two 20 nm-thick Ag mirrors separated by a PVA layer of varying thickness. Red, green, and blue transmission colors are realized by setting PVA thickness as 145 nm, 115 nm, and 95 nm, respectively. Moreover, thickness of the PVA layer increases proportional to the ambient humidity, leading to a red shift in the device’s resonant wavelength and thereby displayed images with dynamic tunability (Figure 31(e)).

**Figure 32:**
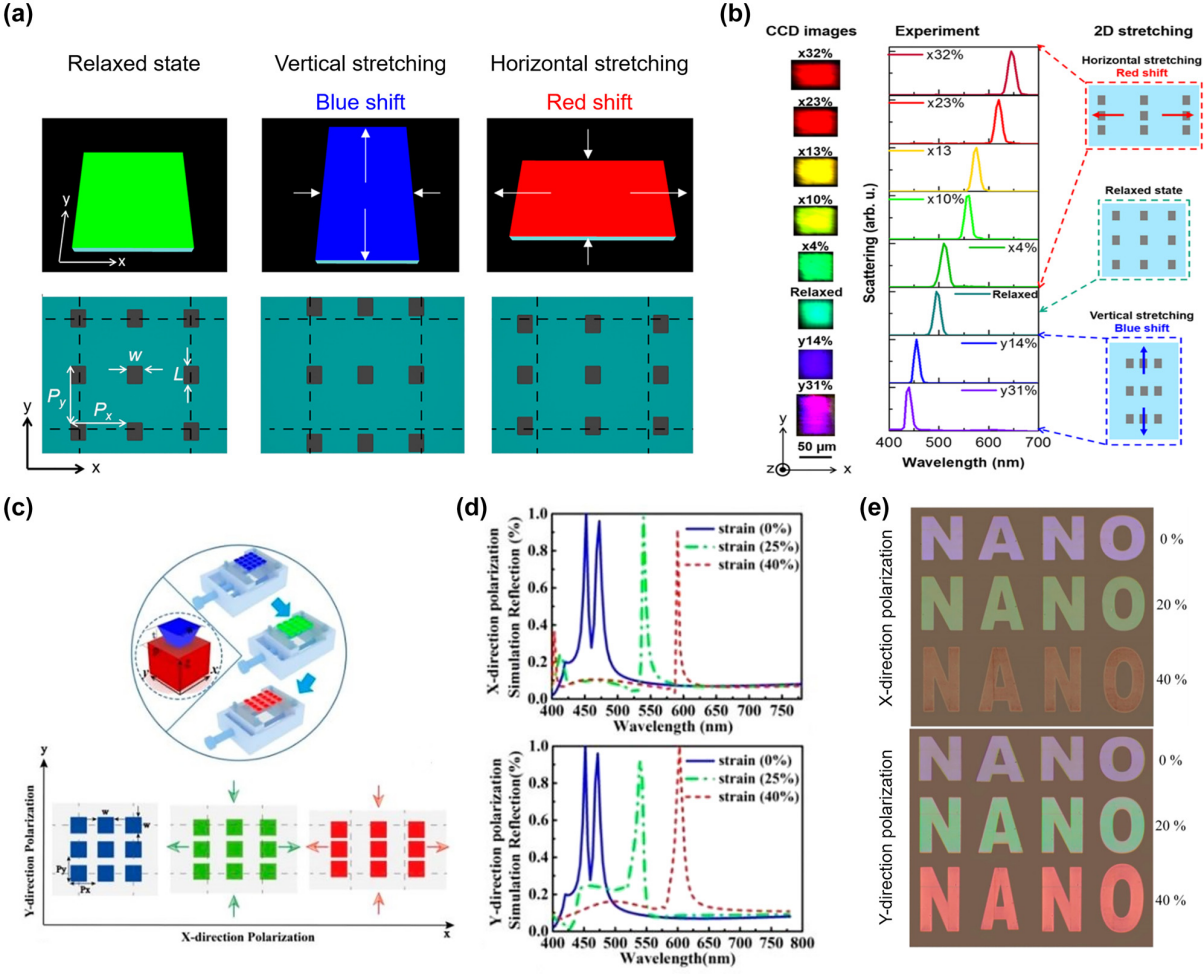
Dynamic structural color generation based on nanostructure geometric configuration modulation through mechanical stretching. (a) Working principle of the full-spectrum stretchable plasmonic color device, in which the top panel shows the schematic diagram of the device’s color change under different stretching conditions, and the bottom panel shows the corresponding schematic of the two-dimensional nanoparticle array. (b) Experimental results of the device performance under different stretching conditions, including the dark-field CCD images (left column) and the reflection spectra (middle column). The right column shows the schematic of the two-dimensional stretching method for full-color tuning. Modified with permission [323]. Copyright 2017, American Chemical Society. (c) Schematic illustration of the stretchable TiO_2_ metasurface and the colors of the device under different strains along the *x*-axis. (d) Reflection spectra of the TiO_2_ metasurface with 0% (solid line), 25% (dotted line), and 40% (dashed line) strains under *x*- and *y*-polarized illuminations. (e) Optical images of a fabricated sample at different stretching conditions under *x*- and *y*-polarized illuminations. Modified with permission [324]. Copyright 2019, American Chemical Society.

Another option for tunability is to alter the geometric configuration of subwavelength nanostructures [112, 323], [324], [325], [326], [327]. Physically stretching and bending the entire device fabricated on a flexible substrate is one frequently used method. Manipulating the flexible substrate can reform the shape of the nanostructures, which then modulates their resonant wavelengths. Figure 32(a) illustrates one design example composed of arrays of 2D Al square on an elastomeric substrate (PDMS) [323]. The device exhibits green color in its relaxed state. When stretching the substrate along the *y*-axis, the lateral interparticle spacing along the *x*-axis decreases which leads to a blueshift of the device’s resonant wavelength; when stretching along the *x*-axis, a redshift in the resonant wavelength occurs. Figure 32(b) shows the experimental results with dark-field CCD-captured images under different stretching conditions. Herein, a cyan color appears in the relaxed state, and then gradually turns to green, yellow, and red as the strain increases along the *x*-axis. Meanwhile, the cyan color changes to blue and then purple when the device is stretched along the *y*-axis.

In general, stretching the flexible substrate along one direction yields compression in the other direction, which breaks symmetry in the device and thus renders the generated color polarization-sensitive. To mitigate such issue, another design composed of arrays of square TiO_2_ nanoblocks and an elastomeric PDMS substrate is proposed (Figure 32(c)), in which the cross section of each nanoblock is an inverse trapezoid with a trapezoidal corner around 72° [324]. The designed device displays blue color in its relaxed state (0% strain), supporting electric dipole (ED) and magnetic dipole (MD) resonances simultaneously (Figure 32(d)). The two resonances exhibit different wavelength shift mechanisms, where the ED resonance under *y*-polarization illumination and the MD resonance under *x*-polarization illumination move to longer wavelengths as the strain increases along the *x*-direction, while the ED under *x*-polarization illumination and the MD under *y*-polarization illumination shift to the shorter wavelengths and then disappear in the visible range. As a result, the proposed design exhibits similar dynamic colors for the above two polarization states when the device is mechanically stretched (Figure 32(e)).

**Figure 33:**
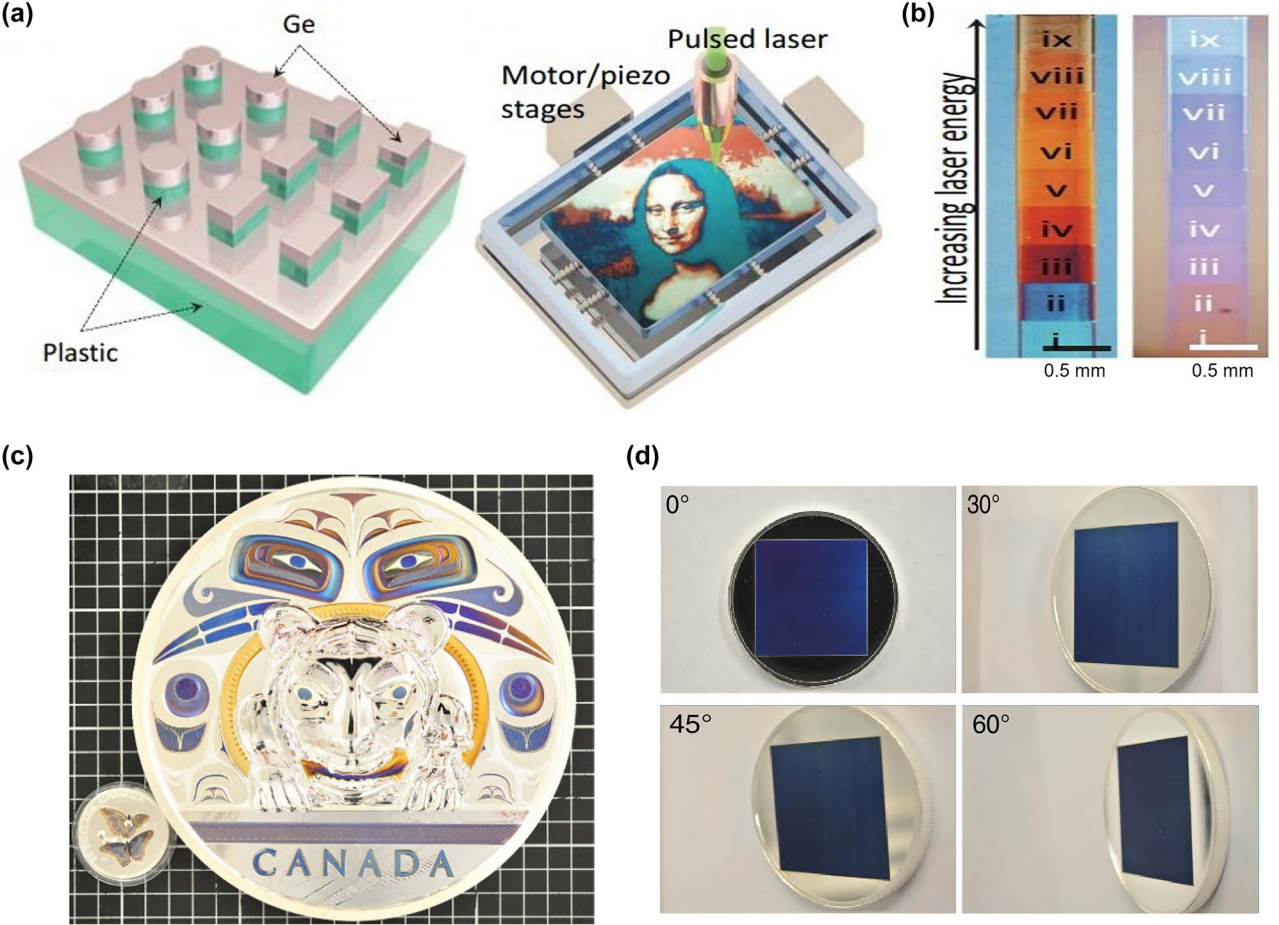
Dynamic structural color generation based on nanostructure geometric configuration modulation through laser post-writing. (a) Schematic illustrations of the Ge-based metasurface (left panel) and the setup of resonant laser printing (right panel). (b) Reflection-mode (left panel) and transmission-mode (right panel) microscope images of the fabricated structures (i to ix) by gradually increasing the laser power from 0.2 to 1.8 nJ with a step size of 0.2 nJ. Modified with permission [330]. Copyright 2017, American Association for the Advancement of Science. (c) Photo of a laser-colored 5 kg Ag coin. The diameter of the coin is 21 cm, and the thickness is 2.5 cm. Left corner shows the photo of an Ag coin (having topography variations of up to 2 mm in height) with laser-colored butterfly patterns. (d) Photos of a blue colored coin taken at four different viewing angles. Modified with permission [333]. Copyright 2017, Nature Publishing Group.

Laser post-writing can reshape the nanostructures and provide an efficient approach for dynamic structural color generation [328–332]. Based on the laser pulse energy density, various surface morphologies that support different resonances are created, leading to an array of structural colors. Zhu and co-workers develop metal-based dynamic structural colors by utilizing the laser post-writing technique [329]. The proposed device is composed of Al disks on top of dielectric pillars (made of either PMMA (polymethyl-methacrylate) or Ormocomp (Micro resist technology GmbH)), hovering above a perforated Al film. When a nanosecond laser pulse is applied, the Al disks are transformed into thicker disks or spheres and finally ablated away as the laser dosage increases. The resonant wavelength blue-shifts from 600 nm to 500 nm and the printed color changes from cyan to yellow as the laser dosage increases from 8 nJ to 535 nJ. In addition to metals, laser post-writing can also be applied to all-dielectric nanostructures. For instance, Zhu and co-workers design Ge-based dynamic structural colors by employing the laser post-writing technique [330]. Figure 33(a) illustrates the schematic representations of the proposed device with Ge deposited on top of the plastic nanopillars and substrate (left panel), and the setup for resonant laser printing (right panel). The strong on-resonance absorption in Ge under pulsed laser irradiation locally elevates the lattice temperature in an ultrashort time scale, leading to the melting and reshaping of nanostructures. The surface-energy-driven morphology changes accordingly, yielding a different color appearance. Figure 33(b) displays the reflection (left panel) and transmission (right panel) microscope images of fabricated samples, which exhibit a gradual color change from cyan to yellow as the applied laser irradiation increases from 0.2 to 1.8 μJ with a step of 0.2 μJ.

Guay and co-workers propose another approach for dynamic structural color generation, where a picosecond laser can produce a full palette of non-iridescent colors (Figure 33(d)) [333]. Moreover, the proposed coloring process can be applied to surfaces of varying quality and morphology, allowing a uniform coloring over rough frosted surfaces and large-area surfaces having significant topographic variations. The produced colors are mainly determined by the total accumulated laser fluence, enabling the fabrication process suitable for high-throughput industrial applications. Photo of a laser-colored 5 kg Ag coin is displayed in Figure 33(c). Left corner shows the photo of an Ag coin with laser-colored butterfly patterns.

Dynamic structural colors can also be produced by employing an external magnetic field [334–337]. For example, Kim and co-workers demonstrate a material called “M-Ink”, whose color is magnetically tunable [336]. The ‘M-Ink’ is composed of superparamagnetic colloidal nanocrystal clusters (CNCs), ethanol solvation layer, and photocurable resin, in which each CNC consists of many single-domain magnetite nanocrystals and is capped with a SiO_2_ shell. Without an external magnetic field ( $\vec{B}=0$ ), the CNCs are randomly dispersed in the liquid resin and exhibit a brown color. Under an external magnetic field ( $\vec{B}={\vec{B}}_{\text{ext}}$ ), the CNCs are assembled to form chain-like structures along the magnetic field lines, where the interparticle distance (d) determines M-Ink’s color appearance. Consequently, the colors can be tuned by adjusting the external magnetic field.

**Figure 34:**
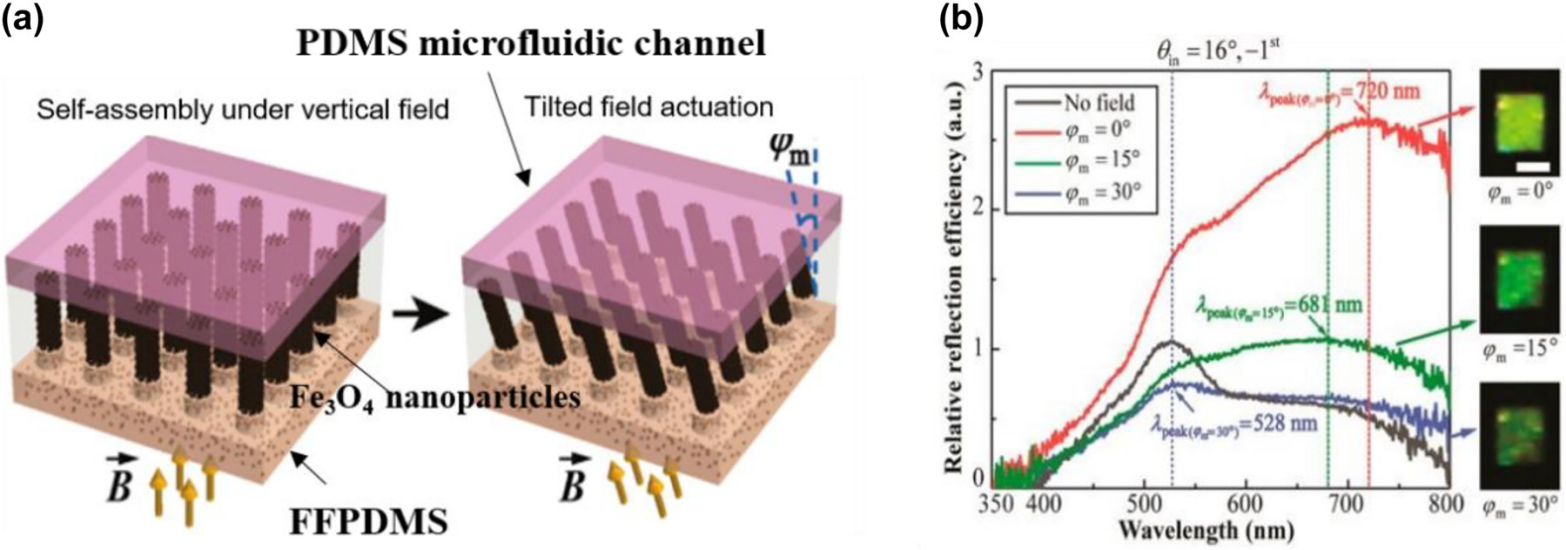
Dynamic structural color generation based on nanostructure geometric configuration modulation through external magnetic field. (a) Schematic illustration of the magnetic field responsive device, which is composed of an FFPDMS template, Fe_3_O_4_ nanoparticles, and a PDMS microfluidic channel. Under an external magnetic field, the FFPDMS template generates a periodic local field to guide the Fe_3_O_4_ nanoparticles into periodic self-assembled columns (SACs). The generated local field also has an “anchor effect” and will immobilize the base of SACs, allowing them to be tilted about the base to induce color change. (b) Measured reflection spectra of a fabricated sample’s -1st order reflection diffraction as well as the associated color appearance under an oblique illumination (*θ*_in_ = 16°). The magnetic alignment angle *φ*_*m*_ varies from 0° to 30°. Scale bar: 1 mm. Modified with permission [337]. Copyright 2019, American Chemical Society.

In spite of changing the interparticle distance, dynamic structural colors can also be produced by tuning the tilt angle of self-assembly nanoparticles. For example, Luo and co-workers introduce a strategy for dynamic iridescence by modifying the orientation of nanostructures via an external magnetic field [337]. The proposed approach employs a 2D periodic magnetic nanopillar array (ferrofluid polydimethylsiloxane, FFPDMS) as a template to guide the assembly of iron oxide (Fe_3_O_4_) nanoparticles in a liquid environment. The FFPDMS is composed of Fe_3_O_4_ nanoparticles (with diameter of 7–10 nm) as well as copolymer of aminopropylmethylsiloxane (APMS) and dimethylsiloxane (DMS). Under an external magnetic field, the FFPDMS generates a periodic local field to guide the Fe_3_O_4_ nanoparticles into periodic self-assembled columns (SACs). These columns are tilted (with a tilting angle *φ*_*m*_) as the direction of external magnetic field varies, leading to a dynamic structural color generation (Figure 34(a)). Figure 34(b) shows the measured reflection spectra (-1st order) under an oblique illumination (*θ*_in_ = 16°). The peak wavelength shifts from 720 nm to 528 nm as the magnetic alignment angle *φ*_*m*_ varies from 0° to 30°, and the associated color changes from bright yellow to dark green.

## 7 Applications

**Figure 35:**
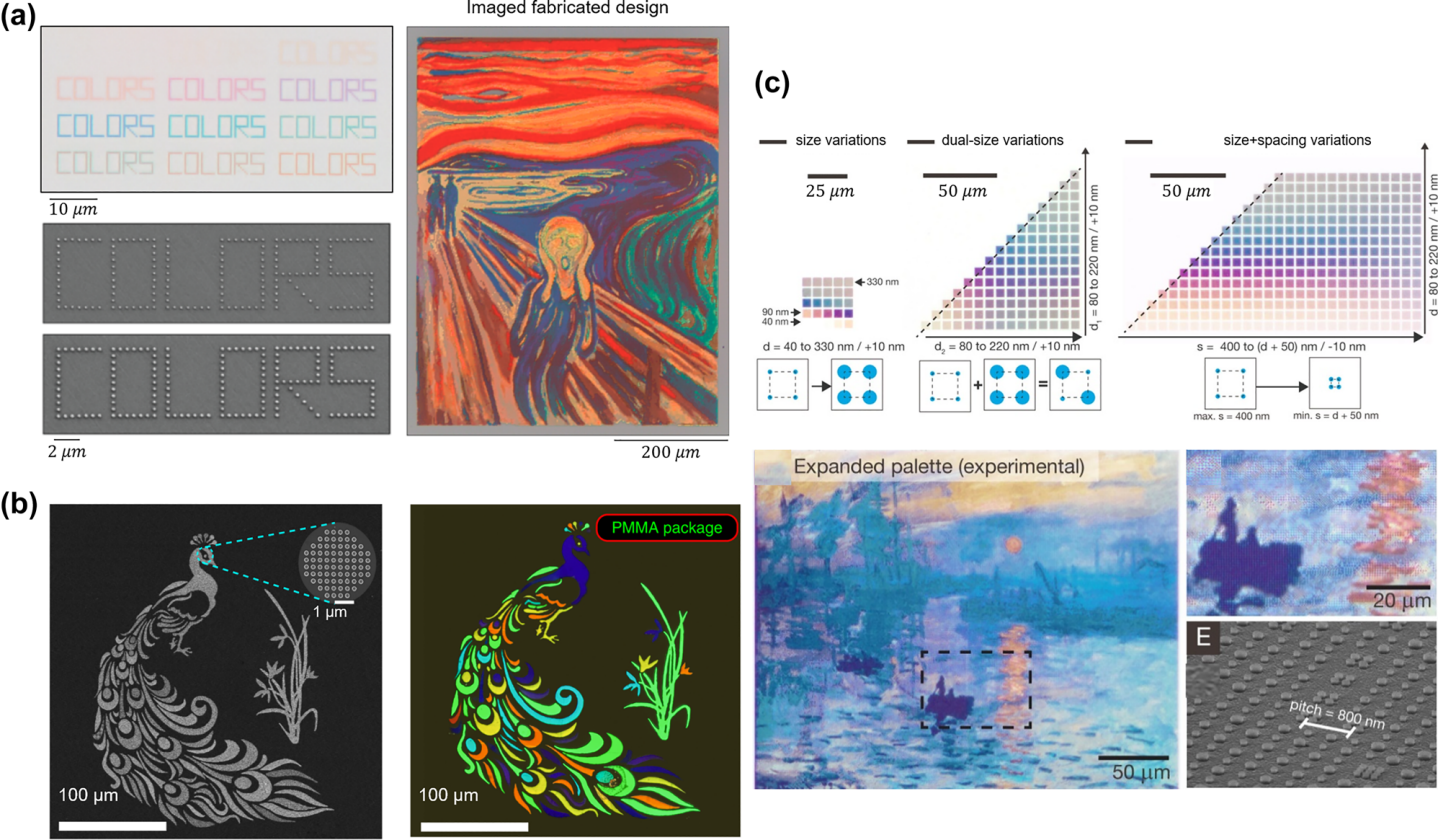
Structural color printing with high purity and large gamut coverage. (a) Si-based structural colors. Left panel: Micrograph of the word “COLORS” printed with single Si nanodisks of different diameters, and SEM images of two “COLORS” patterns. Right panel: Optical micrograph of the painting “The Scream” reproduced by the Si-based structural colors. Modified with permission [340]. Copyright 2021, American Chemical Society. (b) SEM image of the sample “peacock with an orchid” and bright-field microscope image of the sample packaged with a PMMA index matching layer. Modified with permission [166]. Copyright 2020, Nature Publishing Group. (c) Upper panel: color mixing strategy for Al color pixels, where the basic color pixel is formed by four identical nanodisks; Lower panel: reproduction of Monet’s “Impression, Sunrise” using the expanded palette of Al color pixels and an associated SEM image of the sample. Modified with permission [341]. Copyright 2014, American Chemical Society.

**Color printing**. Over recent years, structural color printing has been exploited as a promising alternative to conventional color printing that is based on natural or synthetic chemical pigments [338, 339]. Colors generated by nanoscale structures have several advantages including improved lifetime and fine spatial resolution. In addition, these colors can be readily tuned by adjusting the nanostructure’s constituent materials and geometric parameters, or by applying external stimuli. Researchers have made numerous efforts to improve the performance of structural colors in terms of purity, brightness, and gamut coverage. Nanostructures based on low-loss dielectric materials can support an array of electrical and magnetic resonances, and thus provide a promising platform for generating colors with finely-tuned hue, high saturation, as well as broad gamut coverage. Flauraud and co-workers design an all-dielectric metasurface consisting of Si nanodisk resonators for nanoscale color printing [340]. Through variation of the nanodisk’s diameter and spacing, the generated colors span a broad color palette with a continuous change in hue and saturation (Figure 35(a)). Yang and co-workers further enlarge the gamut area of Si-based metasurfaces to 181.8% of sRGB with the assistance of refractive index matching layer [166]. The reduced refractive index contrast between the substrate and matching layer significantly suppresses undesired background reflection and narrows the Si meta-atom’s resonant linewidth, leading to higher color brightness and purity. Employing a group of size-varying Si resonators embedded in an index matching layer, researchers demonstrate a hundred-micron-scale peacock pattern with vivid color and near perfect black background (Figure 35(b)).

Traditional pigment-based printing attains a full-color spectrum by combining primary colors with different proportions. Inspired by this well-developed color toning technique, Tan and co-workers demonstrate a mixing strategy for plasmonic-resonator-based structural colors (Figure 35(c)) [341]. In the proposed design, each plasmonic color pixel consists of four individual Al nano-resonators with two different in-plane diameters. The resonators located along the pixel’s diagonal direction have an equal in-plane diameter of value *d*_1_, while the other two identical resonators located along the off-diagonal direction have a different in-plane diameter of value *d*_2_. Creation of various colors is accomplished by properly positioning different-sized resonators within each pixel, and subtle tuning of the color hue and saturation is further realized by adjusting the spacing between resonators. Using this strategy, the researchers expand the range of printable colors from ∼15 basic colors to over 300 ones. The extended structural color palette is used to print Claude Monet’s painting “Impression, Sunrise”. Such “plasmonic replication” retains fine details of the original painting, showing fidelity down to its brush strokes characteristic.

**Figure 36:**
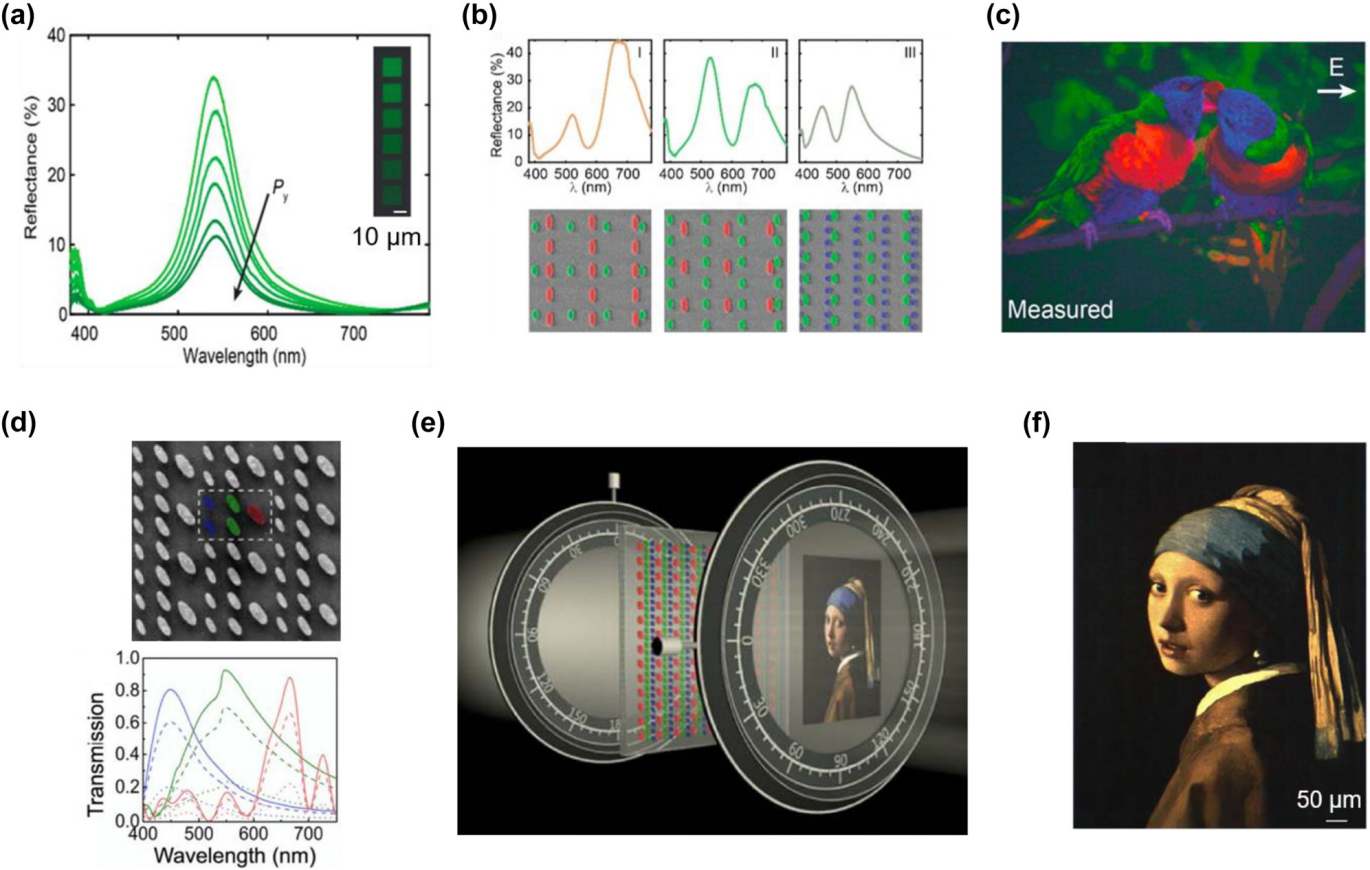
Structural color printing with pixels of tunable brightness. (a) Measured reflectance spectra of green-color Ag nanorod pixel arrays, where the luminance decreases with the increase of lattice spacing along the rod’s long axis (*P*_*y*_). Insets: associated bright-field microscope images of different samples. (b) Measured reflection spectra (upper panel) and corresponding false-colored SEM images (lower panel) of the mixed nanorod arrays. (c) Optical bright-field image of a fabricated sample showing two colorful parrots. The sample consists of nanorod arrays with tunable brightness and chromaticity. The illumination light is linearly polarized along the rod’s long axis (as the arrow indicates). The fabricated sample is 240 × 312 μm in size, and its square pixel has a side length of 1.28 μm. Modified with permission [342]. Copyright 2020, American Chemical Society. (d) Upper panel: SEM image of a fabricated sample consisting of TiO_2_ nanopillars. A super-cell is enclosed by white dashed lines and the false coloring of nanopillars indicates the primary colors that are generated by the respective nanopillars. Scale bar: 500 nm. Lower panel: Calculated transmission spectra corresponding to red (red curves), green (green curves), and blue (blue curves) colors when the nanopillar orientation angle varies from 45° (solid lines) to 30° (dashed lines) and 15° (dotted lines). (e) Illustration of the experimental setup for full-color printing, where a linear polarizer is placed behind the metasurface to decode the generated colors. (f) Reproduction of the painting “girl with a pearl earring”. Scale bar: 50 μm. Modified with permission [244]. Copyright 2020, Optica Publishing Group.

For many practical applications, a gradual and continuous variation in color brightness helps to illustrate variant contrast of light and shade, and at the same time, facilitates the perception of three-dimensional scenes. However, a majority of structural color designs focus on obtaining high reflection (or transmission) intensities in the generated spectra while are incapable of achieving well-controlled variation in color brightness. To mitigate such issue, Hail and co-workers demonstrate a plasmonic metasurface consisting of anisotropic Ag nanorods to attain independent and fine control over both the chromaticity and luminance of generated colors [342]. When an array of identical nanorods is illuminated by incident light polarized along the rod’s long axis, adjusting the lattice spacing along this direction leads to a notable change in the generated color’s luminance, and simultaneously, a negligible variation in its chromaticity (Figure 36(a)). In addition, arranging different nanorod arrays which respectively generate RGB colors along the direction orthogonal to their long axes enables delicate manipulation over the color’s chromaticity without obviously affecting its luminance (Figure 36(b)). The aforementioned method is employed to reproduce a photograph of two colorful parrots (Figure 36(c)), which exhibits rich details of transitions in color chromaticity and brightness. Another design presented by Huo and co-workers realizes full-color and brightness-tunable metasurfaces for photorealistic nano-painting, which consists of different half-wave-plate nanopillars with spatially-varying rotation angles [244]. By adjusting the in-plane dimensions of TiO_2_ nanopillars, three types of half-wave plates that, respectively, correspond to the primary RGB colors are obtained. In addition, the rotation angle of each nanopillar is chosen based on the targeted color brightness at its location (Figure 36(d)). When a linearly polarized white light illuminates the metasurface, the output beam gets spatially modulated both in its spectrum and state of polarization. After the output beam further passes through a linear polarizer (Figure 36(e)), its spatially-varying states of polarization lead to different spectrum intensities based on the Malus’ Law, enabling a flexible and fine manipulation over the brightness level of generated RGB colors. A fabricated sample which reproduces “girl with a pearl earring” (Figure 36(f)) exhibits a smooth and subtle transition in color brightness, mimicking the photorealistic texture of the original oil painting.

**Figure 37:**
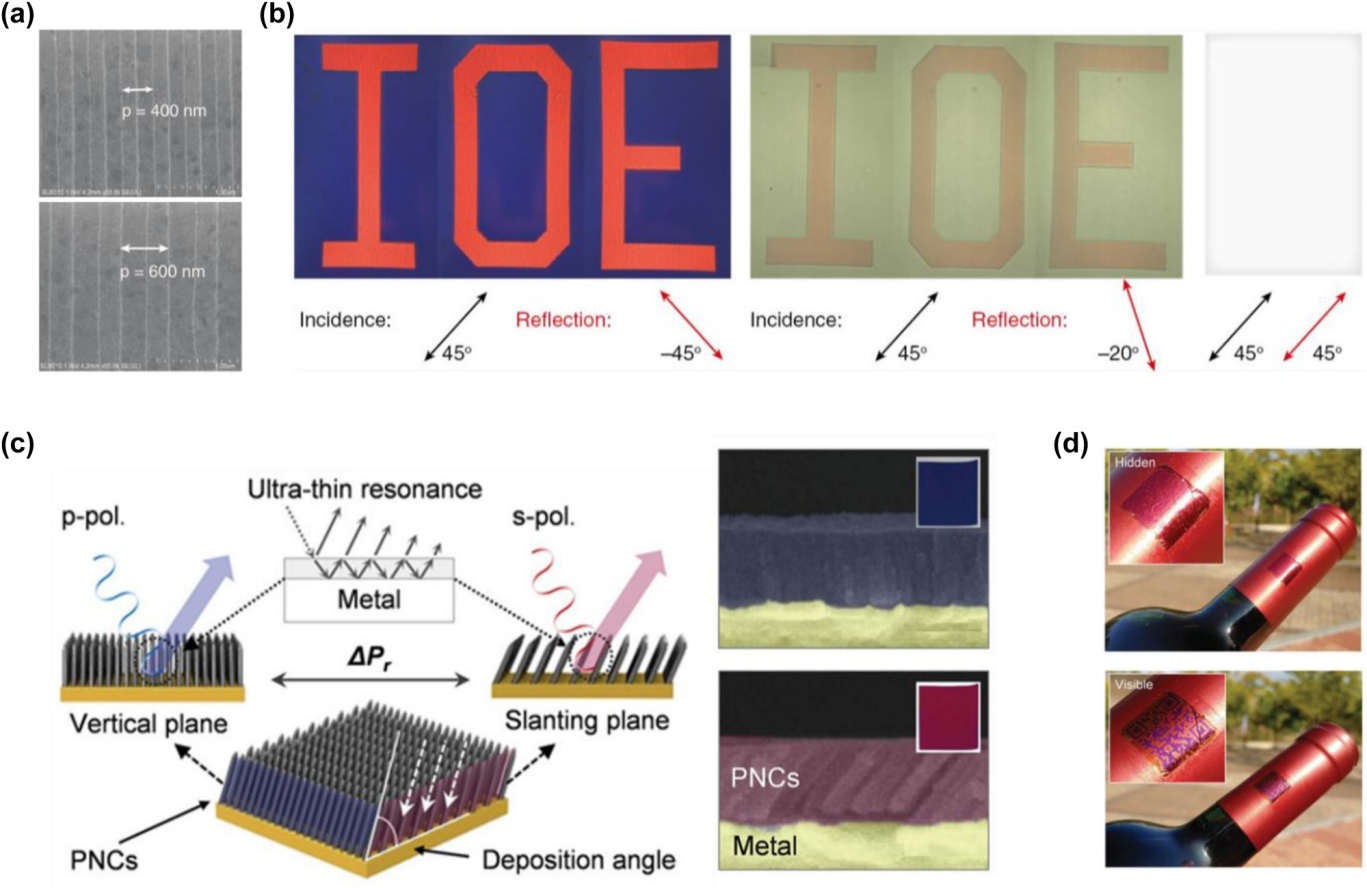
Dual-color encryption based on polarization-sensitive structural coloration. (a) SEM images of the plasmonic shallow grating structures. (b) Optical micrographs of the sample showing letters “IOE”, where the polarization direction of the linearly incident light is set to 45° and that of the reflection light to −45°, −20°, and 45°, respectively. Modified with permission [133]. Copyright 2018, Walter de Gruyter GmbH. (c) Schematic of the slated lossy nanocolumns viewed from different in-plane orientations. When viewed from the vertical plane, the nanocolumn array shows a bluish color; while viewed from the slanting plane, the array shows a purplish color. (d) Photos of the QR code metasurface placed over a curved bottleneck surface contrasting with and without encryption in an outdoor environment. Modified with permission [343]. Copyright 2020, Wiley-VCH.

**Information encryption**. Many practical applications, such as QR codes, ID cards, and anti-counterfeit labels, require advanced encryption technologies to encode certain information in the printed images or patterns. Polarization-sensitive structural coloration, which displays different colors based on different states of polarization of the illumination light, provides a viable solution to the aforementioned requirement. Song and co-workers propose a dual-color encryption metasurface device consisting of plasmonic shallow gratings [133]. As the 1D nanograting structure (Figure 37(a)) possesses anisotropic EM responses, its generated color varies for different polarization components of the reflection light. As depicted in Figure 37(b), the fabricated sample of letters “IOE” exhibits high contrast colors (blue and red) for the reflected light whose state of polarization is set perpendicular to that of the incident light. As the polarization state of reflected light gradually varies towards that of the incident light, the color patterns fade into the white background accordingly. Another dual-color encryption metasurface which is made of slated nanocolumns is further applied to the curved and wrinkled surfaces of daily objects [343]. As shown in Figure 37(c), the lossy nanocolumn array with a specific slanting orientation possesses different effective refractive indices for the s- and p-polarized illumination. As the polarization of incident light is rotated, resonant condition of the metasurface changes and the generated color varies accordingly. For a QR code sample, when the polarization direction of illumination light is set to a predefined angle, the QR code’s fine details can be clearly distinguished from the background. In contrast, when the polarization angle of illumination light is not properly set, the QR code pattern gets dimmed with its background (Figure 37(d)).

**Figure 38:**
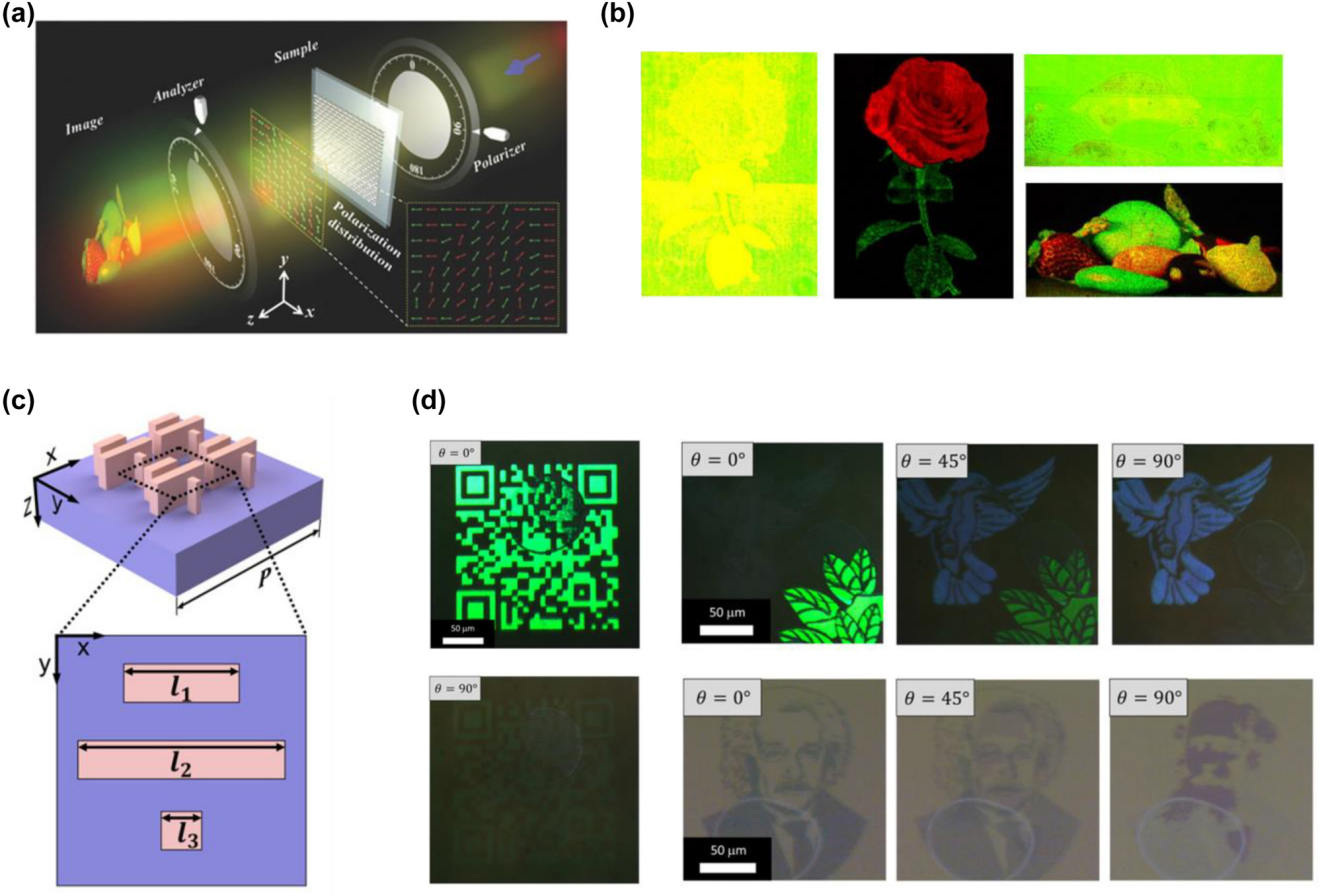
Full-color encryption based on polarization-sensitive structural coloration. (a) Schematic of the polarization encryption setup. A linear polarizer in front of the metasurface generates a linearly polarized light, and the metasurface consisting of miniature half-wave plates can modulate the linearly polarized incident light into a transmitted light with a collection of spatially-varying polarization states. The output linear polarizer (analyzer) with its transmission axis perpendicular to that of the input polarizer transforms the light beam from the metasurface into a color image. (b) Color images by means of polarization encryption without (the left and the upper right images) and with (the middle and the lower right images) the required analyzer. Modified with permission [344]. Copyright 2018, Wiley-VCH. (c) Schematic of the resonant unit consisting of a triple-nanofin structure of the same height, width and separation, but of varying lengths. (d) Left panel: micrographs of a QR code. Right panel: dual-color images of a blue bird on green leaves (upper row) and overlapped dual-portrait images (lower row). The appearance of these images changes as the polarization angle (*θ*) of incident light rotates. Modified with permission [345]. Copyright 2021, Walter de Gruyter GmbH.

Dual-color encryption metasurface is an efficient approach to encrypt single-colored subject. However, to enhance the density of encrypted information, the approach to encoding more complicate images composed of diverse colors needs further investigation. Zang and co-workers demonstrate a dielectric metasurface capable of simultaneously encoding the color chromaticity and brightness into various states of polarization at a subwavelength pixel scale [344]. The meta-atoms of the metasurface are designed to function as wavelength-dependent ultra-compact half wave-plates, which will rotate the polarization direction of a linearly polarized incident beam. Through an array of resonators with different response wavelengths and orientations at each location (Figure 38(a)), the metasurface can modulate a linearly polarized incident beam into a transmitted vector beam which possesses various color responses and polarization directions at different locations over its transverse plane. Behind the metasurface, a linear polarizer is placed to decode the spatially distributed brightness information in the modulated light. As shown in Figure 38(b), the directly transmitted light through the metasurface only features a weak profile of the encoded color pattern. Yet with the aid of a polarizer, the transmitted light finally reveals as an expected color image.

Another full-color, polarization-sensitive metasurface design proposed by Jung and co-workers is further modified to sufficiently diminish the residual color information by the cryptographic mode [345]. The resonant units of the dielectric metasurface are designed as triple-nanofin structures (Figure 38(c)). In response to the geometric parameters of three meta-atoms within one unit, Mie resonances are initiated to produce red, green, or blue colors when the polarization direction of incident light is parallel to the long axis of nanostructures, while are largely suppressed to shut down color display if the polarization direction is rotated to a perpendicular state. The proposed design is applied to the image transitions in response to the rotated polarization direction of incident light. The experimental result indicates approximately dimmed color information at the cryptographic mode (Figure 38(d)).

**Figure 39:**
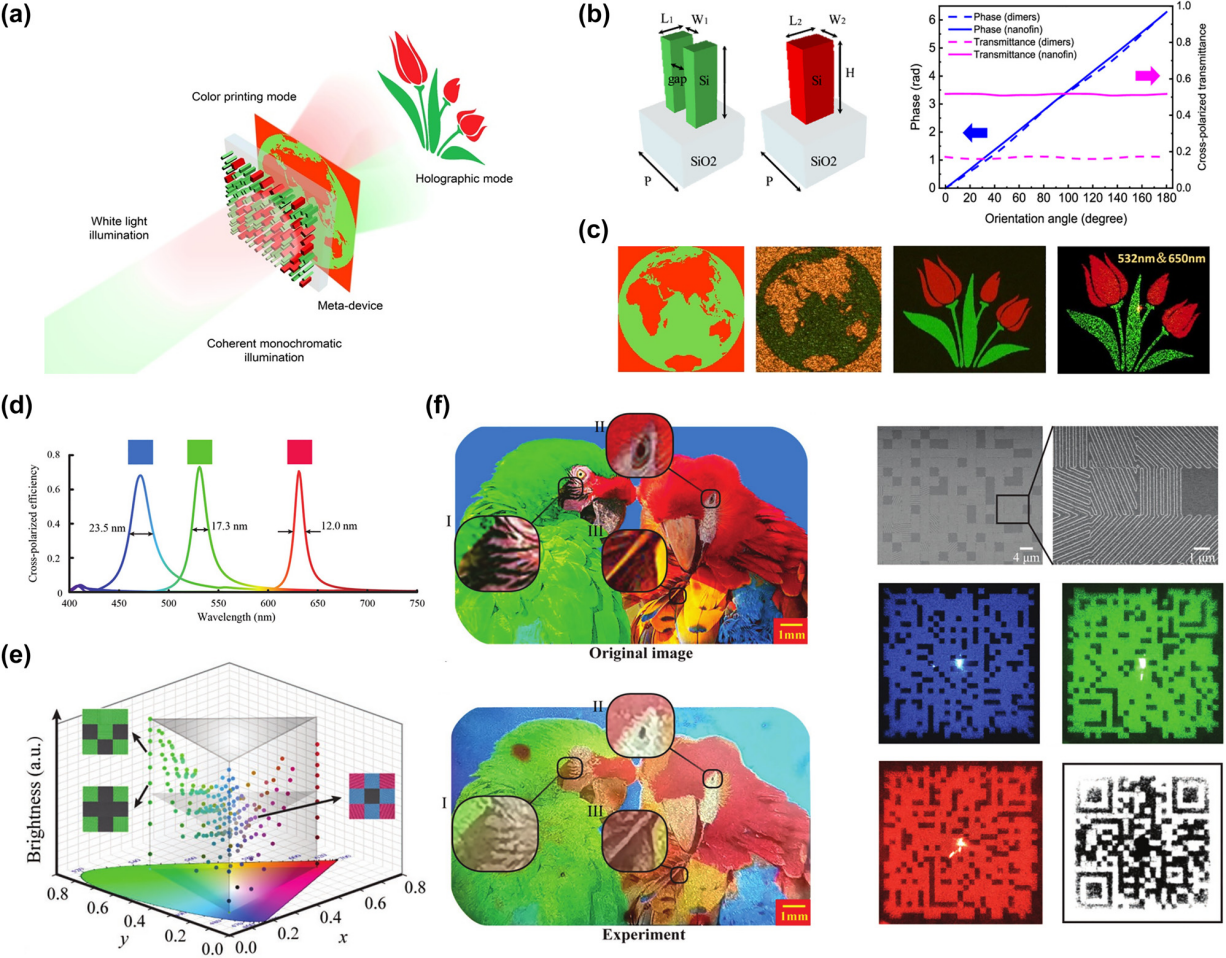
Dual-functional metasurface integrating color printing and hologram projection. (a) Schematic of the meta-device displaying both color printing and hologram projection. (b) Left panel: Schematic diagram of the Si nanodimer and nanofin structures on glass substrate. Right panel: phase responses and cross-polarized transmittances of these two meta-atoms at different orientation angles. (c) Design and experimental results of the dual-functional device. The clear bicolor pattern of the “earth map” results from the color printing mode, and the “red blossoms” and “green leaves” images result from the hologram projection mode. Modified with permission [350]. Copyright 2019, American Chemical Society. (d) Simulated cross-polarized spectra of the plasmonic shallow grating (PSG) units, which, respectively, display red, green, and blue colors. (e) Schematic diagram of the color adjusting system. The RGB color units and Ag nanomirror units are arranged into a 3 × 3 array in proper ratios to create colors with tunable chromaticity and brightness. (f) Left column: Results of the full-color printing, including the original image of two macaws (upper panel) and the optical image of a fabricated device (lower panel). Right column: SEM images of a fabricated sample (upper panel), and holographic images by means of blue, green, and red coherent illuminations. The pattern of the complete QR code (bottom right, lower panel) is restored from three measured subparts. Modified with permission [351]. Copyright 2020, Wiley-VCH.

In addition to polarization-sensitive colored metasurface devices, the integration of color printing and wavelength-multiplexed hologram [346, 347] within single-layered metasurfaces provides a promising prospect on image encryption, and at the same time, further increases the information capacity. Such integration requires an array of well-designed meta-atoms to simultaneously obtain color printing by means of specific intensity responses as well as holographic projection by means of proper phase modulations [348, 349]. Wei and co-workers accomplish concurrent color printing and holography on a bicolor metasurface utilizing amorphous silicon dimers and nanofins as meta-atoms (Figure 39(a)) [350]. The geometric parameters of the nanodimer and nano-fin structures are optimized for green and red colors separately (Figure 39(b)). Meanwhile, a phase modulation ranging from 0–2*π* is accomplished by changing the azimuthal angles of the nanodimer and nano-fin structures following the Pancharatnam–Berry (PB) phase modulation principle with a negligible effect on the spectral intensity. By tuning the spectral and phase responses respectively in all meta-atoms, a dual-functional meta-device is realized, displaying a clear bicolor pattern of the “earth map” (color printing) and holographic images of “red blossoms” and “green leaves” (Figure 39(c)). Another dual-functional metasurface presented by Zhang and co-workers realizes color printing with flexible chromaticity and brightness tuning, and at the same time, a tri-channel holographic projection [351]. Owing to the narrowband resonances generated from the plasmonic shallow grating (PSG) based meta-atoms, additive colors with chromaticity variation are obtained when the PSG units respectively displaying red, green, and blue colors are arranged within the color pixels with a proper ratio (Figure 39(d)). Furthermore, color brightness is adjustable by the introduction of Ag nanomirror structure into different color pixels (Figure 39(e)). The phase profile for tri-channel holography is implemented through rotating the tricolor PSG orientations following the PB principle. As such, a sample with two colorful macaws (color printing) and a QR code (tri-channel hologram) showing three subparts respectively is successfully implemented (Figure 39(f)).

**Colorimetric sensors.** Structural color devices can serve as nanoscale sensors via measuring the color variation due to external stimuli. The color exhibited by a metasurface-based sensor is sensitive to the imperceptible changes of environmental refractive index and its own geometric parameters. Consequently, metasurface-based colorimetric sensors can be roughly categorized into two types: colorimetric refractive index variation sensors [352–354] and colorimetric geometric variation sensors [355, 356], where the first type refers to sensors that work by measuring the color change resulting from variation in refractive index of the surrounding media, and the second type refers to sensors (constructed of tunable materials) that work by measuring the color change when external stimuli affect the material’s dimensions.

**Figure 40:**
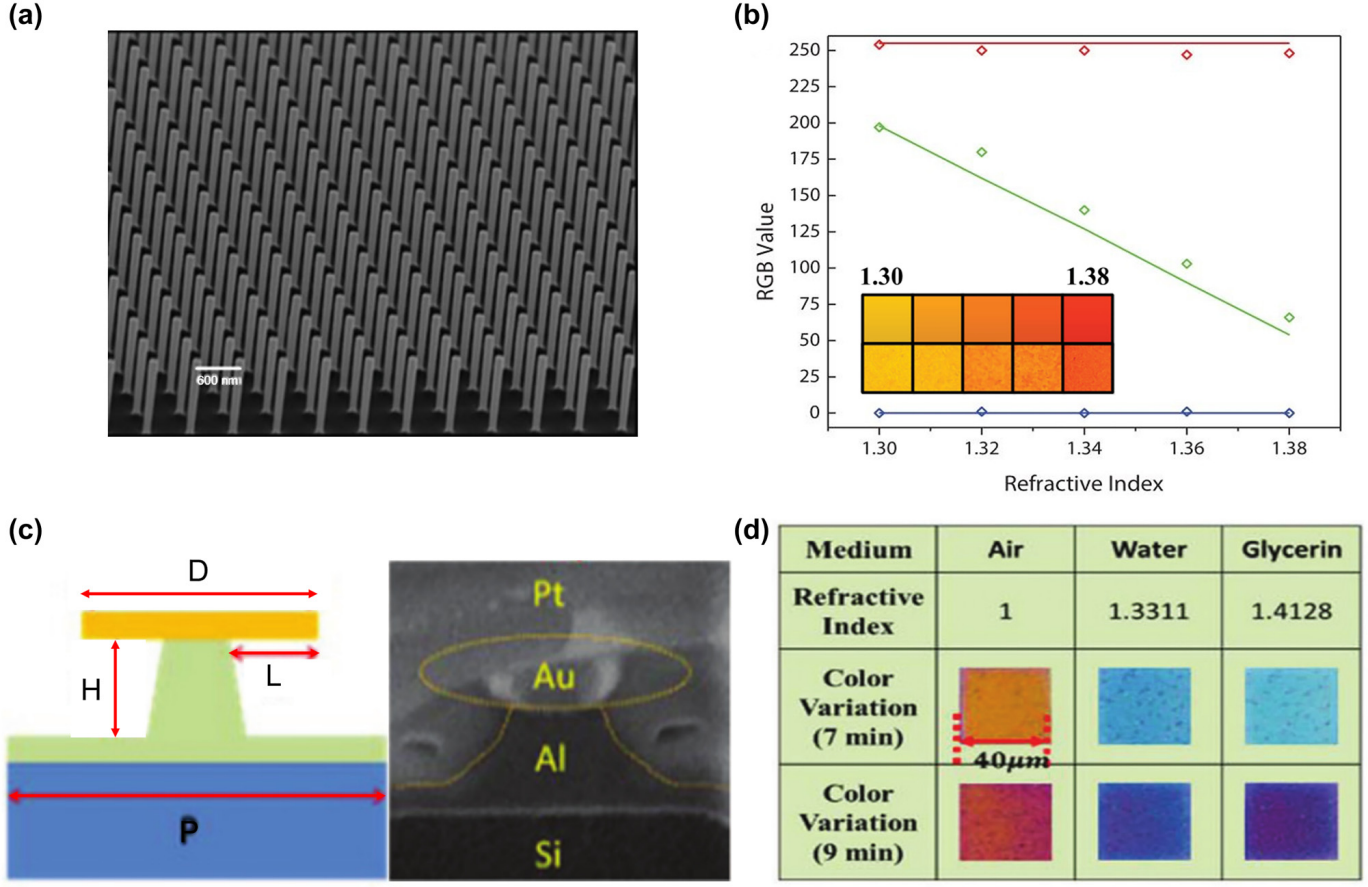
Colorimetric refractive index sensors. (a) SEM image of the Si nanowire array. (b) Experimentally measured (open diamonds) and theoretically predicted (solid lines) results of the displayed colors’ RGB values varying with the surrounding refractive index. Insets: the associated theoretically predicted (upper panel) and experimentally observed (lower panel) colors. Modified with permission [352]. Copyright 2014, Wiley-VCH. (c) Schematic (left panel) and SEM image (right panel) of the three-layer stacked structure consisting of an Au disk, an Al pillar, and a planar Al reflector. (d) Colorimetric responses of two sensors in air, water, and glycerin (60%), respectively. The color of the sensor with a 7 min etching time (the cavities have not formed yet) transits from yellow to blue and then light blue for different surrounding media, while the color change of the sensor with a 9 min etching time (the cavities have formed) is more noticeable (red–blue–violet). Modified with permission [353]. Copyright 2020, Royal Society of Chemistry.

To study the colorimetric refractive index variation sensors, Walia and co-workers demonstrate a metasurface device in the form of vertically arranged Si nanowires (Figure 40(a)) [352]. The diffracted wavelength of the nanowire array (which can be regarded as a 2D grating) possesses a linear shift to the change of surrounding refractive index, leading to a predictable color transformation corresponding to the variation of environmental refractive index. As the refractive index varies, the device exhibits naked-eye distinguishable color changes (Figure 40(b)). Zhu and co-workers propose another colorimetric refractive index variation sensor, which consists of Au disks atop Al pillars on a planar Al reflector (Figure 40(c)) [353]. The vertical air cavities between the top Au disks and bottom Al reflector can confine the light field to a deep subwavelength scale and facilitate light–matter interaction, leading to the device’s significant color variation as its surrounding refraction index changes. An array of experiments to present the device as a refractive index sensor are shown in Figure 40(d), exhibiting colorimetric response to different surrounding refractive indices.

**Figure 41:**
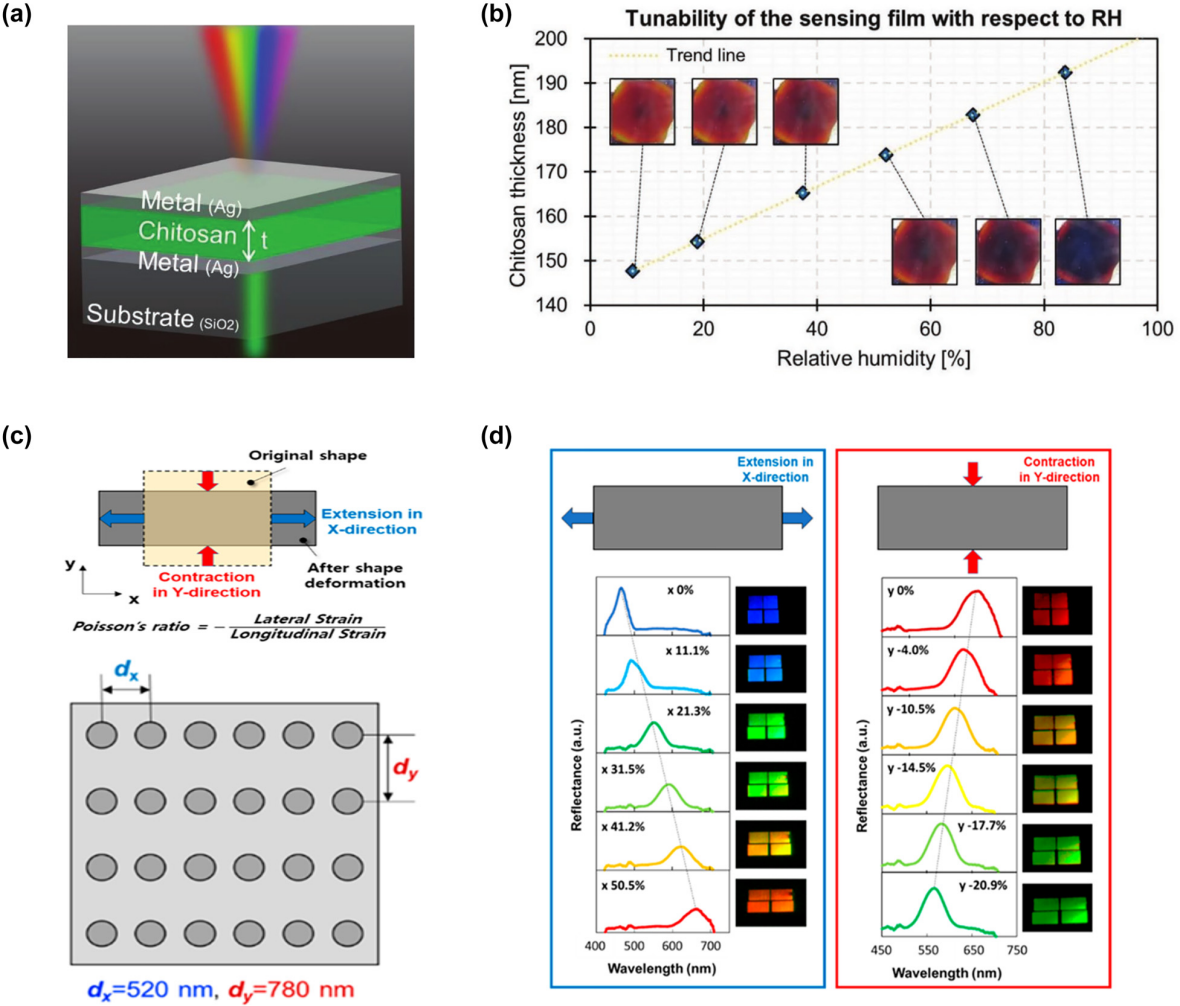
Colorimetric geometric variation sensors. (a) Schematic of the metal–insulator–metal (MIM) structure-based sensor, consisting of two Ag mirrors and a chitosan hydrogel layer in between. (b) Thickness change of the chitosan layer in response to the external relative humidity (RH) variation. Insets: displayed colors of the sensor for different RH values. Reproduced with permission [355]. Copyright 2020, Wiley-VCH. (c) Schematic of the colorimetric strain sensor, whose pitches in the horizontal and vertical directions are asymmetric. (d) Spectrum and color change of the sensor with extension in the horizontal (*X*-) direction and contraction in the vertical (*Y*-) direction. Modified with permission [356]. Copyright 2020, American Chemical Society.

As for the colorimetric geometric variation sensors, Jang and co-workers report a metal–insulator–metal (MIM) structure (Figure 41(a)), where chitosan hydrogel is selected as the insulator whose thickness varies as the relative humidity (RH) changes [355]. A naked-eye distinguishable color shifting appears, accounting for an effective optical thickness change of the chitosan insulator in response to the environmental RH value alteration (Figure 41(b)). Quan and co-workers propose another colorimetric geometric variation sensor consisting of dielectric nanostructures patterned on an elastomeric substrate (Figure 41(c)) [356]. This stretchable, diffractive, color-based wireless sensor can measure strain using the entire visible spectrum, based on an array of cone-shaped Si nanostructures on the surface of PDMS substrate (Figure 41(d)).

**Colored solar cells**. Colored solar cells have attracted wide interest over the past decades due to their potential applications in building integrated photovoltaics to exploit the otherwise wasted solar energy in an aesthetically pleasing manner. By their decorative appearance, the applicable scenarios of colored solar cells have been extended from secluded places, which are usually for conventional solar cells, to the walls, roofs, and other parts of buildings. To obtain a decent photoelectricity performance, decorative colors with low energy absorption, high stability, and adjustable spectrum are introduced into the construction of solar cells.

**Figure 42:**
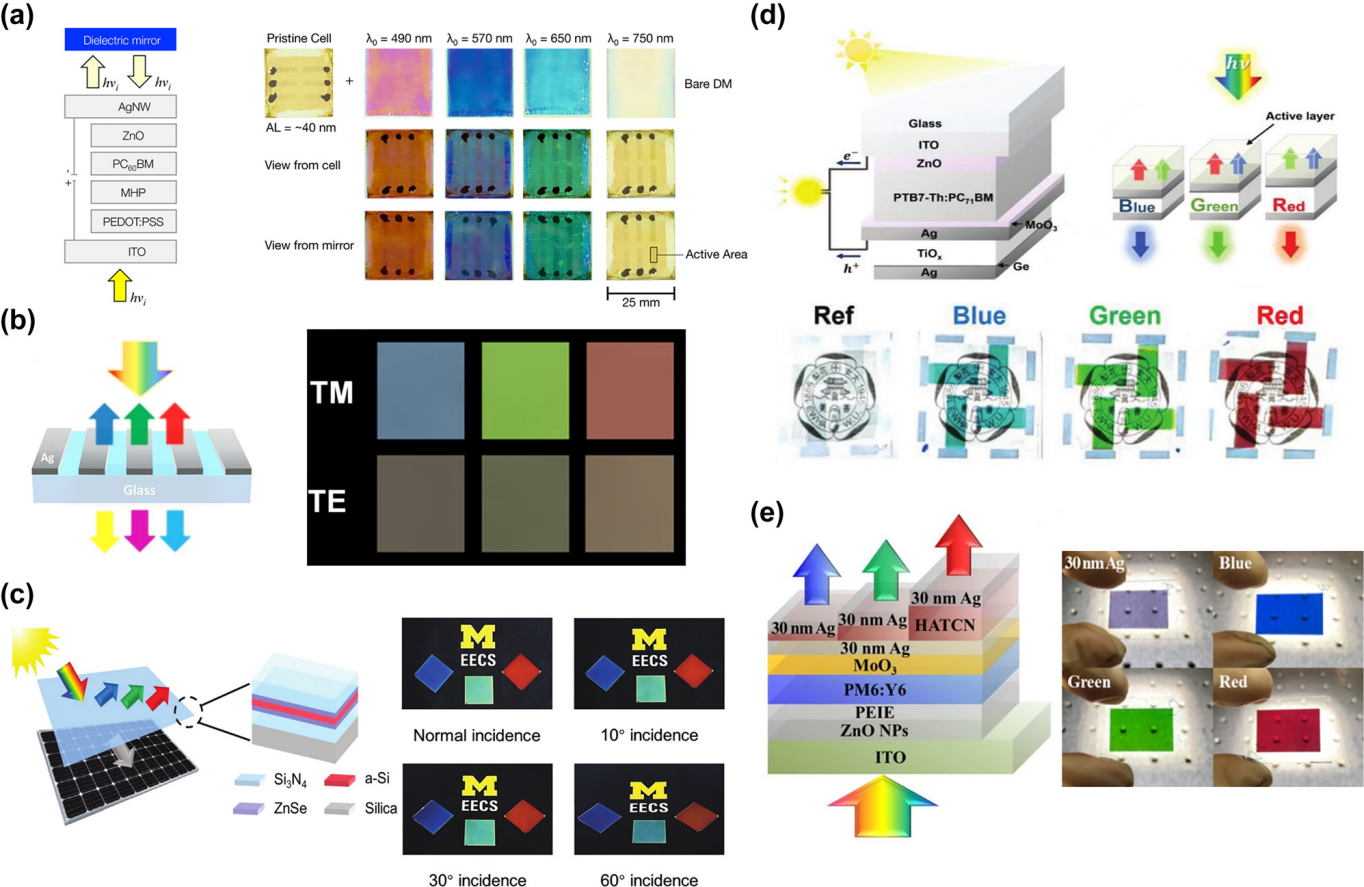
Colored solar cells. (a) Left panel: schematic illustration of the colored solar cell. The colors are generated by the 1D photonic crystal reflective mirror. Right panel: photos of the pristine cell, four bare dielectric mirrors (Bare DMs), and full-devices comprising both the pristine cell and reflective mirrors. Modified with permission [357]. Copyright 2016, American Chemical Society. (b) Left panel: schematic diagram of the plasmonic color filter, consisting of a subwavelength Ag grating layer atop a glass substrate. Right panel: photos of the fabricated color filters showing that the RGB reflection colors are observed only under TM polarized illumination (electric vector perpendicular to the nanograting). Modified with permission [358]. Copyright 2017, Nature Publishing Group. (c) Left panel: schematic of an improved-efficiency colored solar cell with a multilayer filter atop. Right panel: photos of the fabricated RGB filters taken at different viewing angles. Modified with permission [360]. Copyright 2019, Royal Society of Chemistry. (d) Upper panel: schematic of the organic solar cell employing a color filter electrode. Lower panel: photos of the reference transparent solar cell and three colored solar cells respectively having an RGB color filter electrode. Modified with permission [361]. Copyright 2018, Wiley-VCH. (e) Left panel: schematic of the high-efficiency organic solar cells with microcavity electrodes. Right panel: photos of the original 30 nm-thick Ag electrode and the micro-cavity (Ag/HATCN/Ag) based electrodes exhibiting RGB colors. Modified with permission [362]. Copyright 2021, Elsevier BV.

To obtain a desired color and simultaneously enhanced power conversion efficiency, Quiroz and co-workers propose a semitransparent perovskite solar cell with a backside dielectric mirror (Figure 42(a)) [357]. The dielectric mirror, composed of a stack of 10 alternating layers with high and low refractive indices, is designed to satisfy the Bragg condition for generating the desired reflective color. By combining the colored dielectric mirror with the perovskite cell, photovoltaic devices showing distinct colors are realized. Lee and co-workers obtain another colored solar cell by integrating a perovskite solar cell with a plasmonic subwavelength grating atop a transparent substrate (Figure 42(b)) [358]. Vibrant color with a sharp reflection peak is generated based on the localized resonance supported by the ultrathin metallic nanograting structure. The complementary part of the incident spectrum is then transmitted to the underneath perovskite solar cell for solar energy generation. Park and co-workers also show that photonic nanostructures incorporated with organic photovoltaics, where conjugated polymer (light-absorbing) layers are sandwiched between an Au nanograting layer and a continuous thick Al film, are capable of simultaneously producing reflection colors and generating electrical powers [359].

Adding a structural color filter to a solar cell may lead to a significant reduction of the device’s power conversion efficiency. To mitigate such issue, Ji and co-workers refine the filter constitution to reflect a distinctive narrow-band spectrum, producing vivid RGB colors with high peak reflection efficiency (∼60%) and angle-invariant appearance (±60°), and simultaneously, to transmit the remaining broadband light spanning the whole solar spectrum to enhance the device’s power conversion efficiency (Figure 42(c)) [360]. Apart from adding additional layers to alter the colors of photovoltaic devices, modifying the constituent components of solar cells is another approach. For example, Kim and co-workers implement a colored electrode to realize the function of both spectrum selection and energy back-flow towards the solar cell active layer [361]. The colored electrode is composed of a TiO_*x*_ layer switched by two Ag mirrors, where the resonant wavelength is largely determined by the TiO_*x*_ layer thickness. The inner Ag layer interfacing the charge-transporting layer functions as an electrical contact of the solar cell. Upon receiving the full spectrum of normally incident sunlight, the colored electrode selectively transmits the resonant band while reflects the nonresonant band. The active material then harnesses the reflected light, leading to additional charge generation (Figure 42(d)). Sung and co-workers achieve a power conversion efficiency over 13% by utilizing a micro-cavity structured electrode (Figure 42(e)) [362]. An Ag-HATCN-Ag FP cavity based electrode is applied to the fabrication of colored solar cells. Here, HATCN denotes hexaazatriphenylenehexacarbonitrile. Diverse transmission-type colors associated with different HATCN layer thicknesses are generated.

## 8 Summary and outlook

In this review, we summarize some recent developments of structural color generation based on various photonic nanostructures, including layered thin films and optical metasurfaces. We first explain that color is not a physical parameter, but rather a subjective perception of an individual observer through a complex visual process. To connect an object’s visible spectrum to its physiologically perceived color, two popular color spaces, namely, the CIE 1931 *XYZ* color space and the CIE 1976 L^*^a^*^b^*^ color space, are discussed. Then, we elaborate on several representative physical mechanisms for structural color generation and the associated nanostructure designs. For the stacked multi-layer thin film design, various structural colors can be generated based on either FP resonance or photonic crystal resonance; for the subwavelength nanostructure array design, different colors can be created using an array of resonance effects including guided mode resonance, plasmon resonance, and Mie resonance. To speed up the metasurface design process, several optimization-based and machine learning-based intelligent inverse design algorithms have been exploited, which can greatly facilitate the optimized device design corresponding to a specific color generation target. Different fabrication techniques for large-scale and low-cost device manufacturing are subsequently explained, including electrochemical deposition, self-assembly, and nano-imprint lithography. Realization of dynamic colors can be achieved through incorporating active materials (e.g., liquid crystals, electrochromic polymers, and phase-change materials, etc.) into the device as well as utilizing external stimuli (e.g., bias voltage, heating, hydrogenation/oxidation treatment, etc.). Nanostructure-based structural colors have found widespread applications in color printing, information encryption, sensing, energy harvesting, etc.

Further development of the structural color technology calls for advances in several aspects. So far, a majority of device designs focus on achieving colors with high saturation and wide gamut coverage. While other important color characteristics such as brightness and hue, which play an equally important role in determining the generated colors’ visual appearance, have been paid less attention. In order to efficiently control and manipulate all three basic characteristics of a color, new design methodologies and intelligent algorithms need to be developed. Although several large-scale and low-cost manufacturing methods for structural colors have been demonstrated, precise and high-throughput methods that are compatible with various device constituent materials and structures, as well as different kinds of substrates (especially flexible ones) need to be exploited. Advances in dynamic color display with a wide tuning range and high spatial resolution require development in the device’s working mechanism, constituent materials, as well as associated fabrication techniques. Besides the applications surveyed in this article, structural colors might find niche applications in food and beauty makeup products. Moreover, integrating structural colors into existing optoelectronic devices and systems could further extend the application scenarios of structural color technology.
